# A two-phase study investigating the quality of life benefit of additional 0.5% cocaine mouthwash to institutional standard of care mucositis management in head and neck cancer patients undergoing radiotherapy or chemoradiotherapy

**DOI:** 10.1186/s12885-025-14955-7

**Published:** 2025-10-10

**Authors:** Piyush Grover, Colin Tang, Angela Jacques, Elizabeth Kernutt, Joshua Dass, Joanna Dewar, Rohen White, Annette M. Lim

**Affiliations:** 1 Sir Charles Gairdner Hospital, Perth, WA Australia; 2 Medical School, The University of Western Australia, Perth, WA Australia; 3 Department of Research, Sir Charles Gairdner Hospital, Hospital Avenue, Nedlands, WA Australia; 4 Institute for Health Research, The University of Notre Dame, Fremantle, WA Australia; 5 Peter MacCallum Cancer Centre, 305 Grattan St Melbourne, Melbourne, VIC 3000 Australia; 6 Sir Peter MacCallum Department of Oncology, The University of Melbourne, VIC Melbourne, Australia

**Keywords:** Mucositis, Cocaine mouthwash, Head and neck, Cancer, Chemotherapy, Radiotherapy

## Abstract

**Background:**

Mucositis remains a challenging complication of radiotherapy or chemoradiotherapy (CRT) for head and neck cancer (HNC) patients. There is limited data on the safety or effectiveness of cocaine mouthwash to treat mucositis.

**Methods:**

This was single centre, prospective sequential cohort study in HNC patients undergoing CRT. Patients were allocated to the standard of care mucositis management (SOC) or SOC + 0.5% CMW (CMW). We assessed the benefit of CMW in the management of mucositis on patient reported quality of life (QoL) using the EORTC QLQ-C30 and H&N35 questionnaires at baseline, during CRT, at 1- and 3-months follow-up.

**Results:**

Of 137 eligible patients,64 were included in each arm. Baseline characteristics were similar between the arms. Most patients were male (84%) who received 70 Gy of radiation (71%) with concurrent systemic therapy (74%). Up to 69% of patients had grade 2 or 3 mucositis and up to 78% of patients required prescription for opioid analgesia, but no consistent or clinically meaningful differences were observed between the arms. Morbidity was high without significant differences observed between SOC and CMW with high hospitalisation rates (49%) and need for enterostomy feeding (29%). There were significantly more patients with > 5% weight change from baseline to 3-months post treatment in the SOC (56.3% vs. 40.6%, *p* = 0.024). Rate of opioid analgesia prescription were not consistently different between the arms. No significant differences were observed in patient reported QoL between SOC and CMW (adjusted and unadjusted), or when assessed using a 10-point clinically meaningful difference threshold (*p* > 0.05).

**Conclusion:**

In HNC patients undergoing CRT, the addition of CMW to SOC management did not demonstrate any patient reported quality of life improvement or harm.

**Trial registration:**

The study was retrospectively registered with the Australian New Zealand Clinical Trials Registry (ACTRN12625000158460) on 11 February 2025.

**Supplementary Information:**

The online version contains supplementary material available at 10.1186/s12885-025-14955-7.

## Introduction

Mucositis is a common and challenging complication of radiotherapy or chemoradiotherapy (CRT) in head and neck cancer (HNC) patients. It occurs in at least 75% and up to 90% of patients with locally advanced mucosal head and neck cancers who receive radiotherapy with or without chemotherapy [1, 2]. The etiopathogenesis of mucositis in HNC patients is complex and involves the destruction of the basal epithelial mucosal layer and activation of multiple biological pro-inflammatory pathways leading to ulceration [3]. There are multiple patient and treatment related factors associated with development of mucositis in HNC patients such as alcohol and tobacco consumption, diabetes, low body mass index, unintentional weight loss prior to therapy, higher comorbidity burden, female gender, hyperfractionated accelerated radiation regimens, and concurrent anti-neoplastic systemic therapy [4]. Severe (grade 3 or 4) mucositis is reported to occur in 77–85% of such patients [5]. Mucositis may result in severe pain, malnutrition, secondary infections, and poorer quality of life (QoL) [2, 5, 6]. Complications of mucositis may lead to hospitalisations or treatment delays; the latter associated with inferior survival outcomes [7, 8]. The economic impact of mucositis is significant with high incremental hospitalisation costs in those with mucositis [9].

The publication of the updated Multinational Association of Supportive Care in Cancer/International Society of Oral Oncology (MASCC/ISOO) guidelines on the management of mucositis secondary to cancer therapy in 2020 provided a robust review of the literature on the agents available to prevent/treat oral mucositis in HNC patients undergoing CRT [10]. Several agents, such as benzydamine mouthwash, photobiomodulation, topical morphine mouthwash, sucralfate, growth factors and cytokines, have been examined to alleviate mucositis. However, there is limited high-level evidence to support the clinical use of the majority of these agents in the management of mucositis in HNC patients undergoing CRT. Benzydamine mouthwash is the only agent with level 1 evidence for the prevention of oral mucositis in HNC patients receiving moderate dose radiotherapy (< 50 Gy). There is limited objective data in the literature for the use of cocaine mouthwash for mucositis management, however it is routinely used in clinical practice in both the adult and paediatric population. The analgesic effect of cocaine is well established with the most widely accepted understanding of it having a similar mechanism of action to local anaesthetic agents, through the inhibition of action potential generation after binding to sodium channels in nociceptive receptors [11]. There are no prospective studies examining its safety or effectiveness in management of CRT induced mucositis. Hence, we sought to assess the benefit of the use of 0.5% cocaine mouthwash (CMW) to institutional standard of care (SOC) management of mucositis-related pain in HNC patients undergoing CRT.

## Methods

### Study design

This was a single centre, prospective sequential cohort study at a tertiary hospital in Perth, Western Australia between 1 August 2016 and 11 November 2019. The primary objective was to assess the benefit of the use of 0.5% CMW to institutional SOC management of mucositis-related pain in HNC patients undergoing CRT assessed by the validated European Organization for Research and Treatment of Cancer Quality of Life and Head and Neck 35 (EORTC QLQ-C30 and H&N35) questionnaires [12, 13]. Secondary objectives were (1) rate of analgesic prescription, (2) physician assessed mucositis score, (3) patient reported pain using an 11-point Numeric Rating Scale, (4) number of hospital admissions, and (5) assessment of weight.

The study was conducted pragmatically and focused on the global impact of the intervention rather than individual patient benefit. The first cohort of patients (the CMW arm) received analgesia prescribed according to the analgesic ladder guidelines including 0.5% CMW, and upon completion of the CMW arm, the standard arm only received medications as per the analgesia guideline without the CMW (the SOC arm).

### Ethics approval

Institutional ethics approval was sought (application number 12573) from the Sir Charles Gairdner Group Human Research Ethics Committee and informed patient consent was obtained prior to study participation. The study was performed in accordance with the applicable laws and regulations, good clinical practice, and ethical principles as described in the Declaration of Helsinki [14]. The study reported according to the Consolidated Standards of Reporting Trials (CONSORT) guideline and the results are reported as per the CONSORT statement [15].

### Patient eligibility

Eligible patients had a histologically confirmed HNC receiving CRT in the definitive or adjuvant setting. Patients were excluded if they were under 18 years of age, pregnant or breastfeeding, or allergic or intolerant to any of the study treatment intervention.

### Guideline development for mucositis management

The mucositis-related pain management guideline was established following consensus agreement between medical oncology, radiation oncology, palliative care and pain service teams and was disseminated after an educational session. Step 1 of the analgesic ladder focused on topical management. If the patient required topical therapy, all three of the following mouthwashes were provided – (1) benzydamine mouthwash, (2) lignocaine viscous 2% mouthwash, and (3) cocaine mouthwash 0.5% (in the CMW arm only). Each mouthwash was encouraged to be used regularly and staggered in use to maximise relief. Step 2 of the analgesic ladder employed the use of short acting analgesia with preference for morphine elixir or hydromorphone elixir on a when required basis. Step 3 focused on addition of long-acting analgesia with preference for long-acting oral morphine/oxycodone or fentanyl patch. Step 2 and step 3 could be initiated simultaneously. The guidelines recommended routine mouth care and oral hygiene. Patients were not blinded for the intervention. Further information on the analgesic ladder used in this study can be found as additional information in Supplementary Material 1. An a priori decision was not to mandate medication use diary from the patients given the already high burden of care and intervention in this patient group.

### Outcome assessments

Physician assessed and patient reported outcomes were undertaken at baseline, weekly during CRT and at 1-month and 3-month following completion of the treatment. Physician assessed outcomes included (1) grading of mucositis (as per the Common Terminology Criteria for Adverse Events version 4.03) (up-to-date version at the time of the study), (2) review of the analgesic requirements at each step of the analgesic ladder, (3) documentation of weight in kilograms, (4) capturing need for hospitalisation for any cause, and (5) capturing need for enterostomy tube. Patient reported outcomes included assessment of QoL using the EORTC QOL-C30 and H&N35 questionnaires and pain level using the visual analogue scale [16]. The final survival sweep was undertaken on 5 August 2020, and the database locked.

### Data aggregation

Data gathered at weekly treatment reviews was aggregated to cover missing data and because the available data between consecutive treatment weeks did not differ significantly. Data is thus reported at baseline, aggregated at week 1 and 2 of CRT, aggregated at week 4 and 5 of CRT, last week of CRT, 1-month post completion of CRT and 3-months post completion of CRT.

### Statistical analysis

The sample size was not formally calculated given the pragmatic conduct of the study. Based on the limited availability of the CMW, it was estimated that 30–50 patients would be recruited in each arm based on the total amount of CMW purchased for the conduct of the study and drug expiry date. A post-hoc analysis of the sample of size for the quality of life analyses demonstrated that a sample range of *n* = 80 to 128 had 80% power to detect a range of effect size differences f = 0.238 to 0.185 in a repeated measures mixed effects model between 2 groups over 10 timepoints with repeated measures correlations = 0.5 (based on mean difference (standard deviation range) = 3.0 (6.3 to 8.1) points in the EORTC QLQ-C30 Global summary scale score) (G*Power 3.1.9.7).

Descriptive summaries of patient demographic and clinical data and secondary outcome data consist of frequency distributions for categorical data and means and standard deviations or medians, interquartile ranges and ranges for numerical (continuous, ordinal and count) data. Normality of distribution of continuous variables were assessed graphically and by Shapiro-Wilk tests.

Univariate treatment group comparisons were done using Chi squared or Fisher exact tests, as appropriate depending on cell counts, for categorical data comparisons and Mann-Whitney U tests for continuous data comparisons. Generalised linear mixed models (GLMM), with appropriate canonical links, and random patient effects, were used to obtain predicted mean estimates of EORTC QLQ-C30 and H&N35 scale scores. Tukey ladder of powers (ladder of transformations) graphical assessments was used to examine outcome distribution shapes. Model fit was checked by graphical assessment of the normality distribution of residuals. Results were summarised as estimated marginal means and 95% confidence intervals (CI), within group mean (95% CI) differences from baseline and between group cross-sectional adjusted mean (95% CI) differences were reported. Models were adjusted for age, gender, American Joint Committee on Cancer (AJCC) 7th Edition Staging, Charlson Comorbidity Index, 5% weight decrease from baseline, current smoking and enterostomy. GLMM utilise maximum likelihood estimation methods, allowing all available data points for all patients to be included in models regardless of missing data. Common Terminology Criteria for Adverse Events v4.03 Oral Mucositis Grading ordinal outcome was recoded into mucositis indicator (yes/no) and severity (nil-mild/moderate-severe) categories and modelled using mixed effects logistic regression, with results summarised using odds ratios and 95% CI. Models were adjusted for age, gender, AJCC 7th Edition Staging, Charlson Comorbidity Index, 5% weight decrease from baseline, current smoking and enterostomy status. Mean differences in QoL scores were considered clinically relevant if a minimum discrepancy of 10 points was observed [17]. All hypotheses were 2-sided, and significance was set at alpha = 0.05. Stata version 18.0 (StataCorp, College Station, TX) and IBM SPSS version 28.0 were used for data analysis.

## Results

### Baseline characteristics

Of one-hundred and thirty-seven participants, 9 participants were excluded from the analyses set (four received palliative treatment, two withdrew from all study participation in the first two weeks of treatment, two did not have a mucosal HNC, and one did not receive radiation therapy). There were 64 participants in each arm for analysis (CONSORT diagram provided in Supplementary Material 2). The median follow-up for both arms was 27 months (range 2–48). The median follow-up in the CMW arm was 36 months (range 2–48). The median follow-up in the SOC arm was 19 months (range 4–42). As expected, the follow-up in the CMW group was longer given its initial allocation prior to commencement of the SOC arm. Baseline characteristics and treatment modalities are summarised in Table 1. None of the characteristics were statistically different between the two arms. Most patients (71%) received 70 Gy of radiation treatment, whilst the rest received 60–66 Gy of radiation treatment. The majority (> 96% in both arms) received intensity-modulated radiation therapy or volumetric modulated arc therapy. Most patients (74%) received concurrent systemic treatment, most commonly with cisplatin chemotherapy as a radiosensitiser.

**Table 1:** Baseline characteristics and treatment modalities between the 0.5% cocaine mouthwash arm and the standard of care arm

	Total, SOC + CMW (*n* = 128)	CMW (*n* = 64)	SOC (*n* = 64)	*p*-value^*^
Age (years), mean (SD)	63.2 (10.6)	63.4 (11.2)	62.9 (10.2)	0.785
Gender, n (%)				0.144
Male	108 (84.4)	51 (79.7)	57 (89.1)	
Female	20 (15.6)	13 (20.3)	7 (10.9)	
ECOG performance status, n (%)				0.927
0 or 1	114 (89.0)	57 (89.0)	57 (89.0)	
≥ 2	2 (1.6)	1 (1.6)	1 (1.6)	
Unknown	12 (9.4)	6 (9.4)	6 (9.4)	
Tumour location, n (%)				0.486
Oropharynx	66 (51.6)	29 (45.3)	37 (57.8)	
Nasopharyngeal cavity	7 (5.5)	4 (6.2)	3 (4.7)	
Oral Cavity	21 (16.4)	13 (20.3)	8 (12.5)	
Larynx	14 (10.9)	6 (9.4)	8 (12.5)	
Hypopharynx	5 (3.9)	4 (6.2)	1 (1.6)	
Unknown Primary	15 (11.7)	8 (12.5)	7 (10.9)	
Surgery performed prior to radiotherapy/chemoradiotherapy, n (%)	32 (25.0)	18 (28.1)	14 (21.9)	0.414
Smoking status, n (%)				0.810
Never	28 (21.9)	14 (21.9)	14 (21.9)	
Ex-smoker	69 (53.9)	33 (51.6)	36 (56.2)	
Current smoker	31 (24.2)	17 (26.6)	14 (21.9)	
Alcohol consumption –, n (%)				0.161
None or < 20 g/day	71 (55.5)	33 (51.6)	38 (59.4)	
Current > 20 g alcohol per day	35 (27.3)	20 (31.2)	15 (23.4)	
Past > 20 g alcohol per day	8 (6.2)	5 (7.8)	3 (4.7)	
Not known	14 (10.9)	6 (9.4)	8 (12.5)	
Charlson comorbidity index > 5, n (%)	25 (19.5)	16 (25.0)	9 (14.1)	0.119
Baseline weight (kg), mean (SD)	79.7 (17.5)	77.3 (19.3)	82.2 (15.4)	0.119
Disease stage, AJCC 7th edition, n (%)				0.134
Stage I	6 (4.7)	5 (7.8)	1 (1.6)	
Stage II	14 (10.9)	4 (6.2)	10 (15.6)	
Stage III	32 (25.0)	15 (23.4)	17 (26.6)	
Stage IVA/B	76 (59.4)	40 (62.5)	36 (56.2)	
Systemic treatment administration, n (%)	95 (74.2)	44 (68.8)	51 (79.7)	0.157
Radiotherapy delivery, n (%)				0.079
< 70 Gy	37 (28.9)	23 (35.9)	14 (21.9)	
70 Gy	91 (71.1)	41 (64.1)	50 (78.1)	

*arms were not randomised

*AJCC* American Joint Committee on Cancer, *CMW* 0.5% cocaine mouthwash arm, *ECOG* Eastern Cooperative Oncology Group, *IQR* Interquartile range, *SD* Standard deviation, *SOC* Standard of care arm

### Rate of mucositis and pain scores

The rate of any grade of mucositis did differ between the SOC and CMW arms at various time points but was difficult to interpret given differing incidences of the grade of mucositis between arms (Table 2). For example, at week 4–5 CRT, the CMW had more patients with no mucositis, less grade 1 mucositis but more grade 2 mucositis than SOC (*p* = 0.046). At 1-month post completion of CRT, the CMW had more patients with no mucositis and less patients with grade 1 mucositis than SOC (*p* = 0.008). As expected, grade 2 and 3 mucositis occurred with higher prevalence towards the last week of treatment, resolved with longer follow up, and were similar between the arms. Post-hoc adjusted logistic regression modelling for mucositis grade demonstrated lower odds for the CMW to have grade 1 mucositis towards the end of CRT (*p* = 0.012) and at 1-month post treatment (*p* = 0.017), and lower odds of grade 2 mucositis at the week 6–8 timepoint (*p* = 0.039) compared to SOC (Supplementary Material 3). Furthermore, adjusted logistic regression modelling of any mucositis versus no mucositis demonstrated lower odds of any mucositis at the week 6–8 timepoint (odds ratio 0.16, *p* = 0.047) and 3-month post radiation timepoint (odds ratio 0.08, *p* = 0.027) for the CMW compared to SOC (Supplementary Material 3). Although differences were noted between arms at different time points, these were not considered clinically meaningful given the co-occurrence of higher proportion of grade 2 mucositis in the CMW arm, and the absence of improvements in the other assessed variables such as pain levels or prescriptions. However, there were more patients with no mucositis in the CMW at most timepoints compared to SOC.

‘Severe pain’ scores (pain score ≥ 7) and ‘any pain’ scores (pain score > 0) reported on the Numeric Rating Scale were not statistically different between the CMW and SOC (*p* > 0.05), although numerically higher in the SOC arm in the last week of treatment (Table 3). Adjusted and unadjusted longitudinal pain scores (0–10) were not different between the treatment groups. Pain scores were higher towards the end of the treatment and improved post completion of treatment (Supplementary Material 4).

**Table 2:** Rate of mucositis over time in the cocaine mouthwash arm and the standard of care arm

Time point	Total (CMW, SOC), *n*	Grade of mucositis as per CTCAE v4.03	CMW, *n* (%)^#^	SOC, *n* (%)^#^	*p*-value^*^
Week 1–2	109 (59,50)				0.779
		0	45 (76.3)	40 (80.0)	
		1	11 (18.6)	9 (18.0)	
		2	2 (3.4)	1 (2.0)	
		3	1 (1.7)	0	
Week 4–5	119 (60,59)				0.046
		0	14 (23.3)	6 (10.2)	
		1	12 (20.0)	24 (40.7)	
		2	31 (51.7)	25 (42.4)	
		3	3 (5.0)	4 (6.8)	
Week 6–8	101 (52,49)				0.009
		0	11 (21.2)	2 (4.1)	
		1	5 (9.6)	14 (28.6)	
		2	21 (40.4)	23 (46.9)	
		3	15 (28.8)	10 (20.4)	
1-month follow-up	100 (47,53)				0.008
		0	32 (68.1)	25 (47.2)	
		1	6 (12.8)	22 (41.5)	
		2	7 (14.9)	6 (11.3)	
		3	2 (4.3)	0 (0.0)	
3-month follow-up	92 (49,43)				0.053
		0	45 (91.8)	34 (79.1)	
		1	1 (2.0)	7 (16.3)	
		2	3 (6.1)	2 (4.7)	
		3	0	0	

^#^percentages based on the sample size of the respective CMW or SOC cohort at the given timepoint

*arms were not randomised

*CMW *0.5% cocaine mouthwash arm,* CTCAE *Common Terminology Criteria for Adverse Events v4.03,* CRT *radiotherapy or chemoradiotherapy arm,* SOC *standard of care arm

**Table 3:** Longitudinal pain score categories - any pain (pain score > 0) and severe pain (pain score ≥ 7) in the cocaine mouthwash arm and the standard of care arm

Time point	Any pain (pain score > 0)			Severe pain (pain score ≥ 7)		
	CMW, *n* (%)	SOC, *n* (%)	*p*-value^*^	CMW, *n* (%)	SOC, *n* (%)	*p*-value^*^
Week 1–2	16 (27.1)	17 (32.7)	0.521	2 (3.4)	0 (0.0)	0.497
Week 4–5	45 (77.6)	49 (84.5)	0.343	3 (5.2)	2 (3.4)	1.000
Week 6–8	42 (82.4)	48 (94.1)	0.065	6 (11.8)	9 (17.6)	0.402
1-month follow-up	21 (43.8)	27 (50.9)	0.470	0	0	Not applicable
3-month follow-up	13 (27.7)	13 (29.5)	0.842	0	0	Not applicable

*arms were not randomised

*CMW*- 0.5% cocaine mouthwash arm, *SOC* standard of care arm

### Morbidity associated with CRT

Hospitalisations due to any cause occurred in 63/128 (49%) of all patients with the median of 7 days in both arms for the maximum length of stay, highlighting the significant morbidity in patients with HNC undergoing CRT (Table 4). The median number of hospitalisations in CMW was 2 (IQR 1,3) and in the SOC was 1 (IQR 1,1) (*p* = 0.017). An enterostomy tube was required in 29% of patients. However, this was not statistically different between the treatment arms although numerically higher in the CMW arm, noting more patients in the SOC received 70 Gy dose of radiation treatment with concurrent systemic treatment. Weight loss occurred in both arms and the median weight loss was 9.8 kg and 9.9 kg at the 3-month follow-up period in SOC and CMW arms respectively. Of note, despite similar enterostomy tube rates which were numerically higher in the CMW arm, the proportion of patients with > 5% weight change from baseline to 3-months post treatment, was significantly higher in the SOC (56.3% vs. 40.6%, *p* = 0.024).

**Table 4:** Hospitalisation rate, hospitalisation length of stay, enterostomy feeding tube rate, treatment interruption rate and > 5% weight loss at 3-month follow-up in the cocaine mouthwash arm and the standard of care arm

Variable	Total (CMW + SOC)	CMW	SOC	*p*-value^*^
Hospitalisation, n (%)	63 (49.2)	34 (53.1)	29 (45.3)	0.377
Maximum length of hospitalisation stay (days), median (IQR)	7 (4,17)	7 (4,23)	7 (4,14)	0.572
Enterostomy feeding tube, n (%)	37 (28.9)	23 (35.9)	14 (21.9)	0.079
> 5% weight change at 3-month follow-up compared to baseline, n (%)	62 (48.4)	26 (40.6)	36 (56.3)	0.024
Interruption to systemic therapy, n (%)	23 (18.0)	10 (15.6)	13 (20.3)	0.583
Interruption to radiotherapy, n (%)	12 (9.4)	9 (14.1)	3 (4.7)	0.069

*arms were not randomised

*CMW* 0.5% cocaine mouthwash arm, *IQR* interquartile range, *SOC* standard of care arm

### Rate of analgesia prescription

Similar to the observation that there was no difference between arms in patient reported pain scores, the rate of analgesia prescription at steps 1 and 3 of the analgesic ladder also did not consistently differ significantly between the two arms (Table 5). At week 4–5 of CRT the rate of Step 2 analgesia (short acting PRN opioid analgesia) prescription was higher in the CMW compared to SOC (66.1% vs. 43.3%, *p* = 0.018). The higher grade 2 mucositis in the CMW at this same timepoint corresponds to the higher rate of step 2 short acting PRN opioid analgesia, even though the grade 1 mucositis was lower, and no mucositis was higher in the CMW at this timepoint. Therefore, this solitary statistically significant difference should be interpreted with caution. Unsurprisingly, the rate of analgesia prescription was highest during the last week of CRT and subsequently dropped with longer follow-up.

**Table 5:** Rates of analgesic prescription use in the cocaine mouthwash arm and the standard of care arm

Time point	Total, (CMW, SOC), *n*	Analgesic requirement as per the analgesic ladder	CMW, *n* (%)^#^	SOC, *n* (%)^#^	*p*-value^*^
Week 1–2	111 (59,52)				
		Step 1^^^	22 (37.3)	14 (26.9)	0.244
		Step 2^^^^	10 (16.9)	3 (5.8)	0.082
		Step 3^^^^^	4 (6.8)	0 (0)	0.121
Week 4–5	122 (62,60)				
		Step 1^^^	53 (85.5)	53 (88.3)	0.641
		Step 2^^^^	41 (66.1)	26 (43.3)	0.018
		Step 3^^^^^	20 (32.3)	16 (26.7)	0.498
Week 6–8	106 (55,51)				
		Step 1^^^	49 (89.1)	47 (92.2)	0.589
		Step 2^^^^	34 (63.0)	25 (49.0)	0.172
		Step 3^^^^^	27 (50.0)	23 (45.1)	0.615
1-month follow-up	100 (48,52)				
		Step 1^^^	24 (50.0)	19 (36.5)	0.174
		Step 2^^^^	11 (22.9)	7 (13.5)	0.219
		Step 3^^^^^	11 (22.9)	10 (19.2)	0.612
3-month follow-up	94 (49,45)				
		Step 1^^^	12 (24.5)	7 (15.6)	0.268
		Step 2^^^^	2 (4.1)	1 (2.2)	1.000
		Step 3^^^^^	6 (12.2)	2 (4.4)	0.271

^#^Percentages based on the sample size of the respective CMW or SOC cohort at the given timepoint

^^^Step 1 - topical analgesic management

^^^^Step 2 - short acting opioid analgesia on a when required basis

^^^^^Step 3 - long-acting analgesia

*arms were not randomised

*CMW*- 0.5% cocaine mouthwash arm*, CRT* radiotherapy or chemoradiotherapy arm, *SOC* standard of care arm

### Quality of life assessment

**Fig. 1:**
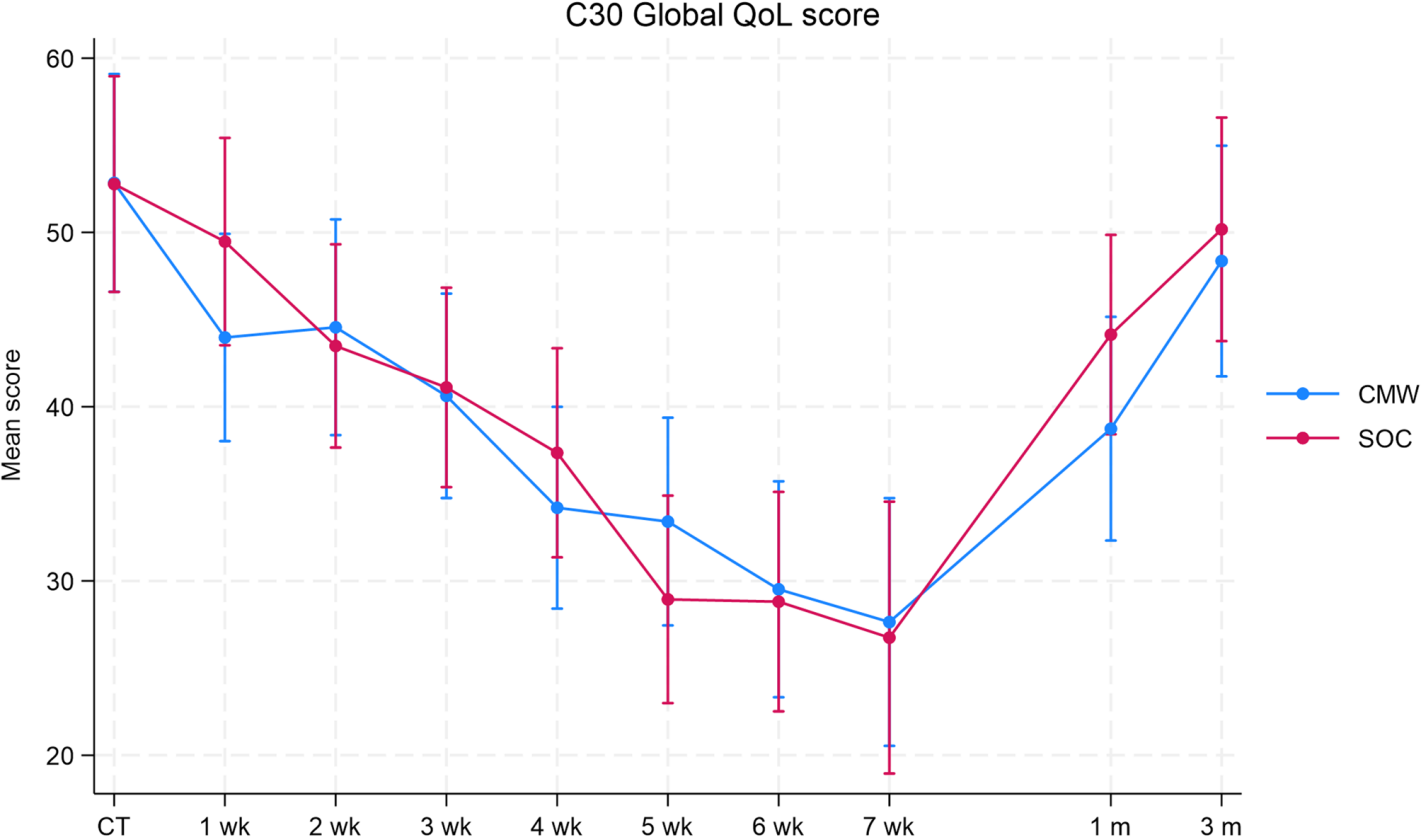
Mean global health status scores assessed by the EORTC QLQ-C30 questionnaire at each timepoint of assessment according to treatment. *CMW *0.5% cocaine mouthwash arm; *CT *commencement of treatment; *m *follow-up month; *SOC *standard of care arm; *wk* week of treatment

Quality of life was assessed using the EORTC QLQ-C30 and H&N 35 questionnaires. Compliance for completion of the 65 questions on both questionnaires was reasonable. From baseline to week 6 of treatment, the median compliance was 70%, but in week 7, reflecting the peak of toxicity, compliance dropped to 39% but returned to 70% and 55% at months 1 and 3 follow-up, respectively. No statistically significant differences were noted consistently between the treatment arms for any domain assessed by the two QoL questionnaires at any time point, using unadjusted and adjusted analyses which considered the baseline value (Supplementary Material 5), or using a mean 10-point difference to define clinically meaningful change. Figure 1 demonstrates the global health status on the EORTC QLQ-C30 questionnaire demonstrating no difference in the mean QoL scores at any of the assessed time points. In the last week of CRT, the mean reduction in global QoL score on the EORTC QLQ-C30 was greatest at 25.2 points. Following that, the mean scores improved at 1- and 3-month follow-up, however, reached the baseline levels at the last follow-up.

A summary of results including categorisation by 10-point reduction in scores, for both questionnaires is provided as additional information in Supplementary Material 5. For some domains at specific time points, statistically significant differences were observed between arms, such as on the EORTC QLQ-C30 pain domain, 10-point difference assessment at week 7 (p = 0.044). However, this was not reflected by similar changes in other domains of the same questionnaire, or on the H&N 35 questionnaire pain domains or relevant other domains such as “pain killers”, or in other outcome measures such as pain reporting using the visual analogue scale or by physician assessed toxicity grading or medication prescription. Overall, there were no consistent or significant differences in patient reported outcomes assessed by either questionnaire noted between the treatment arms using multiple methods of analyses.

## Discussion

This was a prospective study investigating the addition of 0.5% CMW to institutional standard of care guidelines in management of mucositis in HNC patients receiving CRT. We did not find clear evidence of benefit or harm from addition of CMW to SOC guidelines on physician assessed measures or patient reported outcomes.

Our patient cohort is largely representative of the patient population with HNC. In the Eating as Treatment (EAT) TROG (Trans-Tasman Radiation Oncology Group) 12.03 study that investigated the efficacy of ‘Eating as Treatment’ program in 307 participants undergoing definite or post-operative radiotherapy or chemoradiotherapy for head and neck cancers across six high-volume head and neck cancer centres in Australia, the baseline patient cohort characteristics were similar to the current study [18]. The majority of the patients were male (84.4%) with stage IV disease (59.4%) and oropharynx as the most common site of disease (51.6%). Concurrent chemoradiotherapy was received by 74.2% of patients, mainly with concurrent platinum-based chemotherapy (58.6%) and 71.1% of patients received 70 Gy of radiation. In a prospective, multicentre, non-interventional study in 75 patients across 6 US centres, baseline patient characteristics were also similar [19].

There is variation in the reported incidence of mucositis in the literature given the heterogeneity of reporting and the scoring criteria. In our study, up to 69.2% of patients had grade 2 or 3 mucositis and up to 78% patients required prescription for an opioid analgesia. In the DAHANCA 6&7 randomised trial with accelerated radiotherapy for head and neck cancer, the incidence of moderate/severe mucositis varied from 47% to 84% [20]. Their assessment was based on a pragmatic weekly scoring system of morbidity in HNC patients by Dische et al. [21, 22]. The rate of opioid prescription for pain management was between 37% and 79%.

Our study further confirms that the morbidity associated with mucositis in HNC patients undergoing CRT is significant as demonstrated by the number of hospitalisations (45.3–53.1% of patients), associated maximum median length of stay of 7 days, and need for enterostomy tube (21.9–35.6% of patients). In the previously mentioned US study across 6 centres, 85% of patients had mucositis associated pain and functional impairment despite use of opioid analgesia, 37% were hospitalised (33% of which were deemed to be secondary to mucositis), the mean length of hospital admission was 4.9 days and 51% of patients required enteral tube feeds [19]. In a retrospective review of Surveillance, Epidemiology, and End Results (SEER) cancer registry records of head and neck cancer patients over the age of 65 years undergoing treatment with CRT between 2001 and 2009, up to 62% of patients presented to the emergency department or required hospitalisation with an acute treatment related toxicity and up to 74% of patients required enteral tube feeding [22]. Previous reports have shown similar rates of mucositis related hospitalisations in younger and older patients receiving chemoradiotherapy [23, 24].

Weight loss is common and multifactorial in HNC patients receiving CRT [25]. Patients have been reported to lose up to 12% of pre-treatment body weight and significantly, weight loss is associated with increased morbidity and mortality [26–32]. In this cohort of patients, unintended weight loss > 5% during radiotherapy is considered significant [33]. Of all patients, 48.4% of all patients reported > 5% weight loss at 3-month follow-up compared to their baseline weight, highlighting the importance of minimising weight loss in this patient cohort. There was a statistically significant difference in the rate of > 5% weight loss between the arms at the 3-month follow-up (56.3% in SOC vs. 40.6% in CMW, *p* = 0.024), however, this finding was not corroborated with consistent or clinically meaningful differences in rate of mucositis, analgesia prescription or QoL scores between the arms.

In this study there was no evidence of benefit from addition of CMW to standard of care guidelines on global or any of the individual QoL metrics on the two QoL questionnaires. There has been no prior evaluation of the impact of CMW on QoL metrics in management of mucositis in HNC patients. As expected, there was a reduction in the QoL scores during treatment with CRT. There is consensus that mucositis significantly impedes QoL and functional status in HNC patients given the associated pain and impairment in swallowing, chewing, drinking and speaking [5, 7, 34, 35]. Whilst basic oral care is recommended in management of mucositis and may improve QoL scores, there is no robust evidence for pharmacological agents to improve QoL [36]. Our results, once adjusted for other variables, suggested that there may be higher odds of lower grade mucositis in CMW arm at a few timepoints during treatment with CRT. However, these findings were not consistent with other assessed variables and should be interpreted cautiously given the absence of improvement in reported pain, QoL scores and analgesia prescription at similar time points, and with there being no statistically or clinically significant differences observed between treatment arms for any of the outcomes investigated. Preliminary results from the phase 3 study of avasopasem manganese to reduce incidence of severe oral mucositis in patients with locally advanced nonmetastatic HNC have been promising, however, this study did not assess patient reported QoL metrics [37]. Results from the phase IV clinical study, investigating feasibility of benzydamine oromucosal solution in management of radiation-induced mucositis in HNC patients are pending, which includes QoL assessment as a secondary end-point (NCT05055726).

Mucositis management remains an area of unmet need. There is limited high-level evidence to guide its appropriate management given insufficient or conflicting evidence and hence several recommendations are based on expert opinion [10]. The 2020 update of MASCC/ISOO guidelines on management of mucositis secondary to cancer therapy included benzydamine mouthwash as the only pharmacological agent with level 1 evidence in prevention of mucositis in head and neck cancer patients receiving moderate dose radiotherapy (< 50 Gy). However, it is not known whether benzydamine mouthwash use improves QoL in head and neck cancer patients with mucositis [38]. Up to 71.1% of patients in this study received high-dose radiation treatment (70 Gy) with systemic therapy. A systematic review of low-cost interventions to treat radiation induced mucositis in head and neck cancer patients identified benzydamine hydrochloride mouth rinse, honey and oral glutamine as potential therapeutics [39]. Whilst only glutamine was recommended to mitigate radiation induced mucositis, the lack of its standardised formulation and dose have precluded its robust assessment. Other local anaesthetic and analgesic agents, such as amitriptyline and doxepin mouthwashes which are believed to block conduction of local sodium channels, have shown some evidence of benefit [40, 41]. Doxepin mouthwash (5 mg/mL) has been shown to reduce the frequency of mucositis and severity of pain in HNC patients undergoing CRT in a multicentre, randomised, double-blind, placebo-controlled trial with a crossover phase [40]. The area under the curve for mouth and throat pain reduction was greater for doxepin than for placebo (*n* = 155; −9.1 vs. −4.7; *p* < 0.001). However, the study did not report QoL outcomes and there were multiple side effects associated with doxepin mouthwash that may impede its use in clinical care. Other anti-inflammatory agents such as celecoxib, irsogladine maleate, misoprostol and rebamipide have been investigated in management of mucositis and to date there is limited evidence to support their use in clinical practice [10]. There remains a need for high-quality studies to generate more robust evidence in mucositis management.

This study has strengths and limitations. It was not a randomised controlled trial and there were modest patient numbers in each cohort. The patient population examined was representative of typical head and neck cancer patient cohorts. There was no assessment of patient compliance to the analgesic guidelines and pain diary was not mandated. Nonetheless, we undertook a practical approach at assessment of an important clinical question that has not been addressed before. Further research is required to improve management of mucositis in head and neck cancer patients undergoing treatment.

In conclusion, this study did not demonstrate QoL benefit or harm from addition of CMW to SOC guidelines in the management of mucositis in HNC patients undergoing CRT.

## Supplementary Information

Supplementary Material 1.

Supplementary Material 2.

Supplementary Material 3.

Supplementary Material 4.

Supplementary Material 5.

## Data Availability

The datasets used and/or analysed during the current study available from the corresponding author on reasonable request.
