# Supplementary material for: A two-phase study investigating the quality of life benefit of additional 0.5% cocaine mouthwash to institutional standard of care mucositis management in head and neck cancer patients undergoing radiotherapy or chemoradiotherapy
Source: BMC Cancer. 2025 Oct 10;25:1551. doi: 10.1186/s12885-025-14955-7 (PMC12513096; doi:10.1186/s12885-025-14955-7)
Supplement: Supplementary file 1 — Supplementary Material 1. [file 12885_2025_14955_MOESM1_ESM.docx]

Supplementary table – Online Resource 1

*Table 1. Analgesic ladder for management of mucositis.*
