# Supplementary material for: A two-phase study investigating the quality of life benefit of additional 0.5% cocaine mouthwash to institutional standard of care mucositis management in head and neck cancer patients undergoing radiotherapy or chemoradiotherapy
Source: BMC Cancer. 2025 Oct 10;25:1551. doi: 10.1186/s12885-025-14955-7 (PMC12513096; doi:10.1186/s12885-025-14955-7)
Supplement: Supplementary file 2 — Supplementary Material 2. [file 12885_2025_14955_MOESM2_ESM.doc]

**A two-phase study investigating the quality of life benefit of additional 0.5% cocaine mouthwash to institutional standard of care mucositis management in head and neck cancer patients undergoing radiotherapy or chemoradiotherapy.**

Assessed for eligibility

(n=137)

**Enrolment**

Excluded (n=9)

Palliative intent treatment (n=4)

Withdrew consent (n=2)

Not mucosal head and neck cancer (n=2)

No radiation therapy received (n=1)

Allocated (n=128)

**Allocation**

#

**Follow up**

**Analysis**

Allocated to 0.5% cocaine mouthwash arm

(n =64)

Allocated to institutional standard of care

(n=64)

Analysed in the primary analysis (n=64)

Analysed in the primary analysis (n=64)

Lost to follow up at censor date (n=1)

Lost to follow up at censor date (n=0)
