# Supplementary material for: A two-phase study investigating the quality of life benefit of additional 0.5% cocaine mouthwash to institutional standard of care mucositis management in head and neck cancer patients undergoing radiotherapy or chemoradiotherapy
Source: BMC Cancer. 2025 Oct 10;25:1551. doi: 10.1186/s12885-025-14955-7 (PMC12513096; doi:10.1186/s12885-025-14955-7)
Supplement: Supplementary file 3 — Supplementary Material 3. [file 12885_2025_14955_MOESM3_ESM.docx]

**Additional File 3**

Logistic regression adjusted model results for mucositis grade outcomes (reference grade=0)

| Time | Grade 1 |  | Grade 2 |  | Grade 3 |  |
| --- | --- | --- | --- | --- | --- | --- |
|  | adjOR* (95%CI) | p-value | adjOR* (95%CI) | p | adj | p-value |
| Week 1-2 | 1.29 (0.32, 5.18) | 0.722 | - | - | - | - |
| Week 4-5 | 0.41 (0.08, 2.16) | 0.292 | 1.13 (0.22, 5.87) | 0.883 | 0.23 (0.01, 6.05) | 0.380 |
| Week 6-8 | 0.05 (0.01, 0.52) | 0.012 | 0.13 (0.02, 0.90) | 0.039 | 0.84 (0.09, 7.51) | 0.875 |
| 1-month follow-up | 0.15 (0.03, 0.71) | 0.017 | 3.44 (0.45, 26.40) | 0.235 | - | - |
| 3-month follow-up | 0.00 (0.00, 0.00) | 0.994 | 0.00 (0.00, 0.00) | 0.253 | - | - |

** Odds of CMW group compared to SOC group*

Mucositis severity categories (nil/mild vs mod/severe) by treatment group

| Time |  | CMW | SOC |  |
| --- | --- | --- | --- | --- |
|  |  | n (%) | n (%) | p-value |
|  |  |  |  |  |
| Week 1-2 | Nil-mild (0-1) | 56 (94.9%) | 49 (98.0%) | 0.623 |
|  | Moderate-severe (2-3) | 3 (5.1%) | 1 (2.0%) |  |
| Week 4-5 | Nil-mild (0-1) | 26 (43.3%) | 30 (50.8%) | 0.412 |
|  | Moderate-severe (2-3) | 34 (56.7%) | 29 (49.2%) |  |
| Week 6-8 | Nil-mild (0-1) | 16 (30.8%) | 16 (32.7%) | 0.839 |
|  | Moderate-severe (2-3) | 36 (69.2%) | 33 (67.3%) |  |
| 1-month follow-up | Nil-mild (0-1) | 38 (80.9%) | 47 (88.7%) | 0.401 |
|  | Moderate-severe (2-3) | 9 (19.1%) | 6 (11.3%) |  |
| 3-month follow-up | Nil-mild (0-1) | 46 (93.9%) | 41 (95.3%) | 1.000 |
|  | Moderate-severe (2-3) | 3 (6.1%) | 2 (4.7%) |  |

** Odds of CMW group compared to SOC group*

Logistic regression model results for any mucositis (reference no mucositis)

| Time | OR* (95%CI) | p-value | adjOR* (95%CI) | p-value |
| --- | --- | --- | --- | --- |
| Week 1-2 | 1.24 (0.50, 3.11) | 0.640 | 2.55 (0.76, 8.62) | 0.132 |
| Week 4-5 | 0.37 (0.13, 1.05) | 0.061 | 0.44 (0.11, 1.71) | 0.234 |
| Week 6-8 | 0.16 (0.03, 0.76) | 0.021 | 0.16 (0.03, 0.98) | 0.047 |
| 1-month follow-up | 0.42 (0.19, 0.95) | 0.037 | 0.59 (0.22, 1.59) | 0.295 |
| 3-month follow-up | 0.34 (0.10, 1.18) | 0.089 | 0.08 (0.01, 0.75) | 0.027 |

** Odds of CMW group compared to SOC group*

Logistic regression model results for moderate/severe mucositis (reference nil/mild mucositis)

| Time | OR* (95%CI) | p-value | adjOR* (95%CI) | p-value |
| --- | --- | --- | --- | --- |
| Week 1-2 | 2.63 (0.26, 26.06) | 0.410 | - | - |
| Week 4-5 | 1.35 (0.66, 2.79) | 0.412 | 1.09 (0.43, 2.77) | 0.863 |
| Week 6-8 | 1.09 (0.47, 2.52) | 0.839 | 1.09 (0.39, 3.05) | 0.864 |
| 1-month follow-up | 1.86 (0.61, 5.68) | 0.279 | 2.86 (0.64, 12.86) | 0.170 |
| 3-month follow-up | 1.34 (0.21, 8.40) | 0.757 | 0.12 (0.00, 4.09) | 0.239 |

** Odds of CMW group compared to SOC group*
