# Supplementary material for: A two-phase study investigating the quality of life benefit of additional 0.5% cocaine mouthwash to institutional standard of care mucositis management in head and neck cancer patients undergoing radiotherapy or chemoradiotherapy
Source: BMC Cancer. 2025 Oct 10;25:1551. doi: 10.1186/s12885-025-14955-7 (PMC12513096; doi:10.1186/s12885-025-14955-7)
Supplement: Supplementary file 4 — Supplementary Material 4. [file 12885_2025_14955_MOESM4_ESM.docx]

***Table****. Pain scores as per Numeric Rating Scale in the cocaine mouthwash arm and the standard of care arm.*

| Time point | Total, (CMW, SOC), n | Pain score category | CMW, n (%)^#^ | SOC, n (%)^#^ | p-value^*^ |
| --- | --- | --- | --- | --- | --- |
|  |  |  |  |  |  |
| Week 1-2 | 111 (59,52) |  |  |  | 0.361 |
|  |  | 0 = Nil | 43 (72.9%) | 35 (67.3%) |  |
|  |  | 1-3 = Mild | 11 (18.6%) | 15 (28.8%) |  |
|  |  | 4-6 = Moderate | 3 (5.1%) | 2 (3.8%) |  |
|  |  | ≥7 = Severe | 2 (3.4%) | 0 |  |
|  |  |  |  |  |  |
| Week 4-5 | 116 (58,58) |  |  |  | 0.743 |
|  |  | 0 = Nil | 13 (22.4%) | 9 (15.5%) |  |
|  |  | 1-3 = Mild | 26 (44.8%) | 30 (51.7%) |  |
|  |  | 4-6 = Moderate | 16 (27.6%) | 17 (29.3%) |  |
|  |  | ≥7 = Severe | 3 (5.2%) | 2 (3.4%) |  |
|  |  |  |  |  |  |
| Week 6-8 | 102 (51,51) |  |  |  | 0.284 |
|  |  | 0 = Nil | 9 (17.6%) | 3 (5.9%) |  |
|  |  | 1-3 = Mild | 21 (41.2%) | 24 (47.1%) |  |
|  |  | 4-6 = Moderate | 15 (29.4%) | 15 (29.4%) |  |
|  |  | ≥7 = Severe | 6 (11.8%) | 9 (17.6%) |  |
|  |  |  |  |  |  |
| 1-month follow-up | 101 (48,53) |  |  |  | 0.240 |
|  |  | 0 = Nil | 27 (56.3%) | 26 (49.1%) |  |
|  |  | 1-3 = Mild | 15 (31.3%) | 24 (45.3%) |  |
|  |  | 4-6 = Moderate | 6 (12.5%) | 3 (5.7%) |  |
|  |  | ≥7 = Severe | 0 | 0 |  |
|  |  |  |  |  |  |
| 3-month follow-up | 91 (47,44) |  |  |  | 0.044 |
|  |  | 0 = Nil | 34 (72.3) | 31 (70.5) |  |
|  |  | 1-3 = Mild | 8 (17.0) | 13 (29.5) |  |
|  |  | 4-6 = Moderate | 5 (10.6) | 0 |  |
|  |  | ≥7 = Severe | 0 | 0 |  |

**arms were not randomised; ^#^ percentages based on the sample size of the respective CMW or SOC cohort at the given timepoint; CMW- 0.5% cocaine mouthwash arm; SOC- standard of care arm.*
