# Supplementary material for: A two-phase study investigating the quality of life benefit of additional 0.5% cocaine mouthwash to institutional standard of care mucositis management in head and neck cancer patients undergoing radiotherapy or chemoradiotherapy
Source: BMC Cancer. 2025 Oct 10;25:1551. doi: 10.1186/s12885-025-14955-7 (PMC12513096; doi:10.1186/s12885-025-14955-7)
Supplement: Supplementary file 5 — Supplementary Material 5. [file 12885_2025_14955_MOESM5_ESM.docx]

**Additional File 5**

**EORTC QLQ-C30**

Table 1: C30 global scale score

|  | CMW (Tx) | | | SoC (Cx) | | |  |  |  |
| --- | --- | --- | --- | --- | --- | --- | --- | --- | --- |
| Time | Est mean (95%CI) | Mean change from baseline (95%CI) | p | Est mean (95%CI) | Mean change from baseline (95%CI) | p | Adjusted mean (95%CI) group difference | p# | P* |
| 0 | 52.84 (46.60, 59.09) |  |  | 52.78 (46.59, 58.96) |  |  | -1.89 (-11.16, 7.38) | 0.689 |  |
| 1 | 43.97 (38.02, 49.92) | -8.87 (-15.69, -2.05) | 0.011 | 49.48 (43.53, 55.43) | -3.30 (-10.04, 3.44) | 0.337 | 2.05 (-7.04, 11.14) | 0.659 | 0.446 |
| 2 | 44.56 (38.37, 50.75) | -8.29 (-15.29, -1.28) | 0.020 | 43.49 (37.65, 49.32) | -9.29 (-15.95, -2.63) | 0.006 | -0.27 (-9.53, 9.00) | 0.955 | 0.756 |
| 3 | 40.63 (34.76, 46.49) | -12.22 (-18.94, -5.50) | <0.001 | 41.11 (35.39, 46.83) | -11.67 (-18.22, -5.11) | <0.001 | 2.11 (-6.62, 10.85) | 0.635 | 0.424 |
| 4 | 34.20 (28.41, 39.99) | -18.64 (-25.31, -11.97) | <0.001 | 37.35 (31.36, 43.35) | -15.42 (-22.21, -8.63) | <0.001 | 5.53 (-3.38, 14.44) | 0.224 | 0.145 |
| 5 | 33.41 (27.45, 39.37) | -19.43 (-26.21, -12.66) | <0.001 | 28.94 (22.99, 34.90) | -23.83 (-30.58, -17.08) | <0.001 | -2.84 (-11.77, 6.10) | 0.534 | 0.854 |
| 6 | 29.52 (23.33, 35.72) | -23.32 (-30.29, -16.35) | <0.001 | 28.81 (22.52, 35.11) | -23.96 (-31.01, -16.92) | <0.001 | -1.56 (-10.92, 7.80) | 0.744 | 0.950 |
| 7 | 27.65 (20.54, 34.76) | -25.20 (-32.99, -17.41) | <0.001 | 26.75 (18.95, 34.55) | -26.03 (-34.49, -17.56) | <0.001 | -0.77 (-11.97, 10.44) | 0.893 | 0.855 |
| 1m | 38.74 (32.32, 45.16) | -14.11 (-21.33, -6.88) | <0.001 | 44.14 (38.42, 49.86) | -8.64 (-15.17, -2.10) | 0.010 | 6.63 (-2.56, 15.83) | 0.158 | 0.103 |
| 3m | 48.36 (41.74, 54.98) | -4.48 (-11.84, 2.88) | 0.233 | 50.18 (43.76, 56.59) | -2.60 (-9.77, 4.58) | 0.478 | 0.98 (-8.83, 10.80) | 0.844 | 0.601 |

*P# adjusted cross-sectional group differences : Adjusted for Age Gender Staging_AJCC_7th_edition CCI weight_5pct smoking_current Enterostomy*

*P* group-time interactions ie rate of change over time/difference of differences*

Table 2: C30 Physical functioning

|  | CMW (Tx) | | | SoC (Cx) | | |  |  |  |
| --- | --- | --- | --- | --- | --- | --- | --- | --- | --- |
| Time | Est mean (95%CI) | Mean change from baseline (95%CI) | p | Est mean (95%CI) | Mean change from baseline (95%CI) | p | Adjusted mean (95%CI) group difference | p# | P* |
| 0 | 84.10 (77.19, 91.00) |  |  | 86.70 (79.85, 93.55) |  |  | 0.09 (-10.44, 10.62) | 0.986 |  |
| 1 | 81.84 (75.23, 88.45) | -2.26 (-9.38, 4.87) | 0.535 | 82.26 (75.64, 88.87) | -4.44 (-11.49, 2.60) | 0.216 | -3.57 (-13.91, 6.77) | 0.498 | 0.520 |
| 2 | 80.89 (74.04, 87.75) | -3.20 (-10.52, 4.12) | 0.391 | 85.02 (78.52, 91.53) | -1.68 (-8.63, 5.28) | 0.636 | 4.04 (-6.48, 14.55) | 0.452 | 0.494 |
| 3 | 78.19 (71.66, 84.73) | -5.90 (-12.92, 1.12) | 0.099 | 81.95 (75.56, 88.35) | -4.75 (-11.59, 2.09) | 0.174 | 0.57 (-9.39, 10.53) | 0.911 | 0.931 |
| 4 | 69.57 (63.12, 76.03) | -14.52 (-21.49, -7.56) | <0.001 | 71.58 (64.91, 78.25) | -15.12 (-22.21, -8.03) | <0.001 | -0.02 (-10.17, 10.12) | 0.996 | 0.984 |
| 5 | 71.10 (64.47, 77.72) | -13.00 (-20.07, -5.92) | <0.001 | 66.18 (59.55, 72.80) | -20.53 (-27.58, -13.48) | <0.001 | -8.94 (-19.11, 1.24) | 0.085 | 0.108 |
| 6 | 64.88 (58.02, 71.73) | -19.22 (-26.50, -11.94) | <0.001 | 66.60 (59.64, 73.56) | -20.10 (-27.46, -12.74) | <0.001 | -4.58 (-15.20, 6.04) | 0.398 | 0.423 |
| 7 | 61.13 (53.37, 68.90) | -22.96 (-31.10, -14.82) | <0.001 | 62.76 (54.29, 71.22) | -23.94 (-32.79, -15.09) | <0.001 | -0.04 (-12.64, 12.56) | 0.995 | 0.985 |
| 1m | 71.55 (64.47, 78.63) | -12.55 (-20.10, -5.00) | 0.001 | 73.16 (66.76, 79.55) | -13.54 (-20.37, -6.72) | <0.001 | -1.64 (-12.09, 8.81) | 0.759 | 0.764 |
| 3m | 70.20 (62.92, 77.48) | -13.90 (-21.59, -6.21) | <0.001 | 85.23 (78.15, 92.30) | -1.47 (-8.97, 6.02) | 0.700 | 12.06 (0.94, 23.17) | 0.033 | 0.048 |

Table 3: C30 Role functioning

|  | CMW (Tx) | | | SoC (Cx) | | |  |  |  |
| --- | --- | --- | --- | --- | --- | --- | --- | --- | --- |
| Time | Est mean (95%CI) | Mean change from baseline (95%CI) | p | Est mean (95%CI) | Mean change from baseline (95%CI) | p | Adjusted mean (95%CI) group difference | p# | P* |
| 0 | 74.19 (65.45, 82.93) |  |  | 80.24 (71.58, 88.90) |  |  | 6.85 (-6.19, 19.90) | 0.303 |  |
| 1 | 65.24 (56.90, 73.59) | -8.95 (-18.15, 0.25) | 0.057 | 72.96 (64.61, 81.31) | -7.28 (-16.37, 1.81) | 0.117 | 7.56 (-5.25, 20.38) | 0.247 | 0.919 |
| 2 | 67.54 (58.88, 76.20) | -6.65 (-16.10, 2.80) | 0.168 | 71.05 (62.85, 79.25) | -9.19 (-18.17, -0.21) | 0.045 | 7.71 (-5.32, 20.74) | 0.246 | 0.904 |
| 3 | 68.55 (60.31, 76.79) | -5.64 (-14.70, 3.42) | 0.222 | 68.11 (60.05, 76.16) | -12.13 (-20.97, -3.29) | 0.007 | -1.77 (-14.12, 10.58) | 0.779 | 0.203 |
| 4 | 49.71 (41.57, 57.85) | -24.48 (-33.48, -15.48) | <0.001 | 65.23 (56.82, 73.65) | -15.01 (-24.17, -5.85) | 0.001 | 14.90 (2.33, 27.48) | 0.02 | 0.243 |
| 5 | 54.53 (46.17, 62.89) | -19.66 (-28.79, -10.52) | <0.001 | 50.50 (42.14, 58.85) | -29.74 (-38.84, -20.64) | <0.001 | -6.16 (-18.76, 6.45) | 0.339 | 0.060 |
| 6 | 46.35 (37.68, 55.02) | -27.84 (-37.24, -18.44) | <0.001 | 48.91 (40.11, 57.71) | -31.33 (-40.83, -21.83) | <0.001 | -3.13 (-16.28, 10.03) | 0.641 | 0.163 |
| 7 | 41.90 (32.04, 51.77) | -32.29 (-42.80, -21.78) | <0.001 | 47.03 (36.25, 57.80) | -33.21 (-44.63, -21.79) | <0.001 | 2.35 (-13.22, 17.92) | 0.768 | 0.588 |
| 1m | 55.86 (46.90, 64.82) | -18.33 (-28.08, -8.59) | <0.001 | 62.55 (54.50, 70.61) | -17.68 (-26.49, -8.87) | <0.001 | 3.54 (-9.41, 16.49) | 0.593 | 0.639 |
| 3m | 58.19 (48.97, 67.41) | -16.00 (-25.93, -6.07) | 0.002 | 73.78 (64.82, 82.74) | -6.46 (-16.14, 3.22) | 0.191 | 12.97 (-0.78, 26.73) | 0.064 | 0.410 |

Table 4: C30 Emotional functioning

|  | CMW (Tx) | | | SoC (Cx) | | |  |  |  |
| --- | --- | --- | --- | --- | --- | --- | --- | --- | --- |
| Time | Est mean (95%CI) | Mean change from baseline (95%CI) | p | Est mean (95%CI) | Mean change from baseline (95%CI) | p | Adjusted mean (95%CI) group difference | p# | P* |
| 0 | 75.48 (68.35, 82.62) |  |  | 75.90 (68.82, 82.98) |  |  | -2.69 (-13.78, 8.40) | 0.634 |  |
| 1 | 78.59 (71.76, 85.43) | 3.11 (-4.14, 10.36) | 0.401 | 75.95 (69.11, 82.80) | 0.05 (-7.11, 7.21) | 0.989 | -6.37 (-17.29, 4.55) | 0.253 | 0.516 |
| 2 | 74.89 (67.81, 81.97) | -0.59 (-8.04, 6.85) | 0.876 | 73.82 (67.09, 80.55) | -2.08 (-9.16, 5.00) | 0.564 | -1.48 (-12.57, 9.60) | 0.793 | 0.833 |
| 3 | 78.51 (71.75, 85.27) | 3.03 (-4.11, 10.16) | 0.406 | 72.28 (65.66, 78.90) | -3.62 (-10.59, 3.34) | 0.308 | -7.00 (-17.56, 3.56) | 0.194 | 0.431 |
| 4 | 69.56 (62.88, 76.25) | -5.92 (-13.01, 1.17) | 0.102 | 68.16 (61.27, 75.05) | -7.74 (-14.96, -0.53) | 0.035 | -1.94 (-12.67, 8.80) | 0.724 | 0.892 |
| 5 | 71.01 (64.16, 77.86) | -4.47 (-11.67, 2.73) | 0.223 | 64.26 (57.41, 71.11) | -11.64 (-18.82, -4.47) | 0.001 | -8.54 (-19.30, 2.21) | 0.120 | 0.294 |
| 6 | 69.62 (62.54, 76.71) | -5.86 (-13.27, 1.55) | 0.121 | 66.20 (59.01, 73.39) | -9.70 (-17.19, -2.22) | 0.011 | -3.40 (-14.57, 7.78) | 0.551 | 0.903 |
| 7 | 68.86 (60.86, 76.86) | -6.62 (-14.90, 1.66) | 0.117 | 63.60 (54.90, 72.30) | -12.30 (-21.30, -3.30) | 0.007 | -7.66 (-20.71, 5.39) | 0.25 | 0.459 |
| 1m | 73.34 (66.03, 80.65) | -2.14 (-9.82, 5.54) | 0.585 | 72.24 (65.62, 78.86) | -3.67 (-10.61, 3.27) | 0.301 | -2.77 (-13.79, 8.25) | 0.622 | 0.989 |
| 3m | 75.32 (67.81, 82.82) | -0.16 (-7.99, 7.66) | 0.967 | 78.45 (71.14, 85.75) | 2.54 (-5.08, 10.17) | 0.513 | 0.16 (-11.48, 11.80) | 0.978 | 0.635 |

Table 5: C30 Cognitive functioning

|  | CMW (Tx) | | | SoC (Cx) | | |  |  |  |
| --- | --- | --- | --- | --- | --- | --- | --- | --- | --- |
| Time | Est mean (95%CI) | Mean change from baseline (95%CI) | p | Est mean (95%CI) | Mean change from baseline (95%CI) | p | Adjusted mean (95%CI) group difference | p# | P* |
| 0 | 76.33 (69.12, 83.53) |  |  | 80.41 (73.27, 87.55) |  |  | 1.41 (-9.51, 12.33) | 0.8 |  |
| 1 | 75.17 (68.29, 82.05) | -1.15 (-8.74, 6.43) | 0.766 | 79.03 (72.14, 85.92) | -1.38 (-8.87, 6.11) | 0.718 | 1.14 (-9.59, 11.86) | 0.836 | 0.963 |
| 2 | 75.34 (68.20, 82.48) | -0.99 (-8.78, 6.80) | 0.803 | 76.41 (69.64, 83.17) | -4.01 (-11.41, 3.40) | 0.289 | 1.36 (-9.55, 12.27) | 0.807 | 0.993 |
| 3 | 77.14 (70.35, 83.93) | 0.81 (-6.65, 8.28) | 0.831 | 75.23 (68.59, 81.87) | -5.18 (-12.46, 2.10) | 0.163 | -1.50 (-11.83, 8.82) | 0.775 | 0.610 |
| 4 | 71.70 (64.99, 78.42) | -4.62 (-12.04, 2.79) | 0.222 | 73.58 (66.64, 80.52) | -6.83 (-14.38, 0.71) | 0.076 | 0.99 (-9.53, 11.52) | 0.853 | 0.943 |
| 5 | 71.43 (64.53, 78.32) | -4.90 (-12.43, 2.63) | 0.202 | 67.52 (60.63, 74.41) | -12.89 (-20.39, -5.39) | 0.001 | -3.87 (-14.42, 6.68) | 0.472 | 0.365 |
| 6 | 71.02 (63.87, 78.17) | -5.30 (-13.05, 2.44) | 0.180 | 67.10 (59.84, 74.36) | -13.31 (-21.14, -5.48) | 0.001 | -2.30 (-13.31, 8.72) | 0.683 | 0.539 |
| 7 | 67.63 (59.50, 75.76) | -8.70 (-17.36, -0.03) | 0.049 | 69.46 (60.58, 78.34) | -10.95 (-20.36, -1.54) | 0.023 | -1.91 (-14.97, 11.15) | 0.774 | 0.636 |
| 1m | 71.12 (63.73, 78.51) | -5.21 (-13.24, 2.83) | 0.204 | 71.42 (64.77, 78.06) | -9.00 (-16.26, -1.74) | 0.015 | -1.25 (-12.09, 9.59) | 0.821 | 0.655 |
| 3m | 75.77 (68.16, 83.37) | -0.56 (-8.74, 7.62) | 0.894 | 79.30 (71.92, 86.69) | -1.11 (-9.09, 6.86) | 0.785 | 0.53 (-10.99, 12.05) | 0.928 | 0.889 |

Table 6: C30 Social functioning

|  | CMW (Tx) | | | SoC (Cx) | | |  |  |  |
| --- | --- | --- | --- | --- | --- | --- | --- | --- | --- |
| Time | Est mean (95%CI) | Mean change from baseline (95%CI) | p | Est mean (95%CI) | Mean change from baseline (95%CI) | p | Adjusted mean (95%CI) group difference | p# | P* |
| 0 | 71.27 (62.36, 80.19) |  |  | 72.01 (63.17, 80.84) |  |  | 0.56 (-12.84, 13.95) | 0.935 |  |
| 1 | 72.46 (63.93, 80.98) | 1.18 (-8.13, 10.49) | 0.804 | 76.54 (68.01, 85.07) | 4.54 (-4.66, 13.74) | 0.334 | 2.66 (-10.50, 15.83) | 0.692 | 0.767 |
| 2 | 67.19 (58.35, 76.02) | -4.09 (-13.65, 5.47) | 0.402 | 68.41 (60.04, 76.79) | -3.59 (-12.68, 5.49) | 0.438 | 2.41 (-10.97, 15.79) | 0.724 | 0.797 |
| 3 | 66.94 (58.53, 75.36) | -4.33 (-13.50, 4.83) | 0.354 | 69.73 (61.50, 77.97) | -2.27 (-11.21, 6.67) | 0.618 | 3.15 (-9.55, 15.84) | 0.627 | 0.706 |
| 4 | 57.54 (49.22, 65.85) | -13.74 (-22.84, -4.64) | 0.003 | 60.39 (51.80, 68.99) | -11.61 (-20.88, -2.35) | 0.014 | 2.57 (-10.35, 15.50) | 0.696 | 0.773 |
| 5 | 62.25 (53.71, 70.78) | -9.03 (-18.27, 0.21) | 0.056 | 51.45 (42.92, 59.98) | -20.56 (-29.77, -11.35) | <0.001 | -10.37 (-23.33, 2.59) | 0.117 | 0.120 |
| 6 | 58.35 (49.50, 67.19) | -12.93 (-22.44, -3.41) | 0.008 | 52.19 (43.21, 61.18) | -19.81 (-29.42, -10.20) | <0.001 | -7.57 (-21.08, 5.94) | 0.272 | 0.264 |
| 7 | 53.78 (43.73, 63.83) | -17.50 (-28.13, -6.86) | 0.001 | 61.14 (50.18, 72.11) | -10.86 (-22.42, 0.69) | 0.065 | 6.12 (-9.82, 22.06) | 0.452 | 0.510 |
| 1m | 65.84 (56.70, 74.98) | -5.43 (-15.29, 4.43) | 0.280 | 58.87 (50.64, 67.10) | -13.14 (-22.05, -4.22) | 0.004 | -8.53 (-21.83, 4.77) | 0.209 | 0.206 |
| 3m | 66.08 (56.68, 75.48) | -5.20 (-15.24, 4.85) | 0.311 | 72.06 (62.93, 81.20) | 0.06 (-9.73, 9.85) | 0.991 | 3.77 (-10.34, 17.88) | 0.6 | 0.670 |

Table 7: C30 Fatigue

|  | CMW (Tx) | | | SoC (Cx) | | |  |  |  |
| --- | --- | --- | --- | --- | --- | --- | --- | --- | --- |
| Time | Est mean (95%CI) | Mean change from baseline (95%CI) | p | Est mean (95%CI) | Mean change from baseline (95%CI) | p | Adjusted mean (95%CI) group difference | p# | P* |
| 0 | 30.12 (22.67, 37.57) |  |  | 29.28 (21.90, 36.66) |  |  | -0.73 (-11.89, 10.43) | 0.898 |  |
| 1 | 40.16 (33.04, 47.27) | 10.04 (2.21, 17.87) | 0.012 | 32.76 (25.63, 39.88) | 3.48 (-4.26, 11.21) | 0.378 | -5.16 (-16.11, 5.79) | 0.356 | 0.472 |
| 2 | 41.13 (33.75, 48.52) | 11.02 (2.98, 19.05) | 0.007 | 34.61 (27.61, 41.60) | 5.33 (-2.31, 12.97) | 0.172 | -7.59 (-18.74, 3.56) | 0.182 | 0.271 |
| 3 | 40.96 (33.93, 47.99) | 10.84 (3.13, 18.55) | 0.006 | 38.08 (31.21, 44.95) | 8.80 (1.29, 16.32) | 0.022 | -2.03 (-12.56, 8.50) | 0.706 | 0.827 |
| 4 | 49.37 (42.43, 56.32) | 19.25 (11.60, 26.91) | <0.001 | 44.61 (37.43, 51.78) | 15.33 (7.54, 23.12) | <0.001 | -3.67 (-14.41, 7.07) | 0.503 | 0.628 |
| 5 | 49.18 (42.05, 56.31) | 19.06 (11.29, 26.83) | <0.001 | 58.41 (51.28, 65.53) | 29.13 (21.38, 36.87) | <0.001 | 11.89 (1.12, 22.65) | 0.03 | 0.038 |
| 6 | 56.18 (48.79, 63.57) | 26.06 (18.06, 34.06) | <0.001 | 56.71 (49.20, 64.21) | 27.43 (19.34, 35.51) | <0.001 | 3.72 (-7.54, 14.98) | 0.517 | 0.480 |
| 7 | 62.59 (54.18, 71.00) | 32.47 (23.53, 41.41) | <0.001 | 56.40 (47.22, 65.58) | 27.12 (17.40, 36.83) | <0.001 | -6.31 (-19.74, 7.12) | 0.357 | 0.445 |
| 1m | 47.95 (40.31, 55.59) | 17.84 (9.55, 26.13) | <0.001 | 44.99 (38.12, 51.86) | 15.71 (8.21, 23.20) | <0.001 | 1.23 (-9.85, 12.30) | 0.828 | 0.753 |
| 3m | 35.86 (27.99, 43.72) | 5.74 (-2.71, 14.18) | 0.183 | 35.14 (27.51, 42.78) | 5.87 (-2.37, 14.10) | 0.163 | 2.38 (-9.42, 14.18) | 0.693 | 0.635 |

Table 8: C30 Nausea & vomiting

|  | CMW (Tx) | | | SoC (Cx) | | |  |  |  |
| --- | --- | --- | --- | --- | --- | --- | --- | --- | --- |
| Time | Est mean (95%CI) | Mean change from baseline (95%CI) | p | Est mean (95%CI) | Mean change from baseline (95%CI) | p | Adjusted mean (95%CI) group difference | p# | P* |
| 0 | 5.06 (-1.69, 11.80) |  |  | 6.43 (-0.24, 13.10) |  |  | 3.27 (-7.15, 13.68) | 0.538 |  |
| 1 | 17.07 (10.70, 23.43) | 12.01 (3.93, 20.10) | 0.004 | 17.87 (11.51, 24.24) | 11.44 (3.44, 19.44) | 0.005 | 2.96 (-7.21, 13.12) | 0.568 | 0.962 |
| 2 | 13.38 (6.71, 20.06) | 8.33 (0.02, 16.63) | 0.049 | 16.20 (9.99, 22.41) | 9.77 (1.87, 17.67) | 0.015 | 0.75 (-9.65, 11.15) | 0.888 | 0.702 |
| 3 | 19.82 (13.56, 26.08) | 14.77 (6.80, 22.74) | <0.001 | 17.11 (11.05, 23.18) | 10.68 (2.91, 18.46) | 0.007 | -2.61 (-12.28, 7.05) | 0.596 | 0.351 |
| 4 | 29.08 (22.92, 35.24) | 24.02 (16.11, 31.93) | <0.001 | 24.89 (18.47, 31.32) | 18.46 (10.41, 26.52) | <0.001 | -2.01 (-11.93, 7.91) | 0.691 | 0.411 |
| 5 | 26.57 (20.20, 32.95) | 21.52 (13.47, 29.56) | <0.001 | 30.86 (24.50, 37.23) | 24.43 (16.43, 32.44) | <0.001 | 5.17 (-4.79, 15.12) | 0.309 | 0.768 |
| 6 | 29.12 (22.43, 35.80) | 24.06 (15.79, 32.34) | <0.001 | 38.95 (32.13, 45.76) | 32.52 (24.16, 40.88) | <0.001 | 9.30 (-1.24, 19.84) | 0.084 | 0.366 |
| 7 | 31.03 (23.18, 38.88) | 25.98 (16.74, 35.22) | <0.001 | 36.28 (27.56, 45.00) | 29.85 (19.83, 39.87) | <0.001 | 7.64 (-5.42, 20.69) | 0.252 | 0.572 |
| 1m | 14.46 (7.48, 21.43) | 9.40 (0.84, 17.97) | 0.031 | 19.39 (13.33, 25.46) | 12.96 (5.21, 20.72) | 0.001 | 7.38 (-2.94, 17.70) | 0.161 | 0.532 |
| 3m | 12.37 (5.14, 19.59) | 7.31 (-1.42, 16.04) | 0.101 | 8.11 (1.14, 15.07) | 1.68 (-6.83, 10.18) | 0.699 | -0.87 (-12.04, 10.30) | 0.878 | 0.549 |

Table 9: C30 Pain

|  | CMW (Tx) | | | SoC (Cx) | | |  |  |  |
| --- | --- | --- | --- | --- | --- | --- | --- | --- | --- |
| Time | Est mean (95%CI) | Mean change from baseline (95%CI) | p | Est mean (95%CI) | Mean change from baseline (95%CI) | p | Adjusted mean (95%CI) group difference | p# | P* |
| 0 | 25.02 (17.02, 33.01) |  |  | 23.52 (15.61, 31.43) |  |  | 3.22 (-9.01, 15.45) | 0.606 |  |
| 1 | 26.88 (19.30, 34.45) | 1.86 (-7.28, 11.01) | 0.690 | 16.98 (9.40, 24.55) | -6.54 (-15.59, 2.50) | 0.156 | -2.64 (-14.61, 9.33) | 0.666 | 0.414 |
| 2 | 29.65 (21.73, 37.56) | 4.63 (-4.76, 14.02) | 0.334 | 21.96 (14.55, 29.37) | -1.56 (-10.49, 7.37) | 0.732 | -4.32 (-16.54, 7.90) | 0.488 | 0.299 |
| 3 | 37.79 (30.33, 45.25) | 12.78 (3.77, 21.79) | 0.005 | 34.19 (26.93, 41.44) | 10.66 (1.87, 19.45) | 0.017 | -2.85 (-14.31, 8.60) | 0.625 | 0.382 |
| 4 | 41.05 (33.70, 48.41) | 16.04 (7.09, 24.98) | <0.001 | 36.47 (28.83, 44.11) | 12.95 (3.84, 22.06) | 0.005 | -5.44 (-17.16, 6.27) | 0.362 | 0.220 |
| 5 | 40.38 (32.79, 47.97) | 15.37 (6.28, 24.45) | 0.001 | 47.60 (40.02, 55.18) | 24.08 (15.03, 33.13) | <0.001 | 6.06 (-5.69, 17.80) | 0.312 | 0.689 |
| 6 | 51.21 (43.29, 59.13) | 26.19 (16.84, 35.55) | <0.001 | 50.21 (42.15, 58.27) | 26.69 (17.24, 36.14) | <0.001 | -1.45 (-13.81, 10.91) | 0.818 | 0.525 |
| 7 | 51.85 (42.65, 61.06) | 26.84 (16.39, 37.29) | <0.001 | 48.10 (37.95, 58.25) | 24.58 (13.24, 35.92) | <0.001 | -1.29 (-16.30, 13.72) | 0.866 | 0.597 |
| 1m | 27.53 (19.29, 35.76) | 2.51 (-7.17, 12.20) | 0.611 | 29.31 (22.06, 36.57) | 5.79 (-2.97, 14.56) | 0.195 | 3.44 (-8.69, 15.56) | 0.579 | 0.976 |
| 3m | 25.67 (17.15, 34.19) | 0.65 (-9.22, 10.53) | 0.897 | 13.61 (5.38, 21.83) | -9.92 (-19.53, -0.30) | 0.043 | -6.39 (-19.41, 6.64) | 0.337 | 0.208 |

Table 10: C30 Dyspnoea

|  | CMW (Tx) | | | SoC (Cx) | | |  |  |  |
| --- | --- | --- | --- | --- | --- | --- | --- | --- | --- |
| Time | Est mean (95%CI) | Mean change from baseline (95%CI) | p | Est mean (95%CI) | Mean change from baseline (95%CI) | p | Adjusted mean (95%CI) group difference | p# | P* |
| 0 | 11.69 (4.28, 19.09) |  |  | 13.98 (6.65, 21.32) |  |  | 0.61 (-10.87, 12.10) | 0.917 |  |
| 1 | 14.02 (6.99, 21.05) | 2.34 (-6.00, 10.67) | 0.583 | 14.42 (7.38, 21.45) | 0.43 (-7.81, 8.67) | 0.918 | 2.64 (-8.63, 13.90) | 0.646 | 0.754 |
| 2 | 17.71 (10.38, 25.05) | 6.03 (-2.53, 14.59) | 0.167 | 10.82 (3.93, 17.70) | -3.17 (-11.31, 4.97) | 0.445 | -6.31 (-17.79, 5.16) | 0.281 | 0.290 |
| 3 | 19.93 (13.00, 26.86) | 8.24 (0.03, 16.45) | 0.049 | 15.32 (8.57, 22.07) | 1.34 (-6.67, 9.35) | 0.744 | -2.95 (-13.76, 7.86) | 0.593 | 0.569 |
| 4 | 22.31 (15.48, 29.15) | 10.63 (2.48, 18.78) | 0.011 | 18.16 (11.06, 25.25) | 4.17 (-4.13, 12.47) | 0.324 | -5.48 (-16.51, 5.56) | 0.331 | 0.339 |
| 5 | 23.77 (16.72, 30.81) | 12.08 (3.80, 20.36) | 0.004 | 25.39 (18.36, 32.43) | 11.41 (3.16, 19.66) | 0.007 | -3.55 (-14.62, 7.51) | 0.529 | 0.514 |
| 6 | 25.62 (18.28, 32.97) | 13.94 (5.42, 22.46) | 0.001 | 19.27 (11.79, 26.74) | 5.28 (-3.33, 13.89) | 0.229 | -5.42 (-17.02, 6.17) | 0.359 | 0.361 |
| 7 | 24.78 (16.30, 33.27) | 13.10 (3.58, 22.62) | 0.007 | 21.84 (12.49, 31.19) | 7.85 (-2.49, 18.19) | 0.137 | -2.24 (-16.15, 11.67) | 0.753 | 0.711 |
| 1m | 24.66 (17.04, 32.29) | 12.98 (4.15, 21.81) | 0.004 | 19.33 (12.58, 26.08) | 5.35 (-2.64, 13.33) | 0.190 | -3.09 (-14.49, 8.30) | 0.595 | 0.571 |
| 3m | 21.36 (13.48, 29.23) | 9.67 (0.68, 18.66) | 0.035 | 9.90 (2.29, 17.52) | -4.08 (-12.84, 4.69) | 0.362 | -7.42 (-19.59, 4.75) | 0.232 | 0.242 |

Table 11: C30 Insomnia

|  | CMW (Tx) | | | SoC (Cx) | | |  |  |  |
| --- | --- | --- | --- | --- | --- | --- | --- | --- | --- |
| Time | Est mean (95%CI) | Mean change from baseline (95%CI) | p | Est mean (95%CI) | Mean change from baseline (95%CI) | p | Adjusted mean (95%CI) group difference | p# | P* |
| 0 | 28.81 (19.36, 38.25) |  |  | 35.83 (26.49, 45.18) |  |  | 4.70 (-9.81, 19.22) | 0.526 |  |
| 1 | 28.76 (19.81, 37.70) | -0.05 (-10.92, 10.82) | 0.993 | 24.74 (15.79, 33.68) | -11.09 (-21.84, -0.35) | 0.043 | -4.55 (-18.76, 9.66) | 0.530 | 0.274 |
| 2 | 37.77 (28.42, 47.11) | 8.96 (-2.20, 20.12) | 0.116 | 28.20 (19.45, 36.95) | -7.63 (-18.24, 2.98) | 0.159 | -4.04 (-18.54, 10.46) | 0.585 | 0.308 |
| 3 | 37.26 (28.45, 46.07) | 8.45 (-2.25, 19.16) | 0.122 | 25.45 (16.89, 34.01) | -10.38 (-20.83, 0.07) | 0.052 | -15.00 (-28.61, -1.40) | 0.031 | 0.016 |
| 4 | 42.56 (33.88, 51.24) | 13.76 (3.13, 24.39) | 0.011 | 31.15 (22.12, 40.18) | -4.68 (-15.51, 6.15) | 0.397 | -9.75 (-23.66, 4.15) | 0.169 | 0.083 |
| 5 | 40.71 (31.75, 49.67) | 11.91 (1.10, 22.71) | 0.031 | 43.99 (35.04, 52.94) | 8.16 (-2.60, 18.91) | 0.137 | -1.48 (-15.43, 12.47) | 0.835 | 0.460 |
| 6 | 44.79 (35.43, 54.15) | 15.98 (4.87, 27.10) | 0.005 | 44.73 (35.21, 54.26) | 8.90 (-2.33, 20.13) | 0.120 | 1.33 (-13.33, 16.00) | 0.859 | 0.697 |
| 7 | 47.34 (36.46, 58.22) | 18.53 (6.12, 30.95) | 0.003 | 37.36 (25.34, 49.38) | 1.53 (-11.95, 15.01) | 0.824 | -8.89 (-26.67, 8.89) | 0.327 | 0.176 |
| 1m | 28.61 (18.88, 38.35) | -0.19 (-11.70, 11.32) | 0.974 | 34.27 (25.71, 42.83) | -1.56 (-11.98, 8.86) | 0.770 | 2.85 (-11.54, 17.25) | 0.698 | 0.829 |
| 3m | 36.83 (26.76, 46.89) | 8.02 (-3.71, 19.75) | 0.180 | 30.21 (20.49, 39.93) | -5.62 (-17.05, 5.81) | 0.335 | -5.72 (-21.16, 9.72) | 0.468 | 0.246 |

Table 12: C30 Appetite loss

|  | CMW (Tx) | | | SoC (Cx) | | |  |  |  |
| --- | --- | --- | --- | --- | --- | --- | --- | --- | --- |
| Time | Est mean (95%CI) | Mean change from baseline (95%CI) | p | Est mean (95%CI) | Mean change from baseline (95%CI) | p | Adjusted mean (95%CI) group difference | p# | P* |
| 0 | 17.35 (7.86, 26.84) |  |  | 18.47 (9.09, 27.85) |  |  | 1.23 (-12.81, 15.28) | 0.863 |  |
| 1 | 31.22 (22.26, 40.18) | 13.87 (2.60, 25.14) | 0.016 | 25.15 (16.19, 34.10) | 6.67 (-4.48, 17.83) | 0.241 | -6.60 (-20.31, 7.12) | 0.346 | 0.367 |
| 2 | 37.46 (28.07, 46.85) | 20.11 (8.53, 31.69) | 0.001 | 39.23 (30.48, 47.97) | 20.75 (9.74, 31.77) | <0.001 | -1.76 (-15.79, 12.27) | 0.805 | 0.733 |
| 3 | 47.35 (38.54, 56.16) | 30.00 (18.89, 41.11) | <0.001 | 51.35 (42.81, 59.90) | 32.88 (22.04, 43.72) | <0.001 | -1.83 (-14.89, 11.23) | 0.783 | 0.716 |
| 4 | 66.35 (57.67, 75.02) | 49.00 (37.97, 60.03) | <0.001 | 65.24 (56.19, 74.28) | 46.77 (35.53, 58.00) | <0.001 | -7.36 (-20.75, 6.02) | 0.281 | 0.315 |
| 5 | 64.40 (55.43, 73.38) | 47.06 (35.85, 58.27) | <0.001 | 77.69 (68.73, 86.65) | 59.22 (48.05, 70.38) | <0.001 | 8.90 (-4.53, 22.33) | 0.194 | 0.371 |
| 6 | 78.93 (69.53, 88.32) | 61.58 (50.04, 73.11) | <0.001 | 79.69 (70.11, 89.26) | 61.21 (49.56, 72.87) | <0.001 | -1.26 (-15.47, 12.95) | 0.862 | 0.779 |
| 7 | 80.67 (69.65, 91.69) | 63.32 (50.44, 76.20) | <0.001 | 78.11 (65.89, 90.33) | 59.64 (45.66, 73.61) | <0.001 | -7.41 (-24.95, 10.14) | 0.408 | 0.402 |
| 1m | 48.36 (38.56, 58.15) | 31.01 (19.07, 42.95) | <0.001 | 53.78 (45.24, 62.32) | 35.31 (24.49, 46.12) | <0.001 | 6.43 (-7.48, 20.35) | 0.365 | 0.554 |
| 3m | 41.65 (31.50, 51.80) | 24.30 (12.13, 36.47) | <0.001 | 42.25 (32.46, 52.04) | 23.78 (11.92, 35.64) | <0.001 | 6.32 (-8.72, 21.36) | 0.410 | 0.246 |

Table 13: C30 Constipation

|  | CMW (Tx) | | | SoC (Cx) | | |  |  |  |
| --- | --- | --- | --- | --- | --- | --- | --- | --- | --- |
| Time | Est mean (95%CI) | Mean change from baseline (95%CI) | p | Est mean (95%CI) | Mean change from baseline (95%CI) | p | Adjusted mean (95%CI) group difference | p# | P* |
| 0 | 9.10 (0.02, 18.17) |  |  | 9.93 (0.96, 18.90) |  |  | -0.10 (-14.13, 13.93) | 0.989 |  |
| 1 | 24.89 (16.32, 33.45) | 15.79 (5.01, 26.56) | 0.004 | 25.60 (17.03, 34.16) | 15.67 (5.01, 26.33) | 0.004 | 3.57 (-10.13, 17.27) | 0.610 | 0.674 |
| 2 | 21.48 (12.50, 30.46) | 12.38 (1.31, 23.45) | 0.028 | 27.69 (19.32, 36.05) | 17.76 (7.23, 28.28) | 0.001 | 4.66 (-9.36, 18.68) | 0.515 | 0.590 |
| 3 | 24.92 (16.50, 33.35) | 15.83 (5.20, 26.45) | 0.003 | 23.97 (15.80, 32.14) | 14.04 (3.68, 24.41) | 0.008 | -3.15 (-16.19, 9.88) | 0.635 | 0.718 |
| 4 | 29.45 (21.16, 37.75) | 20.36 (9.82, 30.90) | <0.001 | 34.07 (25.42, 42.71) | 24.14 (13.40, 34.88) | <0.001 | 2.75 (-10.61, 16.12) | 0.686 | 0.740 |
| 5 | 34.35 (25.77, 42.93) | 25.25 (14.54, 35.96) | <0.001 | 38.97 (30.40, 47.54) | 29.04 (18.37, 39.71) | <0.001 | -1.27 (-14.68, 12.15) | 0.853 | 0.892 |
| 6 | 39.35 (30.36, 48.34) | 30.25 (19.23, 41.28) | <0.001 | 37.63 (28.48, 46.79) | 27.71 (16.57, 38.84) | <0.001 | -2.75 (-16.94, 11.45) | 0.704 | 0.767 |
| 7 | 34.82 (24.29, 45.36) | 25.73 (13.41, 38.04) | <0.001 | 50.78 (39.09, 62.46) | 40.85 (27.49, 54.21) | <0.001 | 18.76 (1.21, 36.32) | 0.036 | 0.069 |
| 1m | 19.26 (9.89, 28.62) | 10.16 (-1.26, 21.57) | 0.081 | 34.26 (26.09, 42.43) | 24.33 (14.00, 34.67) | <0.001 | 16.93 (3.03, 30.83) | 0.017 | 0.054 |
| 3m | 14.33 (4.62, 24.03) | 5.23 (-6.40, 16.86) | 0.378 | 17.60 (8.25, 26.96) | 7.68 (-3.66, 19.01) | 0.184 | 5.86 (-9.18, 20.90) | 0.445 | 0.520 |

Table 14: C30 Diarrhoea

|  | CMW (Tx) | | | SoC (Cx) | | |  |  |  |
| --- | --- | --- | --- | --- | --- | --- | --- | --- | --- |
| Time | Est mean (95%CI) | Mean change from baseline (95%CI) | p | Est mean (95%CI) | Mean change from baseline (95%CI) | p | Adjusted mean (95%CI) group difference | p# | P* |
| 0 | 7.07 (0.49, 13.65) |  |  | 13.26 (6.78, 19.75) |  |  | 8.20 (-2.06, 18.47) | 0.117 |  |
| 1 | 6.88 (0.73, 13.02) | -0.19 (-8.72, 8.34) | 0.966 | 6.53 (0.39, 12.67) | -6.73 (-15.19, 1.73) | 0.119 | 2.65 (-7.35, 12.65) | 0.604 | 0.424 |
| 2 | 6.75 (0.26, 13.24) | -0.32 (-9.09, 8.45) | 0.944 | 5.08 (-0.88, 11.04) | -8.19 (-16.53, 0.16) | 0.054 | -1.42 (-11.68, 8.83) | 0.786 | 0.172 |
| 3 | 7.98 (1.96, 14.00) | 0.91 (-7.52, 9.34) | 0.833 | 5.78 (-0.02, 11.58) | -7.48 (-15.71, 0.74) | 0.074 | -3.40 (-12.82, 6.02) | 0.480 | 0.086 |
| 4 | 10.40 (4.49, 16.30) | 3.33 (-5.04, 11.70) | 0.435 | 8.12 (1.92, 14.33) | -5.14 (-13.65, 3.37) | 0.237 | -0.27 (-9.98, 9.44) | 0.956 | 0.217 |
| 5 | 9.58 (3.44, 15.73) | 2.52 (-6.00, 11.03) | 0.563 | 10.60 (4.45, 16.74) | -2.67 (-11.13, 5.79) | 0.537 | 2.16 (-7.58, 11.91) | 0.663 | 0.380 |
| 6 | 15.68 (9.19, 22.17) | 8.61 (-0.14, 17.37) | 0.054 | 14.06 (7.42, 20.70) | 0.80 (-8.03, 9.62) | 0.860 | -4.56 (-14.97, 5.86) | 0.391 | 0.074 |
| 7 | 19.95 (12.16, 27.75) | 12.89 (3.14, 22.63) | 0.010 | 9.25 (0.50, 18.00) | -4.01 (-14.56, 6.53) | 0.455 | -9.06 (-22.31, 4.18) | 0.180 | 0.036 |
| 1m | 7.22 (0.40, 14.05) | 0.16 (-8.87, 9.19) | 0.973 | 13.77 (7.97, 19.57) | 0.51 (-7.71, 8.72) | 0.904 | 8.09 (-2.06, 18.25) | 0.118 | 0.988 |
| 3m | 9.78 (2.68, 16.88) | 2.71 (-6.50, 11.92) | 0.564 | 4.11 (-2.71, 10.93) | -9.16 (-18.14, -0.18) | 0.046 | -5.12 (-16.26, 6.02) | 0.368 | 0.071 |

Table 15: C30 Financial difficulties

|  | CMW (Tx) | | | SoC (Cx) | | |  |  |  |
| --- | --- | --- | --- | --- | --- | --- | --- | --- | --- |
| Time | Est mean (95%CI) | Mean change from baseline (95%CI) | p | Est mean (95%CI) | Mean change from baseline (95%CI) | p | Adjusted mean (95%CI) group difference | p# | P* |
| 0 | 35.42 (25.57, 45.27) |  |  | 29.37 (19.59, 39.14) |  |  | -3.41 (-17.98, 11.17) | 0.647 |  |
| 1 | 33.73 (24.28, 43.18) | -1.69 (-11.55, 8.17) | 0.737 | 16.56 (7.10, 26.02) | -12.80 (-22.54, -3.07) | 0.010 | -13.89 (-28.22, 0.44) | 0.057 | 0.173 |
| 2 | 33.56 (23.79, 43.33) | -1.86 (-11.98, 8.26) | 0.718 | 29.74 (20.43, 39.04) | 0.37 (-9.25, 9.99) | 0.940 | -7.00 (-21.57, 7.56) | 0.346 | 0.644 |
| 3 | 30.22 (20.88, 39.56) | -5.20 (-14.90, 4.50) | 0.294 | 24.53 (15.37, 33.69) | -4.83 (-14.30, 4.63) | 0.317 | -3.63 (-17.45, 10.19) | 0.607 | 0.976 |
| 4 | 40.22 (30.98, 49.46) | 4.80 (-4.84, 14.43) | 0.329 | 31.16 (21.64, 40.69) | 1.80 (-8.01, 11.61) | 0.719 | -10.79 (-24.86, 3.28) | 0.133 | 0.330 |
| 5 | 36.31 (26.85, 45.78) | 0.89 (-8.89, 10.67) | 0.858 | 35.99 (26.52, 45.45) | 6.62 (-3.13, 16.37) | 0.183 | 3.60 (-10.51, 17.70) | 0.617 | 0.356 |
| 6 | 40.10 (30.33, 49.88) | 4.68 (-5.39, 14.75) | 0.362 | 42.09 (32.17, 52.01) | 12.72 (2.55, 22.90) | 0.014 | 4.61 (-10.08, 19.31) | 0.538 | 0.308 |
| 7 | 45.38 (34.37, 56.38) | 9.95 (-1.30, 21.21) | 0.083 | 34.01 (22.06, 45.96) | 4.64 (-7.59, 16.88) | 0.457 | -7.51 (-24.82, 9.81) | 0.396 | 0.654 |
| 1m | 35.80 (25.72, 45.87) | 0.38 (-10.06, 10.81) | 0.944 | 30.89 (21.73, 40.06) | 1.53 (-7.90, 10.96) | 0.751 | -4.13 (-18.61, 10.35) | 0.576 | 0.926 |
| 3m | 37.08 (26.73, 47.42) | 1.66 (-8.98, 12.29) | 0.760 | 31.32 (21.25, 41.40) | 1.96 (-8.40, 12.32) | 0.711 | -7.31 (-22.66, 8.03) | 0.350 | 0.633 |

**EORTC QLQ-C30 10-point change**

Table 1a: C30 item score change categories from baseline

|  |  | QoL global scale | | | Physical functioning | | | Role functioning | | |
| --- | --- | --- | --- | --- | --- | --- | --- | --- | --- | --- |
|  | Score change | CMW (Tx) | SoC (Cx) |  | CMW (Tx) | SoC (Cx) |  | CMW (Tx) | SoC (Cx) |  |
|  |  | N (%) | N (%) | p | N (%) | N (%) | p | N (%) | N (%) | p |
| 1w | ≥10 decrease | 10 (33.3%) | 8 (25.0%) | 0.604 | 8 (26.7%) | 7 (21.9%) | 0.890 | 11 (36.7%) | 15 (46.9%) | 0.606 |
|  | nil | 16 (53.3%) | 17 (53.1%) |  | 18 (60.0%) | 21 (65.6%) |  | 14 (46.7%) | 11 (34.4%) |  |
|  | ≥10 increase | 4 (13.3%) | 7 (21.9%) |  | 4 (13.3%) | 4 (12.5%) |  | 5 (16.7%) | 6 (18.8%) |  |
| 2w | ≥10 decrease | 7 (25.0%) | 12 (37.5%) | 0.278 | 4 (14.3%) | 7 (21.9%) | 0.706 | 11 (39.3%) | 12 (37.5%) | 0.575 |
|  | nil | 18 (64.3%) | 14 (43.8%) |  | 21 (75.0%) | 21 (65.6%) |  | 11 (39.3%) | 16 (50.0%) |  |
|  | ≥10 increase | 3 (10.7%) | 6 (18.8%) |  | 3 (10.7%) | 4 (12.5%) |  | 6 (21.4%) | 4 (12.5%) |  |
| 3w | ≥10 decrease | 16 (48.5%) | 19 (55.9%) | 0.494 | 8 (24.2%) | 12 (35.3%) | 0.583 | 11 (33.3%) | 15 (44.1%) | 0.542 |
|  | nil | 14 (42.4%) | 10 (29.4%) |  | 22 (66.7%) | 20 (58.8%) |  | 17 (51.5%) | 13 (38.2%) |  |
|  | ≥10 increase | 3 (9.1%) | 5 (14.7%) |  | 3 (9.1%) | 2 (5.9%) |  | 5 (15.2%) | 6 (17.6%) |  |
| 4w | ≥10 decrease | 16 (48.5%) | 17 (56.7%) | 0.580 | 13 (39.4%) | 12 (40.0%) | 0.878 | 24 (72.7%) | 16 (53.3%) | 0.278 |
|  | nil | 15 (45.5%) | 10 (33.3%) |  | 18 (54.5%) | 17 (56.7%) |  | 6 (18.2%) | 9 (30.0%) |  |
|  | ≥10 increase | 2 (6.1%) | 3 (10.0%) |  | 2 (6.1%) | 1 (3.3%) |  | 3 (9.1%) | 5 (16.7%) |  |
| 5w | ≥10 decrease | 21 (63.6%) | 21 (65.6%) | 0.611 | 14 (42.4%) | 18 (56.3%) | 0.509 | 18 (54.5%) | 21 (65.6%) | 0.602 |
|  | nil | 11 (33.3%) | 11 (34.4%) |  | 17 (51.5%) | 13 (40.6%) |  | 12 (36.4%) | 8 (25.0%) |  |
|  | ≥10 increase | 1 (3.0%) | 0 (0.0%) |  | 2 (6.1%) | 1 (3.1%) |  | 3 (9.1%) | 3 (9.4%) |  |
| 6w | ≥10 decrease | 18 (60.0%) | 15 (55.6%) | 0.316 | 17 (56.7%) | 14 (51.9%) | 0.716 | 22 (73.3%) | 17 (63.0%) | 0.683 |
|  | nil | 12 (40.0%) | 10 (37.0%) |  | 13 (43.3%) | 13 (48.1%) |  | 6 (20.0%) | 7 (25.9%) |  |
|  | ≥10 increase | 0 (0.0%) | 2 (7.4%) |  | 0 (0.0%) | 0 (0.0%) |  | 2 (6.7%) | 3 (11.1%) |  |
| 7w | ≥10 decrease | 15 (71.4%) | 10 (76.9%) | 0.326 | 14 (66.7%) | 7 (53.8%) | 0.260 | 30 (100.0%) | 27 (100.0%) | 0.914 |
|  | nil | 6 (28.6%) | 2 (15.4%) |  | 5 (23.8%) | 6 (46.2%) |  | 16 (76.2%) | 10 (76.9%) |  |
|  | ≥10 increase | 0 (0.0%) | 1 (7.7%) |  | 2 (9.5%) | 0 (0.0%) |  | 4 (19.0%) | 2 (15.4%) |  |
| 1m | ≥10 decrease | 11 (47.8%) | 15 (41.7%) | 0.674 | 13 (56.5%) | 15 (41.7%) | 0.205 | 21 (100.0%) | 13 (100.0%) | 0.905 |
|  | nil | 10 (43.5%) | 15 (41.7%) |  | 7 (30.4%) | 19 (52.8%) |  | 12 (52.2%) | 19 (52.8%) |  |
|  | ≥10 increase | 2 (8.7%) | 6 (16.7%) |  | 3 (13.0%) | 2 (5.6%) |  | 8 (34.8%) | 11 (30.6%) |  |
| 3m | ≥10 decrease | 11 (44.0%) | 5 (20.8%) | 0.213 | 11 (44.0%) | 7 (29.2%) | 0.431 | 23 (100.0%) | 36 (100.0%) | 0.178 |
|  | nil | 11 (44.0%) | 14 (58.3%) |  | 11 (44.0%) | 15 (62.5%) |  | 11 (44.0%) | 6 (25.0%) |  |
|  | ≥10 increase | 3 (12.0%) | 5 (20.8%) |  | 3 (12.0%) | 2 (8.3%) |  | 9 (36.0%) | 15 (62.5%) |  |

|  |  | Emotional functioning | | | Cognitive functioning | | | Social functioning | | |
| --- | --- | --- | --- | --- | --- | --- | --- | --- | --- | --- |
|  | Score change | CMW (Tx) | SoC (Cx) |  | CMW (Tx) | SoC (Cx) |  | CMW (Tx) | SoC (Cx) |  |
|  |  | N (%) | N (%) | p | N (%) | N (%) | p | N (%) | N (%) | p |
| 1w | ≥10 decrease | 9 (30.0%) | 8 (25.0%) | 0.575 | 9 (30.0%) | 11 (34.4%) | 0.934 | 5 (16.7%) | 8 (25.0%) | 0.664 |
|  | nil | 12 (40.0%) | 17 (53.1%) |  | 13 (43.3%) | 13 (40.6%) |  | 15 (50.0%) | 13 (40.6%) |  |
|  | ≥10 increase | 9 (30.0%) | 7 (21.9%) |  | 8 (26.7%) | 8 (25.0%) |  | 10 (33.3%) | 11 (34.4%) |  |
| 2w | ≥10 decrease | 7 (25.0%) | 8 (25.0%) | 0.956 | 9 (32.1%) | 11 (34.4%) | 0.642 | 8 (28.6%) | 11 (34.4%) | 0.885 |
|  | nil | 14 (50.0%) | 17 (53.1%) |  | 9 (32.1%) | 13 (40.6%) |  | 13 (46.4%) | 14 (43.8%) |  |
|  | ≥10 increase | 7 (25.0%) | 7 (21.9%) |  | 10 (35.7%) | 8 (25.0%) |  | 7 (25.0%) | 7 (21.9%) |  |
| 3w | ≥10 decrease | 3 (9.1%) | 11 (32.4%) | 0.020 | 9 (27.3%) | 14 (41.2%) | 0.452 | 7 (21.2%) | 14 (41.2%) | 0.095 |
|  | nil | 19 (57.6%) | 19 (55.9%) |  | 13 (39.4%) | 12 (35.3%) |  | 19 (57.6%) | 11 (32.4%) |  |
|  | ≥10 increase | 11 (33.3%) | 4 (11.8%) |  | 11 (33.3%) | 8 (23.5%) |  | 7 (21.2%) | 9 (26.5%) |  |
| 4w | ≥10 decrease | 12 (36.4%) | 9 (30.0%) | 0.340 | 15 (45.5%) | 10 (33.3%) | 0.51 | 18 (54.5%) | 12 (40.0%) | 0.365 |
|  | nil | 13 (39.4%) | 17 (56.7%) |  | 9 (27.3%) | 12 (40.0%) |  | 7 (21.2%) | 11 (36.7%) |  |
|  | ≥10 increase | 8 (24.2%) | 4 (13.3%) |  | 9 (27.3%) | 8 (26.7%) |  | 8 (24.2%) | 7 (23.3%) |  |
| 5w | ≥10 decrease | 10 (30.3%) | 9 (28.1%) | 0.982 | 12 (36.4%) | 15 (46.9%) | 0.678 | 13 (39.4%) | 20 (62.5%) | 0.110 |
|  | nil | 18 (54.5%) | 18 (56.3%) |  | 13 (39.4%) | 10 (31.3%) |  | 12 (36.4%) | 5 (15.6%) |  |
|  | ≥10 increase | 5 (15.2%) | 5 (15.6%) |  | 8 (24.2%) | 7 (21.9%) |  | 8 (24.2%) | 7 (21.9%) |  |
| 6w | ≥10 decrease | 11 (36.7%) | 5 (18.5%) | 0.314 | 11 (36.7%) | 10 (37.0%) | 0.731 | 12 (40.0%) | 16 (59.3%) | 0.285 |
|  | nil | 13 (43.3%) | 15 (55.6%) |  | 11 (36.7%) | 12 (44.4%) |  | 12 (40.0%) | 6 (22.2%) |  |
|  | ≥10 increase | 6 (20.0%) | 7 (25.9%) |  | 8 (26.7%) | 5 (18.5%) |  | 6 (20.0%) | 5 (18.5%) |  |
| 7w | ≥10 decrease | 9 (42.9%) | 2 (15.4%) | 0.199 | 11 (52.4%) | 4 (30.8%) | 0.367 | 9 (42.9%) | 6 (46.2%) | 0.816 |
|  | nil | 6 (28.6%) | 7 (53.8%) |  | 4 (19.0%) | 5 (38.5%) |  | 10 (47.6%) | 5 (38.5%) |  |
|  | ≥10 increase | 6 (28.6%) | 4 (30.8%) |  | 6 (28.6%) | 4 (30.8%) |  | 2 (9.5%) | 2 (15.4%) |  |
| 1m | ≥10 decrease | 5 (21.7%) | 5 (13.9%) | 0.633 | 8 (34.8%) | 10 (27.8%) | 0.802 | 9 (39.1%) | 15 (41.7%) | 0.414 |
|  | nil | 13 (56.5%) | 20 (55.6%) |  | 9 (39.1%) | 17 (47.2%) |  | 11 (47.8%) | 12 (33.3%) |  |
|  | ≥10 increase | 5 (21.7%) | 11 (30.6%) |  | 6 (26.1%) | 9 (25.0%) |  | 3 (13.0%) | 9 (25.0%) |  |
| 3m | ≥10 decrease | 5 (20.0%) | 5 (20.8%) | 0.839 | 7 (28.0%) | 9 (37.5%) | 0.778 | 7 (28.0%) | 9 (37.5%) | 0.261 |
|  | nil | 13 (52.0%) | 14 (58.3%) |  | 12 (48.0%) | 10 (41.7%) |  | 14 (56.0%) | 8 (33.3%) |  |
|  | ≥10 increase | 7 (28.0%) | 5 (20.8%) |  | 6 (24.0%) | 5 (20.8%) |  | 4 (16.0%) | 7 (29.2%) |  |

|  |  | Fatigue | | | Nausea & vomiting | | | Pain | | |
| --- | --- | --- | --- | --- | --- | --- | --- | --- | --- | --- |
|  | Score change | CMW (Tx) | SoC (Cx) |  | CMW (Tx) | SoC (Cx) |  | CMW (Tx) | SoC (Cx) |  |
|  |  | N (%) | N (%) | p | N (%) | N (%) | p | N (%) | N (%) | p |
| 1w | ≥10 decrease | 6 (20.0%) | 8 (25.0%) | 0.895 | 4 (13.3%) | 4 (12.5%) | 0.970 | 8 (26.7%) | 7 (21.9%) | 0.275 |
|  | nil | 11 (36.7%) | 11 (34.4%) |  | 11 (36.7%) | 11 (34.4%) |  | 11 (36.7%) | 18 (56.3%) |  |
|  | ≥10 increase | 13 (43.3%) | 13 (40.6%) |  | 15 (50.0%) | 17 (53.1%) |  | 11 (36.7%) | 7 (21.9%) |  |
| 2w | ≥10 decrease | 5 (17.9%) | 9 (28.1%) | 0.282 | 3 (10.7%) | 2 (6.3%) | 0.599 | 9 (32.1%) | 8 (25.0%) | 0.805 |
|  | nil | 5 (17.9%) | 9 (28.1%) |  | 16 (57.1%) | 16 (50.0%) |  | 8 (28.6%) | 11 (34.4%) |  |
|  | ≥10 increase | 18 (64.3%) | 14 (43.8%) |  | 9 (32.1%) | 14 (43.8%) |  | 11 (39.3%) | 13 (40.6%) |  |
| 3w | ≥10 decrease | 7 (21.2%) | 8 (23.5%) | 0.930 | 2 (6.1%) | 3 (8.8%) | 0.679 | 6 (18.2%) | 6 (17.6%) | 0.995 |
|  | nil | 8 (24.2%) | 7 (20.6%) |  | 12 (36.4%) | 15 (44.1%) |  | 8 (24.2%) | 8 (23.5%) |  |
|  | ≥10 increase | 18 (54.5%) | 19 (55.9%) |  | 19 (57.6%) | 16 (47.1%) |  | 19 (57.6%) | 20 (58.8%) |  |
| 4w | ≥10 decrease | 6 (18.2%) | 5 (16.7%) | 0.880 | 1 (3.0%) | 3 (10.0%) | 0.522 | 7 (21.2%) | 7 (23.3%) | 0.616 |
|  | nil | 6 (18.2%) | 7 (23.3%) |  | 9 (27.3%) | 8 (26.7%) |  | 3 (9.1%) | 5 (16.7%) |  |
|  | ≥10 increase | 21 (63.6%) | 18 (60.0%) |  | 23 (69.7%) | 19 (63.3%) |  | 23 (69.7%) | 18 (60.0%) |  |
| 5w | ≥10 decrease | 7 (21.2%) | 3 (9.4%) | 0.301 | 1 (3.0%) | 2 (6.3%) | 0.746 | 5 (15.2%) | 6 (18.8%) | 0.909 |
|  | nil | 4 (12.1%) | 7 (21.9%) |  | 10 (30.3%) | 11 (34.4%) |  | 6 (18.2%) | 5 (15.6%) |  |
|  | ≥10 increase | 22 (66.7%) | 22 (68.8%) |  | 22 (66.7%) | 19 (59.4%) |  | 22 (66.7%) | 21 (65.6%) |  |
| 6w | ≥10 decrease | 2 (6.7%) | 2 (7.4%) | 0.975 | 1 (3.3%) | 1 (3.7%) | 0.851 | 2 (6.7%) | 5 (18.5%) | 0.139 |
|  | nil | 5 (16.7%) | 5 (18.5%) |  | 8 (26.7%) | 9 (33.3%) |  | 7 (23.3%) | 2 (7.4%) |  |
|  | ≥10 increase | 23 (76.7%) | 20 (74.1%) |  | 21 (70.0%) | 17 (63.0%) |  | 21 (70.0%) | 20 (74.1%) |  |
| 7w | ≥10 decrease | 2 (9.5%) | 2 (15.4%) | 0.863 | 0 (0.0%) | 0 (0.0%) | 0.704 | 3 (14.3%) | 6 (46.2%) | 0.044 |
|  | nil | 3 (14.3%) | 2 (15.4%) |  | 5 (23.8%) | 4 (30.8%) |  | 5 (23.8%) | 0 (0.0%) |  |
|  | ≥10 increase | 16 (76.2%) | 9 (69.2%) |  | 16 (76.2%) | 9 (69.2%) |  | 13 (61.9%) | 7 (53.8%) |  |
| 1m | ≥10 decrease | 2 (8.7%) | 7 (19.4%) | 0.279 | 3 (13.0%) | 4 (11.1%) | 0.145 | 8 (34.8%) | 12 (33.3%) | 0.263 |
|  | nil | 9 (39.1%) | 8 (22.2%) |  | 16 (69.6%) | 17 (47.2%) |  | 9 (39.1%) | 8 (22.2%) |  |
|  | ≥10 increase | 12 (52.2%) | 21 (58.3%) |  | 4 (17.4%) | 15 (41.7%) |  | 6 (26.1%) | 16 (44.4%) |  |
| 3m | ≥10 decrease | 5 (20.0%) | 7 (29.2%) | 0.725 | 2 (8.0%) | 3 (12.5%) | 0.840 | 8 (32.0%) | 9 (37.5%) | 0.896 |
|  | nil | 8 (32.0%) | 6 (25.0%) |  | 19 (76.0%) | 18 (75.0%) |  | 12 (48.0%) | 10 (41.7%) |  |
|  | ≥10 increase | 12 (48.0%) | 11 (45.8%) |  | 4 (16.0%) | 3 (12.5%) |  | 5 (20.0%) | 5 (20.8%) |  |

|  |  | Dyspnoea | | | Insomnia | | | Appetite loss | | |
| --- | --- | --- | --- | --- | --- | --- | --- | --- | --- | --- |
|  | Score change | CMW (Tx) | SoC (Cx) |  | CMW (Tx) | SoC (Cx) |  | CMW (Tx) | SoC (Cx) |  |
|  |  | N (%) | N (%) | p | N (%) | N (%) | p | N (%) | N (%) | p |
| 1w | ≥10 decrease | 2 (6.7%) | 2 (6.3%) | 0.891 | 5 (16.7%) | 11 (34.4%) | 0.268 | 4 (13.3%) | 5 (15.6%) | 0.896 |
|  | nil | 23 (76.7%) | 26 (81.3%) |  | 20 (66.7%) | 16 (50.0%) |  | 15 (50.0%) | 17 (53.1%) |  |
|  | ≥10 increase | 5 (16.7%) | 4 (12.5%) |  | 5 (16.7%) | 5 (15.6%) |  | 11 (36.7%) | 10 (31.3%) |  |
| 2w | ≥10 decrease | 2 (7.1%) | 3 (9.4%) | 0.224 | 7 (25.0%) | 10 (31.3%) | 0.088 | 2 (7.1%) | 2 (6.3%) | 0.969 |
|  | nil | 20 (71.4%) | 27 (84.4%) |  | 12 (42.9%) | 19 (59.4%) |  | 14 (50.0%) | 17 (53.1%) |  |
|  | ≥10 increase | 6 (21.4%) | 2 (6.3%) |  | 9 (32.1%) | 3 (9.4%) |  | 12 (42.9%) | 13 (40.6%) |  |
| 3w | ≥10 decrease | 2 (6.1%) | 3 (8.8%) | 0.588 | 7 (21.2%) | 12 (35.3%) | 0.316 | 3 (9.1%) | 2 (5.9%) | 0.530 |
|  | nil | 26 (78.8%) | 23 (67.6%) |  | 16 (48.5%) | 16 (47.1%) |  | 10 (30.3%) | 7 (20.6%) |  |
|  | ≥10 increase | 5 (15.2%) | 8 (23.5%) |  | 10 (30.3%) | 6 (17.6%) |  | 20 (60.6%) | 25 (73.5%) |  |
| 4w | ≥10 decrease | 4 (12.1%) | 2 (6.7%) | 0.097 | 6 (18.2%) | 10 (33.3%) | 0.332 | 2 (6.1%) | 1 (3.3%) | 0.873 |
|  | nil | 18 (54.5%) | 24 (80.0%) |  | 14 (42.4%) | 12 (40.0%) |  | 5 (15.2%) | 5 (16.7%) |  |
|  | ≥10 increase | 11 (33.3%) | 4 (13.3%) |  | 13 (39.4%) | 8 (26.7%) |  | 26 (78.8%) | 24 (80.0%) |  |
| 5w | ≥10 decrease | 2 (6.1%) | 3 (9.4%) | 0.423 | 6 (18.2%) | 8 (25.0%) | 0.629 | 2 (6.1%) | 1 (3.1%) | 0.271 |
|  | nil | 19 (57.6%) | 22 (68.8%) |  | 12 (36.4%) | 13 (40.6%) |  | 6 (18.2%) | 2 (6.3%) |  |
|  | ≥10 increase | 12 (36.4%) | 7 (21.9%) |  | 15 (45.5%) | 11 (34.4%) |  | 25 (75.8%) | 29 (90.6%) |  |
| 6w | ≥10 decrease | 3 (10.0%) | 4 (14.8%) | 0.307 | 4 (13.3%) | 6 (22.2%) | 0.471 | 5 (16.7%) | 0 (0.0%) | 0.053 |
|  | nil | 16 (53.3%) | 18 (66.7%) |  | 8 (26.7%) | 9 (33.3%) |  | 25 (83.3%) | 27 (100.0%) |  |
|  | ≥10 increase | 11 (36.7%) | 5 (18.5%) |  | 18 (60.0%) | 12 (44.4%) |  | 0 (0.0%) | 0 (0.0%) |  |
| 7w | ≥10 decrease | 1 (4.8%) | 2 (15.4%) | 0.443 | 3 (14.3%) | 5 (38.5%) | 0.106 | 0 (0.0%) | 1 (7.7%) | 0.384 |
|  | nil | 14 (66.7%) | 9 (69.2%) |  | 8 (38.1%) | 6 (46.2%) |  | 3 (14.3%) | 1 (7.7%) |  |
|  | ≥10 increase | 6 (28.6%) | 2 (15.4%) |  | 10 (47.6%) | 2 (15.4%) |  | 18 (85.7%) | 11 (84.6%) |  |
| 1m | ≥10 decrease | 2 (8.7%) | 5 (13.9%) | 0.701 | 5 (21.7%) | 12 (33.3%) | 0.544 | 2 (8.7%) | 3 (8.3%) | 0.937 |
|  | nil | 14 (60.9%) | 23 (63.9%) |  | 9 (39.1%) | 10 (27.8%) |  | 8 (34.8%) | 11 (30.6%) |  |
|  | ≥10 increase | 7 (30.4%) | 8 (22.2%) |  | 9 (39.1%) | 14 (38.9%) |  | 13 (56.5%) | 22 (61.1%) |  |
| 3m | ≥10 decrease | 3 (12.0%) | 3 (12.5%) | 0.114 | 5 (20.0%) | 7 (29.2%) | 0.279 | 1 (4.0%) | 2 (8.3%) | 0.688 |
|  | nil | 14 (56.0%) | 19 (79.2%) |  | 14 (56.0%) | 8 (33.3%) |  | 13 (52.0%) | 10 (41.7%) |  |
|  | ≥10 increase | 8 (32.0%) | 2 (8.3%) |  | 6 (24.0%) | 9 (37.5%) |  | 11 (44.0%) | 12 (50.0%) |  |

|  |  | Constipation | | | Diarrhoea | | | Financial difficulties | | |
| --- | --- | --- | --- | --- | --- | --- | --- | --- | --- | --- |
|  | Score change | CMW (Tx) | SoC (Cx) |  | CMW (Tx) | SoC (Cx) |  | CMW (Tx) | SoC (Cx) |  |
|  |  | N (%) | N (%) | p | N (%) | N (%) | p | N (%) | N (%) | p |
| 1w | ≥10 decrease | 1 (3.3%) | 2 (6.3%) | 0.793 | 3 (10.0%) | 8 (25.0%) | 0.298 | 5 (16.7%) | 12 (37.5%) | 0.055 |
|  | nil | 18 (60.0%) | 17 (53.1%) |  | 23 (76.7%) | 20 (62.5%) |  | 23 (76.7%) | 15 (46.9%) |  |
|  | ≥10 increase | 11 (36.7%) | 13 (40.6%) |  | 4 (13.3%) | 4 (12.5%) |  | 2 (6.7%) | 5 (15.6%) |  |
| 2w | ≥10 decrease | 2 (7.1%) | 1 (3.1%) | 0.774 | 2 (7.1%) | 5 (15.6%) | 0.593 | 6 (21.4%) | 7 (21.9%) | 0.259 |
|  | nil | 17 (60.7%) | 20 (62.5%) |  | 23 (82.1%) | 24 (75.0%) |  | 20 (71.4%) | 18 (56.3%) |  |
|  | ≥10 increase | 9 (32.1%) | 11 (34.4%) |  | 3 (10.7%) | 3 (9.4%) |  | 2 (7.1%) | 7 (21.9%) |  |
| 3w | ≥10 decrease | 2 (6.1%) | 2 (5.9%) | 0.909 | 2 (6.1%) | 6 (17.6%) | 0.332 | 8 (24.2%) | 7 (20.6%) | 0.246 |
|  | nil | 21 (63.6%) | 20 (58.8%) |  | 27 (81.8%) | 25 (73.5%) |  | 24 (72.7%) | 22 (64.7%) |  |
|  | ≥10 increase | 10 (30.3%) | 12 (35.3%) |  | 4 (12.1%) | 3 (8.8%) |  | 1 (3.0%) | 5 (14.7%) |  |
| 4w | ≥10 decrease | 2 (6.1%) | 3 (10.0%) | 0.714 | 5 (15.2%) | 6 (20.0%) | 0.848 | 4 (12.1%) | 8 (26.7%) | 0.262 |
|  | nil | 15 (45.5%) | 11 (36.7%) |  | 23 (69.7%) | 19 (63.3%) |  | 20 (60.6%) | 13 (43.3%) |  |
|  | ≥10 increase | 16 (48.5%) | 16 (53.3%) |  | 5 (15.2%) | 5 (16.7%) |  | 9 (27.3%) | 9 (30.0%) |  |
| 5w | ≥10 decrease | 0 (0.0%) | 2 (6.3%) | 0.321 | 6 (18.2%) | 6 (18.8%) | 0.996 | 4 (12.1%) | 6 (18.8%) | 0.267 |
|  | nil | 17 (51.5%) | 14 (43.8%) |  | 22 (66.7%) | 21 (65.6%) |  | 23 (69.7%) | 16 (50.0%) |  |
|  | ≥10 increase | 16 (48.5%) | 16 (50.0%) |  | 5 (15.2%) | 5 (15.6%) |  | 6 (18.2%) | 10 (31.3%) |  |
| 6w | ≥10 decrease | 1 (3.3%) | 2 (7.4%) | 0.761 | 2 (6.7%) | 3 (11.1%) | 0.839 | 4 (13.3%) | 6 (22.2%) | 0.140 |
|  | nil | 14 (46.7%) | 13 (48.1%) |  | 21 (70.0%) | 18 (66.7%) |  | 19 (63.3%) | 10 (37.0%) |  |
|  | ≥10 increase | 15 (50.0%) | 12 (44.4%) |  | 7 (23.3%) | 6 (22.2%) |  | 7 (23.3%) | 11 (40.7%) |  |
| 7w | ≥10 decrease | 7 (33.3%) | 2 (15.4%) | 0.249 | 2 (9.5%) | 4 (30.8%) | 0.057 | 0 (0.0%) | 2 (15.4%) | 0.137 |
|  | nil | 14 (66.7%) | 11 (84.6%) |  | 10 (47.6%) | 8 (61.5%) |  | 16 (76.2%) | 7 (53.8%) |  |
|  | ≥10 increase | 0 (0.0%) | 0 (0.0%) |  | 9 (42.9%) | 1 (7.7%) |  | 5 (23.8%) | 4 (30.8%) |  |
| 1m | ≥10 decrease | 1 (4.3%) | 2 (5.6%) | 0.467 | 3 (13.0%) | 6 (16.7%) | 0.874 | 3 (13.0%) | 9 (25.0%) | 0.190 |
|  | nil | 14 (60.9%) | 16 (44.4%) |  | 18 (78.3%) | 26 (72.2%) |  | 17 (73.9%) | 18 (50.0%) |  |
|  | ≥10 increase | 8 (34.8%) | 18 (50.0%) |  | 2 (8.7%) | 4 (11.1%) |  | 3 (13.0%) | 9 (25.0%) |  |
| 3m | ≥10 decrease | 1 (4.0%) | 2 (8.3%) | 0.805 | 4 (16.0%) | 5 (20.8%) | 0.580 | 3 (12.0%) | 5 (20.8%) | 0.287 |
|  | nil | 18 (72.0%) | 17 (70.8%) |  | 18 (72.0%) | 18 (75.0%) |  | 18 (72.0%) | 12 (50.0%) |  |
|  | ≥10 increase | 6 (24.0%) | 5 (20.8%) |  | 3 (12.0%) | 1 (4.2%) |  | 4 (16.0%) | 7 (29.2%) |  |

**HN35**

Table 1: H&N35 Pain

|  | CMW (Tx) | | | SoC (Cx) | | |  |  |  |
| --- | --- | --- | --- | --- | --- | --- | --- | --- | --- |
| Time | Est mean (95%CI) | Mean change from baseline (95%CI) | p | Est mean (95%CI) | Mean change from baseline (95%CI) | p | Adjusted mean (95%CI) group difference | p# | P* |
| 0 | 26.21 (18.60, 33.81) |  |  | 19.54 (12.02, 27.07) |  |  | -3.67 (-15.58, 8.25) | 0.546 |  |
| 1 | 20.65 (13.43, 27.87) | -5.55 (-14.12, 3.02) | 0.204 | 16.54 (9.32, 23.76) | -3.01 (-11.48, 5.47) | 0.487 | -3.82 (-15.49, 7.85) | 0.521 | 0.982 |
| 2 | 36.54 (29.01, 44.07) | 10.33 (1.53, 19.13) | 0.021 | 35.13 (28.06, 42.20) | 15.59 (7.22, 23.96) | <0.001 | 0.25 (-11.65, 12.16) | 0.967 | 0.576 |
| 3 | 41.87 (34.76, 48.98) | 15.67 (7.22, 24.11) | <0.001 | 42.07 (35.14, 48.99) | 22.52 (14.29, 30.76) | <0.001 | 0.52 (-10.65, 11.69) | 0.927 | 0.533 |
| 4 | 43.28 (36.27, 50.30) | 17.08 (8.70, 25.46) | <0.001 | 42.36 (35.08, 49.65) | 22.82 (14.29, 31.36) | <0.001 | -2.45 (-13.86, 8.97) | 0.674 | 0.858 |
| 5 | 48.28 (41.05, 55.51) | 22.07 (13.56, 30.59) | <0.001 | 50.25 (43.02, 57.47) | 30.71 (22.22, 39.19) | <0.001 | -1.25 (-12.70, 10.20) | 0.831 | 0.724 |
| 6 | 51.74 (44.20, 59.28) | 25.54 (16.77, 34.30) | <0.001 | 51.70 (44.03, 59.37) | 32.16 (23.31, 41.01) | <0.001 | 1.02 (-11.01, 13.06) | 0.868 | 0.509 |
| 7 | 55.93 (47.21, 64.64) | 29.72 (19.93, 39.51) | <0.001 | 51.17 (41.56, 60.77) | 31.63 (20.99, 42.26) | <0.001 | -2.14 (-16.72, 12.44) | 0.774 | 0.853 |
| 1m | 31.47 (23.64, 39.30) | 5.26 (-3.81, 14.34) | 0.256 | 34.18 (27.26, 41.10) | 14.64 (6.43, 22.85) | <0.001 | 4.26 (-7.55, 16.08) | 0.48 | 0.258 |
| 3m | 27.92 (19.83, 36.00) | 1.71 (-7.54, 10.96) | 0.717 | 17.91 (10.09, 25.73) | -1.63 (-10.64, 7.38) | 0.723 | -6.88 (-19.55, 5.79) | 0.287 | 0.662 |

Table 2: H&N35 Swallowing

|  | CMW (Tx) | | | SoC (Cx) | | |  |  |  |
| --- | --- | --- | --- | --- | --- | --- | --- | --- | --- |
| Time | Est mean (95%CI) | Mean change from baseline (95%CI) | p | Est mean (95%CI) | Mean change from baseline (95%CI) | p | Adjusted mean (95%CI) group difference | p# | P* |
| 0 | 15.15 (7.25, 23.04) |  |  | 10.89 (3.08, 18.69) |  |  | -3.55 (-15.50, 8.40) | 0.560 |  |
| 1 | 13.89 (6.45, 21.33) | -1.26 (-10.75, 8.23) | 0.795 | 13.51 (6.06, 20.95) | 2.62 (-6.77, 12.01) | 0.585 | 3.56 (-8.09, 15.21) | 0.549 | 0.350 |
| 2 | 26.92 (19.11, 34.73) | 11.77 (2.03, 21.52) | 0.018 | 22.67 (15.41, 29.93) | 11.78 (2.51, 21.05) | 0.013 | -4.88 (-16.81, 7.06) | 0.423 | 0.864 |
| 3 | 32.46 (25.14, 39.77) | 17.31 (7.96, 26.66) | <0.001 | 36.58 (29.49, 43.66) | 25.69 (16.56, 34.82) | <0.001 | 2.69 (-8.38, 13.75) | 0.634 | 0.398 |
| 4 | 41.80 (34.60, 49.00) | 26.65 (17.37, 35.93) | <0.001 | 37.13 (29.62, 44.64) | 26.24 (16.79, 35.70) | <0.001 | -7.30 (-18.66, 4.06) | 0.208 | 0.618 |
| 5 | 47.61 (40.15, 55.06) | 32.46 (23.02, 41.89) | <0.001 | 50.17 (42.73, 57.62) | 39.29 (29.89, 48.68) | <0.001 | 1.21 (-10.19, 12.62) | 0.835 | 0.526 |
| 6 | 49.16 (41.35, 56.98) | 34.02 (24.31, 43.73) | <0.001 | 52.18 (44.21, 60.15) | 41.29 (31.48, 51.10) | <0.001 | 2.89 (-9.21, 14.99) | 0.639 | 0.408 |
| 7 | 61.37 (52.18, 70.56) | 46.22 (35.38, 57.06) | <0.001 | 49.32 (39.11, 59.53) | 38.43 (26.67, 50.19) | <0.001 | -12.55 (-27.63, 2.53) | 0.103 | 0.320 |
| 1m | 26.38 (18.22, 34.53) | 11.23 (1.18, 21.28) | 0.029 | 33.63 (26.55, 40.72) | 22.75 (13.64, 31.85) | <0.001 | 7.71 (-4.12, 19.55) | 0.201 | 0.143 |
| 3m | 25.95 (17.50, 34.41) | 10.81 (0.57, 21.05) | 0.039 | 17.91 (10.09, 25.73) | 6.88 (-3.10, 16.87) | 0.176 | -3.41 (-16.26, 9.43) | 0.602 | 0.986 |

Table 3: H&N35 Senses problems

|  | CMW (Tx) | | | SoC (Cx) | | |  |  |  |
| --- | --- | --- | --- | --- | --- | --- | --- | --- | --- |
| Time | Est mean (95%CI) | Mean change from baseline (95%CI) | p | Est mean (95%CI) | Mean change from baseline (95%CI) | p | Adjusted mean (95%CI) group difference | p# | P* |
| 0 | 11.50 (3.14, 19.87) |  |  | 13.64 (5.36, 21.92) |  |  | -0.59 (-13.49, 12.31) | 0.929 |  |
| 1 | 16.41 (8.48, 24.35) | 4.91 (-4.62, 14.44) | 0.313 | 23.66 (15.72, 31.60) | 10.02 (0.60, 19.44) | 0.037 | 3.94 (-8.70, 16.57) | 0.541 | 0.547 |
| 2 | 37.21 (28.93, 45.50) | 25.71 (15.92, 35.49) | <0.001 | 39.87 (32.10, 47.63) | 26.22 (16.92, 35.53) | <0.001 | -4.94 (-17.83, 7.95) | 0.453 | 0.568 |
| 3 | 46.64 (38.82, 54.45) | 35.13 (25.75, 44.52) | <0.001 | 50.77 (43.17, 58.38) | 37.13 (27.97, 46.29) | <0.001 | 2.52 (-9.57, 14.61) | 0.683 | 0.669 |
| 4 | 56.78 (49.08, 64.49) | 45.28 (35.96, 54.60) | <0.001 | 58.34 (50.34, 66.35) | 44.70 (35.21, 54.19) | <0.001 | -2.10 (-14.46, 10.26) | 0.739 | 0.838 |
| 5 | 59.76 (51.81, 67.70) | 48.25 (38.78, 57.72) | <0.001 | 61.99 (54.06, 69.93) | 48.35 (38.92, 57.78) | <0.001 | -3.23 (-15.63, 9.17) | 0.610 | 0.722 |
| 6 | 59.76 (51.47, 68.05) | 48.25 (38.51, 58.00) | <0.001 | 64.84 (56.40, 73.28) | 51.20 (41.36, 61.04) | <0.001 | 1.61 (-11.43, 14.64) | 0.809 | 0.775 |
| 7 | 68.18 (58.57, 77.80) | 56.68 (45.79, 67.56) | <0.001 | 55.29 (44.68, 65.90) | 41.65 (29.83, 53.47) | <0.001 | -16.80 (-32.60, -1.00) | 0.037 | 0.070 |
| 1m | 51.83 (43.21, 60.45) | 40.32 (30.23, 50.42) | <0.001 | 49.12 (41.51, 56.72) | 35.47 (26.34, 44.60) | <0.001 | -3.23 (-16.03, 9.56) | 0.621 | 0.728 |
| 3m | 42.07 (33.16, 50.97) | 30.56 (20.28, 40.84) | <0.001 | 38.48 (29.87, 47.09) | 24.84 (14.81, 34.86) | <0.001 | -1.31 (-15.04, 12.41) | 0.851 | 0.928 |

Table 4: H&N35 Speech problems

|  | CMW (Tx) | | | SoC (Cx) | | |  |  |  |
| --- | --- | --- | --- | --- | --- | --- | --- | --- | --- |
| Time | Est mean (95%CI) | Mean change from baseline (95%CI) | p | Est mean (95%CI) | Mean change from baseline (95%CI) | p | Adjusted mean (95%CI) group difference | p# | P* |
| 0 | 17.24 (9.17, 25.31) |  |  | 19.42 (11.44, 27.40) |  |  | 4.68 (-7.95, 17.32) | 0.467 |  |
| 1 | 22.40 (14.75, 30.06) | 5.16 (-3.99, 14.32) | 0.269 | 17.82 (10.16, 25.47) | -1.60 (-10.65, 7.44) | 0.728 | -1.60 (-13.97, 10.77) | 0.800 | 0.395 |
| 2 | 24.65 (16.66, 32.64) | 7.41 (-1.99, 16.81) | 0.122 | 27.06 (19.57, 34.55) | 7.64 (-1.29, 16.58) | 0.094 | 2.94 (-9.68, 15.57) | 0.648 | 0.816 |
| 3 | 28.99 (21.45, 36.53) | 11.76 (2.74, 20.77) | 0.011 | 27.38 (20.04, 34.71) | 7.96 (-0.84, 16.75) | 0.076 | -0.29 (-12.13, 11.54) | 0.961 | 0.487 |
| 4 | 36.67 (29.24, 44.10) | 19.43 (10.49, 28.38) | <0.001 | 29.91 (22.19, 37.63) | 10.49 (1.38, 19.61) | 0.024 | -9.29 (-21.39, 2.81) | 0.132 | 0.055 |
| 5 | 40.69 (33.02, 48.35) | 23.45 (14.36, 32.54) | <0.001 | 41.81 (34.15, 49.46) | 22.38 (13.33, 31.44) | <0.001 | 1.96 (-10.18, 14.10) | 0.752 | 0.709 |
| 6 | 45.60 (37.60, 53.59) | 28.36 (19.00, 37.72) | <0.001 | 47.26 (39.12, 55.40) | 27.84 (18.39, 37.29) | <0.001 | 5.18 (-7.58, 17.95) | 0.426 | 0.947 |
| 7 | 54.58 (45.32, 63.84) | 37.34 (26.89, 47.80) | <0.001 | 46.59 (36.38, 56.81) | 27.17 (15.82, 38.53) | <0.001 | -9.98 (-25.47, 5.51) | 0.206 | 0.095 |
| 1m | 31.68 (23.37, 39.99) | 14.45 (4.75, 24.14) | 0.003 | 29.00 (21.66, 36.33) | 9.58 (0.81, 18.35) | 0.032 | 2.98 (-9.55, 15.51) | 0.641 | 0.819 |
| 3m | 36.72 (28.14, 45.30) | 19.48 (9.61, 29.36) | <0.001 | 24.98 (16.68, 33.28) | 5.56 (-4.07, 15.18) | 0.258 | -5.29 (-18.74, 8.15) | 0.440 | 0.204 |

Table 5: H&N35 Trouble with social eating

|  | CMW (Tx) | | | SoC (Cx) | | |  |  |  |
| --- | --- | --- | --- | --- | --- | --- | --- | --- | --- |
| Time | Est mean (95%CI) | Mean change from baseline (95%CI) | p | Est mean (95%CI) | Mean change from baseline (95%CI) | p | Adjusted mean (95%CI) group difference | p# | P* |
| 0 | 20.26 (11.96, 28.56) |  |  | 15.26 (7.05, 23.47) |  |  | 0.96 (-11.56, 13.49) | 0.88 |  |
| 1 | 24.30 (16.45, 32.16) | 4.04 (-5.56, 13.64) | 0.410 | 15.36 (7.51, 23.22) | 0.10 (-9.40, 9.60) | 0.984 | -4.35 (-16.59, 7.88) | 0.486 | 0.487 |
| 2 | 28.54 (20.32, 36.75) | 8.27 (-1.59, 18.13) | 0.100 | 30.08 (22.40, 37.76) | 14.81 (5.44, 24.19) | 0.002 | 3.83 (-8.68, 16.34) | 0.549 | 0.711 |
| 3 | 40.34 (32.61, 48.07) | 20.08 (10.61, 29.54) | <0.001 | 41.17 (33.66, 48.69) | 25.91 (16.68, 35.14) | <0.001 | 1.24 (-10.42, 12.91) | 0.834 | 0.970 |
| 4 | 47.68 (40.06, 55.30) | 27.41 (18.02, 36.81) | <0.001 | 50.00 (42.08, 57.93) | 34.74 (25.17, 44.31) | <0.001 | 2.25 (-9.70, 14.20) | 0.712 | 0.864 |
| 5 | 47.70 (39.83, 55.57) | 27.44 (17.89, 36.98) | <0.001 | 56.64 (48.78, 64.50) | 41.37 (31.87, 50.88) | <0.001 | 10.82 (-1.17, 22.81) | 0.077 | 0.191 |
| 6 | 55.63 (47.41, 63.86) | 35.37 (25.55, 45.19) | <0.001 | 62.01 (53.64, 70.38) | 46.75 (36.82, 56.67) | <0.001 | 3.84 (-8.83, 16.50) | 0.553 | 0.713 |
| 7 | 70.12 (60.55, 79.69) | 49.86 (38.88, 60.83) | <0.001 | 58.90 (48.31, 69.48) | 43.63 (31.72, 55.54) | <0.001 | -10.71 (-26.28, 4.86) | 0.178 | 0.198 |
| 1m | 43.81 (35.25, 52.36) | 23.54 (13.37, 33.71) | <0.001 | 37.87 (30.36, 45.39) | 22.61 (13.40, 31.82) | <0.001 | -3.75 (-16.16, 8.66) | 0.554 | 0.542 |
| 3m | 40.75 (31.90, 49.60) | 20.49 (10.12, 30.85) | <0.001 | 28.86 (20.31, 37.40) | 13.59 (3.49, 23.70) | 0.008 | -3.91 (-17.30, 9.48) | 0.567 | 0.548 |

Table 6: H&N35 Trouble with social contact

|  | CMW (Tx) | | | SoC (Cx) | | |  |  |  |
| --- | --- | --- | --- | --- | --- | --- | --- | --- | --- |
| Time | Est mean (95%CI) | Mean change from baseline (95%CI) | p | Est mean (95%CI) | Mean change from baseline (95%CI) | p | Adjusted mean (95%CI) group difference | p# | P* |
| 0 | 13.89 (6.03, 21.75) |  |  | 13.35 (5.56, 21.14) |  |  | 3.99 (-8.26, 16.23) | 0.523 |  |
| 1 | 16.18 (8.69, 23.68) | 2.29 (-6.22, 10.81) | 0.598 | 8.72 (1.22, 16.21) | -4.63 (-13.05, 3.79) | 0.281 | -0.99 (-13.00, 11.02) | 0.872 | 0.468 |
| 2 | 21.00 (13.21, 28.79) | 7.11 (-1.63, 15.86) | 0.111 | 20.06 (12.71, 27.41) | 6.72 (-1.60, 15.03) | 0.113 | 2.28 (-9.95, 14.52) | 0.715 | 0.806 |
| 3 | 17.75 (10.36, 25.14) | 3.86 (-4.53, 12.25) | 0.367 | 20.06 (12.85, 27.28) | 6.72 (-1.47, 14.90) | 0.108 | 4.51 (-7.02, 16.05) | 0.443 | 0.937 |
| 4 | 24.94 (17.64, 32.23) | 11.05 (2.72, 19.38) | 0.009 | 20.95 (13.39, 28.51) | 7.60 (-0.88, 16.08) | 0.079 | -4.25 (-16.02, 7.52) | 0.479 | 0.223 |
| 5 | 28.22 (20.71, 35.72) | 14.33 (5.87, 22.79) | 0.001 | 33.37 (25.87, 40.86) | 20.02 (11.59, 28.45) | <0.001 | 9.71 (-2.10, 21.51) | 0.107 | 0.398 |
| 6 | 29.15 (21.35, 36.94) | 15.26 (6.55, 23.96) | 0.001 | 35.66 (27.74, 43.59) | 22.32 (13.52, 31.11) | <0.001 | 9.42 (-2.94, 21.78) | 0.135 | 0.438 |
| 7 | 45.11 (36.18, 54.05) | 31.22 (21.49, 40.95) | <0.001 | 29.30 (19.51, 39.09) | 15.95 (5.38, 26.52) | 0.003 | -15.96 (-30.77, -1.15) | 0.035 | 0.014 |
| 1m | 25.36 (17.28, 33.44) | 11.47 (2.45, 20.49) | 0.013 | 22.63 (15.42, 29.84) | 9.28 (1.12, 17.44) | 0.026 | 2.37 (-9.78, 14.52) | 0.702 | 0.815 |
| 3m | 23.74 (15.42, 32.07) | 9.85 (0.66, 19.04) | 0.036 | 14.74 (6.67, 22.81) | 1.39 (-7.57, 10.35) | 0.761 | -3.10 (-16.07, 9.87) | 0.639 | 0.330 |

Table 7: H&N35 Less sexuality

|  | CMW (Tx) | | | SoC (Cx) | | |  |  |  |
| --- | --- | --- | --- | --- | --- | --- | --- | --- | --- |
| Time | Est mean (95%CI) | Mean change from baseline (95%CI) | p | Est mean (95%CI) | Mean change from baseline (95%CI) | p | Adjusted mean (95%CI) group difference | p# | P* |
| 0 | 38.32 (25.14, 51.50) |  |  | 40.86 (27.77, 53.95) |  |  | 9.71 (-10.24, 29.65) | 0.340 |  |
| 1 | 55.46 (42.80, 68.11) | 17.14 (4.06, 30.21) | 0.010 | 50.78 (38.10, 63.45) | 9.92 (-3.00, 22.83) | 0.132 | 4.32 (-15.33, 23.96) | 0.667 | 0.587 |
| 2 | 60.85 (47.78, 73.93) | 22.54 (9.11, 35.96) | 0.001 | 62.61 (50.14, 75.09) | 21.75 (9.00, 34.50) | 0.001 | 3.03 (-16.90, 22.96) | 0.766 | 0.506 |
| 3 | 62.10 (49.58, 74.61) | 23.78 (10.91, 36.64) | <0.001 | 65.45 (53.16, 77.73) | 24.58 (12.04, 37.13) | <0.001 | 4.21 (-14.82, 23.24) | 0.665 | 0.567 |
| 4 | 71.71 (59.33, 84.10) | 33.39 (20.62, 46.17) | <0.001 | 70.52 (57.76, 83.28) | 29.66 (16.65, 42.67) | <0.001 | -0.61 (-19.94, 18.72) | 0.95 | 0.291 |
| 5 | 68.18 (55.50, 80.86) | 29.86 (16.89, 42.84) | <0.001 | 77.16 (64.48, 89.84) | 36.30 (23.37, 49.22) | <0.001 | 14.27 (-5.10, 33.65) | 0.149 | 0.641 |
| 6 | 76.83 (63.74, 89.92) | 38.51 (25.16, 51.87) | <0.001 | 82.56 (69.28, 95.84) | 41.70 (28.21, 55.19) | <0.001 | 9.97 (-10.12, 30.06) | 0.331 | 0.979 |
| 7 | 97.21 (82.51, 111.92) | 58.89 (43.97, 73.82) | <0.001 | 75.92 (59.96, 91.87) | 35.05 (18.83, 51.28) | <0.001 | -15.79 (-39.10, 7.53) | 0.184 | 0.031 |
| 1m | 67.84 (54.35, 81.32) | 29.52 (15.67, 43.36) | <0.001 | 64.53 (52.24, 76.81) | 23.66 (11.16, 36.17) | <0.001 | 1.10 (-18.72, 20.93) | 0.913 | 0.391 |
| 3m | 63.03 (49.19, 76.86) | 24.71 (10.61, 38.81) | 0.001 | 53.69 (40.21, 67.18) | 12.83 (-0.91, 26.58) | 0.067 | 0.73 (-20.15, 21.62) | 0.945 | 0.395 |

Table 8: H&N35 Teeth

|  | CMW (Tx) | | | SoC (Cx) | | |  |  |  |
| --- | --- | --- | --- | --- | --- | --- | --- | --- | --- |
| Time | Est mean (95%CI) | Mean change from baseline (95%CI) | p | Est mean (95%CI) | Mean change from baseline (95%CI) | p | Adjusted mean (95%CI) group difference | p# | P* |
| 0 | 23.66 (13.09, 34.23) |  |  | 15.18 (4.72, 25.65) |  |  | -5.28 (-21.48, 10.91) | 0.522 |  |
| 1 | 15.59 (5.55, 25.64) | -8.07 (-19.78, 3.65) | 0.177 | 13.66 (3.61, 23.71) | -1.53 (-13.11, 10.05) | 0.796 | 2.29 (-13.56, 18.13) | 0.777 | 0.430 |
| 2 | 18.40 (7.94, 28.87) | -5.26 (-17.29, 6.77) | 0.392 | 21.05 (11.20, 30.89) | 5.86 (-5.57, 17.30) | 0.315 | 3.28 (-12.90, 19.45) | 0.692 | 0.379 |
| 3 | 19.55 (9.65, 29.46) | -4.11 (-15.65, 7.43) | 0.485 | 14.94 (5.29, 24.60) | -0.24 (-11.50, 11.02) | 0.967 | 0.08 (-15.06, 15.23) | 0.991 | 0.564 |
| 4 | 19.35 (9.58, 29.13) | -4.31 (-15.76, 7.15) | 0.461 | 13.00 (2.86, 23.13) | -2.19 (-13.85, 9.48) | 0.713 | -6.60 (-22.09, 8.89) | 0.404 | 0.889 |
| 5 | 21.15 (11.08, 31.21) | -2.51 (-14.15, 9.12) | 0.672 | 30.43 (20.38, 40.49) | 15.25 (3.65, 26.84) | 0.010 | 6.04 (-9.50, 21.58) | 0.446 | 0.232 |
| 6 | 11.75 (1.27, 22.23) | -11.91 (-23.89, 0.06) | 0.051 | 19.34 (8.69, 30.00) | 4.16 (-7.94, 16.26) | 0.501 | 15.99 (-0.37, 32.36) | 0.055 | 0.030 |
| 7 | 19.99 (7.92, 32.06) | -3.67 (-17.05, 9.71) | 0.591 | 11.59 (-1.68, 24.86) | -3.60 (-18.13, 10.94) | 0.628 | -5.69 (-25.64, 14.25) | 0.576 | 0.971 |
| 1m | 31.06 (20.20, 41.93) | 7.40 (-5.00, 19.81) | 0.242 | 22.44 (12.79, 32.09) | 7.26 (-3.96, 18.48) | 0.205 | -6.47 (-22.53, 9.59) | 0.43 | 0.903 |
| 3m | 36.52 (25.30, 47.73) | 12.86 (0.22, 25.50) | 0.046 | 18.27 (7.41, 29.13) | 3.09 (-9.24, 15.41) | 0.624 | -17.58 (-34.84, -0.32) | 0.046 | 0.228 |

Table 9: H&N35 Opening mouth

|  | CMW (Tx) | | | SoC (Cx) | | |  |  |  |
| --- | --- | --- | --- | --- | --- | --- | --- | --- | --- |
| Time | Est mean (95%CI) | Mean change from baseline (95%CI) | p | Est mean (95%CI) | Mean change from baseline (95%CI) | p | Adjusted mean (95%CI) group difference | p# | P* |
| 0 | 18.85 (10.19, 27.50) |  |  | 11.17 (2.61, 19.73) |  |  | -4.84 (-18.55, 8.86) | 0.488 |  |
| 1 | 11.46 (3.26, 19.66) | -7.39 (-17.30, 2.53) | 0.144 | 12.84 (4.63, 21.04) | 1.67 (-8.14, 11.47) | 0.739 | 2.19 (-11.22, 15.61) | 0.749 | 0.384 |
| 2 | 21.12 (12.55, 29.69) | 2.27 (-7.91, 12.46) | 0.662 | 15.14 (7.12, 23.16) | 3.97 (-5.71, 13.65) | 0.422 | -5.00 (-18.69, 8.69) | 0.474 | 0.985 |
| 3 | 25.00 (16.93, 33.08) | 6.15 (-3.61, 15.92) | 0.217 | 20.25 (12.40, 28.11) | 9.09 (-0.45, 18.62) | 0.062 | -3.70 (-16.52, 9.12) | 0.572 | 0.884 |
| 4 | 28.43 (20.47, 36.39) | 9.59 (-0.11, 19.28) | 0.053 | 21.60 (13.33, 29.87) | 10.43 (0.55, 20.31) | 0.038 | -4.16 (-17.28, 8.95) | 0.534 | 0.932 |
| 5 | 23.11 (14.90, 31.33) | 4.26 (-5.59, 14.12) | 0.396 | 24.42 (16.22, 32.62) | 13.25 (3.44, 23.07) | 0.008 | -0.50 (-13.66, 12.65) | 0.94 | 0.587 |
| 6 | 29.04 (20.46, 37.61) | 10.19 (0.05, 20.33) | 0.049 | 28.23 (19.50, 36.96) | 17.06 (6.82, 27.31) | 0.001 | 2.67 (-11.18, 16.52) | 0.706 | 0.364 |
| 7 | 38.06 (28.10, 48.01) | 19.21 (7.88, 30.54) | 0.001 | 21.49 (10.49, 32.48) | 10.32 (-1.98, 22.62) | 0.100 | -18.45 (-35.30, -1.59) | 0.032 | 0.157 |
| 1m | 25.78 (16.86, 34.69) | 6.93 (-3.58, 17.43) | 0.196 | 17.79 (9.94, 25.64) | 6.62 (-2.89, 16.12) | 0.172 | -7.01 (-20.59, 6.58) | 0.312 | 0.791 |
| 3m | 23.83 (14.62, 33.05) | 4.99 (-5.71, 15.69) | 0.361 | 18.27 (7.41, 29.13) | -2.43 (-12.86, 8.00) | 0.648 | -10.53 (-25.13, 4.06) | 0.157 | 0.508 |

Table 10: H&N35 Dry mouth

|  | CMW (Tx) | | | SoC (Cx) | | |  |  |  |
| --- | --- | --- | --- | --- | --- | --- | --- | --- | --- |
| Time | Est mean (95%CI) | Mean change from baseline (95%CI) | p | Est mean (95%CI) | Mean change from baseline (95%CI) | p | Adjusted mean (95%CI) group difference | p# | P* |
| 0 | 18.54 (9.45, 27.63) |  |  | 22.26 (13.26, 31.25) |  |  | 5.19 (-9.02, 19.40) | 0.474 |  |
| 1 | 31.51 (22.87, 40.14) | 12.97 (2.83, 23.11) | 0.012 | 33.35 (24.71, 41.99) | 11.10 (1.07, 21.12) | 0.030 | 0.08 (-13.84, 14.01) | 0.991 | 0.531 |
| 2 | 44.95 (35.95, 53.95) | 26.41 (16.00, 36.83) | <0.001 | 52.74 (44.28, 61.20) | 30.48 (20.58, 40.38) | <0.001 | 5.89 (-8.31, 20.09) | 0.416 | 0.933 |
| 3 | 53.44 (44.93, 61.95) | 34.90 (24.92, 44.89) | <0.001 | 54.99 (46.69, 63.28) | 32.73 (22.98, 42.47) | <0.001 | -1.21 (-14.55, 12.13) | 0.859 | 0.418 |
| 4 | 54.72 (46.33, 63.12) | 36.19 (26.27, 46.10) | <0.001 | 63.09 (54.37, 71.80) | 40.83 (30.73, 50.93) | <0.001 | 5.81 (-7.82, 19.44) | 0.404 | 0.939 |
| 5 | 61.39 (52.73, 70.04) | 42.85 (32.77, 52.92) | <0.001 | 65.30 (56.66, 73.94) | 43.05 (33.01, 53.08) | <0.001 | -4.00 (-17.68, 9.67) | 0.566 | 0.254 |
| 6 | 65.84 (56.83, 74.85) | 47.30 (36.94, 57.67) | <0.001 | 71.82 (62.65, 80.98) | 49.56 (39.09, 60.04) | <0.001 | 3.96 (-10.39, 18.31) | 0.588 | 0.883 |
| 7 | 72.71 (62.31, 83.10) | 54.17 (42.58, 65.75) | <0.001 | 82.42 (70.99, 93.86) | 60.17 (47.59, 72.75) | <0.001 | 9.79 (-7.53, 27.12) | 0.268 | 0.635 |
| 1m | 64.92 (55.57, 74.27) | 46.38 (35.64, 57.12) | <0.001 | 66.36 (58.07, 74.65) | 44.10 (34.38, 53.82) | <0.001 | -2.13 (-16.23, 11.97) | 0.767 | 0.375 |
| 3m | 66.72 (57.07, 76.37) | 48.18 (37.24, 59.12) | <0.001 | 66.14 (56.79, 75.48) | 43.88 (33.22, 54.55) | <0.001 | -1.19 (-16.29, 13.90) | 0.877 | 0.461 |

Table 11: H&N35 Sticky saliva

|  | CMW (Tx) | | | SoC (Cx) | | |  |  |  |
| --- | --- | --- | --- | --- | --- | --- | --- | --- | --- |
| Time | Est mean (95%CI) | Mean change from baseline (95%CI) | p | Est mean (95%CI) | Mean change from baseline (95%CI) | p | Adjusted mean (95%CI) group difference | p# | P* |
| 0 | 18.98 (9.57, 28.38) |  |  | 21.81 (12.51, 31.11) |  |  | 4.67 (-9.79, 19.12) | 0.527 |  |
| 1 | 31.38 (22.51, 40.25) | 12.40 (1.13, 23.68) | 0.031 | 29.60 (20.73, 38.46) | 7.78 (-3.37, 18.94) | 0.172 | 3.02 (-11.09, 17.13) | 0.675 | 0.855 |
| 2 | 42.10 (32.79, 51.40) | 23.12 (11.54, 34.70) | <0.001 | 54.74 (46.08, 63.39) | 32.93 (21.91, 43.94) | <0.001 | 12.38 (-2.05, 26.82) | 0.093 | 0.397 |
| 3 | 62.09 (53.37, 70.81) | 43.11 (32.00, 54.23) | <0.001 | 57.43 (48.98, 65.88) | 35.62 (24.77, 46.47) | <0.001 | -6.41 (-19.83, 7.02) | 0.35 | 0.204 |
| 4 | 63.08 (54.50, 71.66) | 44.10 (33.07, 55.14) | <0.001 | 65.52 (56.57, 74.47) | 43.71 (32.47, 54.95) | <0.001 | 1.98 (-11.80, 15.75) | 0.779 | 0.761 |
| 5 | 71.76 (62.88, 80.65) | 52.79 (41.57, 64.00) | <0.001 | 75.26 (66.39, 84.13) | 53.45 (42.28, 64.61) | <0.001 | -1.15 (-14.97, 12.67) | 0.87 | 0.512 |
| 6 | 76.30 (66.98, 85.61) | 57.32 (45.78, 68.86) | <0.001 | 78.57 (69.07, 88.06) | 56.76 (45.10, 68.41) | <0.001 | 0.87 (-13.75, 15.50) | 0.907 | 0.68 |
| 7 | 86.16 (75.21, 97.10) | 67.18 (54.29, 80.07) | <0.001 | 83.71 (71.55, 95.86) | 61.90 (47.92, 75.87) | <0.001 | 0.64 (-17.44, 18.73) | 0.944 | 0.706 |
| 1m | 52.80 (43.08, 62.52) | 33.82 (21.88, 45.77) | <0.001 | 68.02 (59.57, 76.47) | 46.20 (35.38, 57.03) | <0.001 | 10.25 (-4.07, 24.57) | 0.161 | 0.539 |
| 3m | 56.03 (45.96, 66.10) | 37.06 (24.89, 49.23) | <0.001 | 53.68 (43.97, 63.38) | 31.87 (20.00, 43.73) | <0.001 | 0.68 (-14.81, 16.17) | 0.932 | 0.676 |

Table 12: H&N35 Coughing

|  | CMW (Tx) | | | SoC (Cx) | | |  |  |  |
| --- | --- | --- | --- | --- | --- | --- | --- | --- | --- |
| Time | Est mean (95%CI) | Mean change from baseline (95%CI) | p | Est mean (95%CI) | Mean change from baseline (95%CI) | p | Adjusted mean (95%CI) group difference | p# | P* |
| 0 | 23.28 (14.71, 31.85) |  |  | 23.84 (15.37, 32.31) |  |  | -0.02 (-13.38, 13.33) | 0.997 |  |
| 1 | 22.85 (14.76, 30.93) | -0.43 (-10.68, 9.81) | 0.934 | 27.78 (19.70, 35.86) | 3.93 (-6.21, 14.07) | 0.447 | 7.79 (-5.24, 20.82) | 0.241 | 0.349 |
| 2 | 29.22 (20.74, 37.69) | 5.94 (-4.59, 16.47) | 0.269 | 37.13 (29.24, 45.01) | 13.28 (3.27, 23.29) | 0.009 | 7.14 (-6.20, 20.48) | 0.294 | 0.397 |
| 3 | 34.45 (26.50, 42.39) | 11.17 (1.07, 21.27) | 0.030 | 34.58 (26.88, 42.28) | 10.74 (0.88, 20.60) | 0.033 | -0.04 (-12.43, 12.36) | 0.995 | 0.999 |
| 4 | 39.14 (31.32, 46.96) | 15.86 (5.84, 25.89) | 0.002 | 43.04 (34.88, 51.20) | 19.20 (8.98, 29.41) | <0.001 | 3.94 (-8.77, 16.66) | 0.544 | 0.630 |
| 5 | 48.46 (40.37, 56.56) | 25.18 (14.99, 35.37) | <0.001 | 52.33 (44.24, 60.41) | 28.48 (18.34, 38.63) | <0.001 | 2.88 (-9.88, 15.64) | 0.659 | 0.725 |
| 6 | 52.52 (44.04, 61.00) | 29.24 (18.75, 39.73) | <0.001 | 55.47 (46.82, 64.11) | 31.62 (21.03, 42.22) | <0.001 | 2.86 (-10.65, 16.38) | 0.678 | 0.736 |
| 7 | 55.60 (45.64, 65.57) | 32.32 (20.62, 44.03) | <0.001 | 61.43 (50.37, 72.49) | 37.59 (24.88, 50.29) | <0.001 | 2.90 (-13.85, 19.64) | 0.734 | 0.768 |
| 1m | 34.38 (25.53, 43.23) | 11.10 (0.25, 21.96) | 0.045 | 42.53 (34.83, 50.23) | 18.69 (8.86, 28.52) | <0.001 | 13.14 (-0.09, 26.37) | 0.052 | 0.119 |
| 3m | 34.40 (25.23, 43.57) | 11.12 (0.06, 22.19) | 0.049 | 34.46 (25.62, 43.30) | 10.62 (-0.16, 21.40) | 0.054 | 2.71 (-11.61, 17.04) | 0.710 | 0.758 |

Table 13: H&N35 Felt ill

|  | CMW (Tx) | | | SoC (Cx) | | |  |  |  |
| --- | --- | --- | --- | --- | --- | --- | --- | --- | --- |
| Time | Est mean (95%CI) | Mean change from baseline (95%CI) | p | Est mean (95%CI) | Mean change from baseline (95%CI) | p | Adjusted mean (95%CI) group difference | p# | P* |
| 0 | 17.29 (7.89, 26.68) |  |  | 14.74 (5.45, 24.03) |  |  | 0.10 (-14.39, 14.59) | 0.989 |  |
| 1 | 22.28 (13.41, 31.16) | 5.00 (-6.05, 16.05) | 0.376 | 29.84 (20.96, 38.71) | 15.10 (4.16, 26.03) | 0.007 | 8.61 (-5.56, 22.77) | 0.234 | 0.335 |
| 2 | 27.92 (18.62, 37.21) | 10.63 (-0.72, 21.98) | 0.066 | 33.06 (24.39, 41.73) | 18.32 (7.53, 29.11) | 0.001 | 3.34 (-11.14, 17.81) | 0.652 | 0.718 |
| 3 | 28.98 (20.25, 37.71) | 11.69 (0.80, 22.58) | 0.035 | 31.74 (23.27, 40.22) | 17.00 (6.38, 27.63) | 0.002 | 4.15 (-9.35, 17.65) | 0.547 | 0.636 |
| 4 | 34.21 (25.61, 42.81) | 16.92 (6.11, 27.73) | 0.002 | 41.02 (32.06, 49.98) | 26.28 (15.27, 37.29) | <0.001 | 4.62 (-9.22, 18.45) | 0.513 | 0.604 |
| 5 | 42.67 (33.78, 51.56) | 25.38 (14.40, 36.37) | <0.001 | 52.41 (43.54, 61.29) | 37.67 (26.73, 48.61) | <0.001 | 8.84 (-5.04, 22.72) | 0.212 | 0.316 |
| 6 | 35.77 (26.47, 45.07) | 18.48 (7.18, 29.79) | 0.001 | 44.22 (34.74, 53.70) | 29.48 (18.06, 40.90) | <0.001 | 6.58 (-8.07, 21.24) | 0.379 | 0.473 |
| 7 | 53.67 (42.80, 64.55) | 36.39 (23.76, 49.01) | <0.001 | 43.68 (31.63, 55.74) | 28.94 (15.24, 42.64) | <0.001 | -1.83 (-19.84, 16.18) | 0.842 | 0.854 |
| 1m | 23.34 (13.65, 33.03) | 6.05 (-5.65, 17.76) | 0.311 | 28.03 (19.56, 36.51) | 13.29 (2.70, 23.89) | 0.014 | 6.25 (-8.11, 20.62) | 0.393 | 0.491 |
| 3m | 17.89 (7.85, 27.92) | 0.60 (-11.33, 12.53) | 0.921 | 14.38 (4.70, 24.06) | 10.62 (-0.16, 21.40) | 0.054 | 0.74 (-14.75, 16.23) | 0.925 | 0.946 |

Table 14: H&N35 Pain killers

|  | CMW (Tx) | | | SoC (Cx) | | |  |  |  |
| --- | --- | --- | --- | --- | --- | --- | --- | --- | --- |
| Time | Est mean (95%CI) | Mean change from baseline (95%CI) | p | Est mean (95%CI) | Mean change from baseline (95%CI) | p | Adjusted mean (95%CI) group difference | p# | P* |
| 0 | 41.15 (26.33, 55.97) |  |  | 46.85 (32.21, 61.49) |  |  | 20.15 (-2.35, 42.66) | 0.079 |  |
| 1 | 45.47 (31.52, 59.42) | 4.32 (-13.77, 22.41) | 0.64 | 41.28 (27.34, 55.22) | -5.57 (-23.48, 12.33) | 0.542 | 9.68 (-12.24, 31.61) | 0.387 | 0.472 |
| 2 | 51.75 (37.10, 66.40) | 10.60 (-7.98, 29.18) | 0.264 | 66.66 (53.07, 80.25) | 19.80 (2.13, 37.48) | 0.028 | 22.61 (0.13, 45.09) | 0.049 | 0.867 |
| 3 | 78.26 (64.56, 91.97) | 37.11 (19.28, 54.95) | <0.001 | 67.25 (53.99, 80.51) | 20.40 (2.99, 37.81) | 0.022 | -9.78 (-30.56, 11.01) | 0.357 | 0.034 |
| 4 | 73.48 (60.00, 86.95) | 32.33 (14.63, 50.03) | <0.001 | 75.89 (61.81, 89.97) | 29.04 (11.01, 47.07) | 0.002 | 8.59 (-12.77, 29.95) | 0.431 | 0.421 |
| 5 | 77.56 (63.59, 91.53) | 36.41 (18.41, 54.40) | <0.001 | 87.17 (73.22, 101.12) | 40.32 (22.40, 58.23) | <0.001 | 12.49 (-8.95, 33.94) | 0.254 | 0.595 |
| 6 | 79.87 (65.20, 94.53) | 38.71 (20.20, 57.23) | <0.001 | 85.77 (70.81, 100.74) | 38.92 (20.22, 57.62) | <0.001 | 10.45 (-12.34, 33.25) | 0.369 | 0.516 |
| 7 | 93.55 (76.24, 110.87) | 52.40 (31.73, 73.07) | <0.001 | 83.13 (63.85, 102.41) | 36.28 (13.87, 58.69) | 0.002 | -7.89 (-36.44, 20.66) | 0.588 | 0.105 |
| 1m | 48.71 (33.39, 64.03) | 7.56 (-11.60, 26.72) | 0.439 | 44.68 (31.43, 57.94) | -2.17 (-19.53, 15.19) | 0.806 | -0.25 (-22.54, 22.03) | 0.982 | 0.166 |
| 3m | 46.03 (30.14, 61.93) | 4.88 (-14.64, 24.41) | 0.624 | 24.92 (9.62, 40.23) | -21.93 (-40.96, -2.90) | 0.024 | -9.67 (-33.90, 14.56) | 0.434 | 0.054 |

Table 15: H&N35 Nutritional supplements

|  | CMW (Tx) | | | SoC (Cx) | | |  |  |  |
| --- | --- | --- | --- | --- | --- | --- | --- | --- | --- |
| Time | Est mean (95%CI) | Mean change from baseline (95%CI) | p | Est mean (95%CI) | Mean change from baseline (95%CI) | p | Adjusted mean (95%CI) group difference | p# | P* |
| 0 | 45.21 (29.78, 60.64) |  |  | 29.14 (13.91, 44.37) |  |  | -17.39 (-41.09, 6.31) | 0.15 |  |
| 1 | 33.41 (18.95, 47.88) | -11.80 (-31.39, 7.80) | 0.238 | 44.97 (30.52, 59.42) | 15.83 (-3.57, 35.23) | 0.11 | 10.01 (-13.03, 33.06) | 0.395 | 0.082 |
| 2 | 55.58 (40.34, 70.83) | 10.37 (-9.76, 30.51) | 0.313 | 43.43 (29.38, 57.49) | 14.29 (-4.85, 33.44) | 0.143 | -10.28 (-33.96, 13.39) | 0.395 | 0.657 |
| 3 | 62.65 (48.47, 76.83) | 17.44 (-1.89, 36.77) | 0.077 | 64.71 (51.03, 78.39) | 35.57 (16.70, 54.44) | <0.001 | 3.58 (-18.22, 25.38) | 0.748 | 0.171 |
| 4 | 73.86 (59.93, 87.79) | 28.65 (9.47, 47.83) | 0.003 | 81.46 (66.85, 96.07) | 52.32 (32.78, 71.86) | <0.001 | 18.27 (-4.17, 40.71) | 0.111 | 0.022 |
| 5 | 73.86 (59.38, 88.35) | 28.65 (9.15, 48.16) | 0.004 | 74.42 (59.96, 88.88) | 45.28 (25.87, 64.69) | <0.001 | 6.19 (-16.34, 28.72) | 0.590 | 0.131 |
| 6 | 80.51 (65.24, 95.77) | 35.30 (15.23, 55.37) | 0.001 | 76.21 (60.61, 91.80) | 47.07 (26.80, 67.33) | <0.001 | -3.06 (-27.07, 20.96) | 0.803 | 0.375 |
| 7 | 77.90 (59.69, 96.11) | 32.69 (10.31, 55.07) | 0.004 | 97.97 (77.60, 118.35) | 68.84 (44.61, 93.07) | <0.001 | 30.15 (-0.21, 60.51) | 0.052 | 0.011 |
| 1m | 66.70 (50.71, 82.69) | 21.49 (0.75, 42.23) | 0.042 | 62.56 (48.88, 76.24) | 33.42 (14.59, 52.25) | 0.001 | -2.23 (-25.68, 21.22) | 0.852 | 0.342 |
| 3m | 64.57 (47.94, 81.19) | 19.36 (-1.79, 40.50) | 0.073 | 56.57 (40.59, 72.55) | 27.43 (6.83, 48.04) | 0.009 | -2.42 (-28.01, 23.18) | 0.853 | 0.371 |

Table 16: H&N35 Feeding tube

|  | CMW (Tx) | | | SoC (Cx) | | |  |  |  |
| --- | --- | --- | --- | --- | --- | --- | --- | --- | --- |
| Time | Est mean (95%CI) | Mean change from baseline (95%CI) | p | Est mean (95%CI) | Mean change from baseline (95%CI) | p | Adjusted mean (95%CI) group difference | p# | P* |
| 0 | 10.45 (-0.45, 21.35) |  |  | 4.79 (-5.97, 15.55) |  |  | 4.91 (-10.83, 20.65) | 0.541 |  |
| 1 | 10.18 (-0.10, 20.45) | -0.27 (-13.22, 12.67) | 0.967 | 1.49 (-8.79, 11.77) | -3.30 (-16.11, 9.50) | 0.613 | 1.84 (-13.49, 17.17) | 0.814 | 0.765 |
| 2 | 8.20 (-2.57, 18.97) | -2.25 (-15.55, 11.05) | 0.740 | 10.03 (0.00, 20.06) | 5.24 (-7.41, 17.88) | 0.417 | 13.08 (-2.65, 28.81) | 0.103 | 0.431 |
| 3 | 11.69 (1.58, 21.79) | 1.24 (-11.52, 14.00) | 0.849 | 4.99 (-4.81, 14.79) | 0.20 (-12.25, 12.65) | 0.975 | 6.32 (-8.21, 20.84) | 0.394 | 0.887 |
| 4 | 15.66 (5.71, 25.61) | 5.21 (-7.46, 17.88) | 0.420 | 8.33 (-2.04, 18.70) | 3.54 (-9.36, 16.44) | 0.591 | 2.54 (-12.40, 17.48) | 0.739 | 0.815 |
| 5 | 19.03 (8.74, 29.33) | 8.59 (-4.29, 21.47) | 0.191 | 9.30 (-0.98, 19.58) | 4.51 (-8.30, 17.33) | 0.490 | -0.35 (-15.35, 14.65) | 0.964 | 0.604 |
| 6 | 18.30 (7.51, 29.09) | 7.85 (-5.39, 21.10) | 0.245 | 18.06 (7.07, 29.05) | 13.27 (-0.11, 26.64) | 0.052 | 12.11 (-3.83, 28.06) | 0.137 | 0.492 |
| 7 | 23.33 (10.68, 35.98) | 12.88 (-1.91, 27.67) | 0.088 | 8.44 (-5.59, 22.47) | 3.65 (-12.40, 19.69) | 0.656 | -9.90 (-29.91, 10.11) | 0.332 | 0.223 |
| 1m | 18.28 (7.04, 29.52) | 7.83 (-5.88, 21.54) | 0.263 | 13.23 (3.44, 23.03) | 8.44 (-3.97, 20.86) | 0.183 | 9.40 (-6.19, 24.99) | 0.237 | 0.665 |
| 3m | 21.50 (9.85, 33.14) | 11.05 (-2.93, 25.02) | 0.121 | 8.80 (-2.42, 20.03) | 4.01 (-9.60, 17.63) | 0.564 | -0.29 (-17.25, 16.68) | 0.973 | 0.633 |

Table 17: H&N35 Weight loss

|  | CMW (Tx) | | | SoC (Cx) | | |  |  |  |
| --- | --- | --- | --- | --- | --- | --- | --- | --- | --- |
| Time | Est mean (95%CI) | Mean change from baseline (95%CI) | p | Est mean (95%CI) | Mean change from baseline (95%CI) | p | Adjusted mean (95%CI) group difference | p# | P* |
| 0 | 31.48 (15.51, 47.45) |  |  | 34.98 (19.20, 50.75) |  |  | 10.26 (-13.66, 34.18) | 0.4 |  |
| 1 | 27.05 (12.11, 42.00) | -4.43 (-25.11, 16.25) | 0.675 | 40.60 (25.66, 55.54) | 5.62 (-14.88, 26.13) | 0.591 | 14.92 (-8.29, 38.14) | 0.208 | 0.777 |
| 2 | 55.45 (39.67, 71.24) | 23.97 (2.72, 45.23) | 0.027 | 57.18 (42.67, 71.69) | 22.21 (1.98, 42.44) | 0.031 | 0.45 (-23.44, 24.34) | 0.97 | 0.556 |
| 3 | 66.11 (51.46, 80.76) | 34.63 (14.20, 55.05) | 0.001 | 65.06 (50.95, 79.17) | 30.09 (10.15, 50.02) | 0.003 | -9.75 (-31.66, 12.16) | 0.383 | 0.210 |
| 4 | 69.52 (55.15, 83.90) | 38.04 (17.78, 58.30) | <0.001 | 84.26 (69.15, 99.36) | 49.28 (28.64, 69.92) | <0.001 | 9.54 (-13.06, 32.14) | 0.408 | 0.965 |
| 5 | 75.90 (60.94, 90.86) | 44.42 (23.80, 65.03) | <0.001 | 93.16 (78.22, 108.11) | 58.19 (37.68, 78.70) | <0.001 | 13.45 (-9.23, 36.14) | 0.245 | 0.845 |
| 6 | 77.07 (61.28, 92.86) | 45.59 (24.38, 66.80) | <0.001 | 101.14 (84.99, 117.30) | 66.17 (44.76, 87.57) | <0.001 | 22.13 (-2.13, 46.39) | 0.074 | 0.481 |
| 7 | 91.44 (72.51, 110.37) | 59.96 (36.33, 83.59) | <0.001 | 93.33 (72.07, 114.58) | 58.35 (32.80, 83.90) | <0.001 | -2.22 (-33.14, 28.70) | 0.888 | 0.521 |
| 1m | 61.83 (45.26, 78.41) | 30.35 (8.45, 52.25) | 0.007 | 58.97 (44.86, 73.08) | 23.99 (4.09, 43.90) | 0.018 | -2.05 (-25.71, 21.60) | 0.865 | 0.459 |
| 3m | 45.14 (27.88, 62.40) | 13.66 (-8.67, 35.99) | 0.23 | 40.97 (24.41, 57.53) | 5.99 (-15.76, 27.75) | 0.589 | -2.12 (-28.04, 23.79) | 0.873 | 0.478 |

Table 18: H&N35 Weight gain

|  | CMW (Tx) | | | SoC (Cx) | | |  |  |  |
| --- | --- | --- | --- | --- | --- | --- | --- | --- | --- |
| Time | Est mean (95%CI) | Mean change from baseline (95%CI) | p | Est mean (95%CI) | Mean change from baseline (95%CI) | p | Adjusted mean (95%CI) group difference | p# | P* |
| 0 | 20.41 (4.64, 36.18) |  |  | 26.47 (10.89, 42.04) |  |  | 6.03 (-17.99, 30.05) | 0.623 |  |
| 1 | 24.95 (10.20, 39.70) | 4.54 (-16.00, 25.08) | 0.665 | 35.63 (20.88, 50.37) | 9.16 (-11.21, 29.53) | 0.378 | 8.95 (-14.38, 32.29) | 0.452 | 0.857 |
| 2 | 18.51 (2.92, 34.09) | -1.91 (-23.03, 19.21) | 0.860 | 21.10 (6.79, 35.41) | -5.37 (-25.46, 14.73) | 0.601 | 1.54 (-22.45, 25.53) | 0.900 | 0.785 |
| 3 | 21.85 (7.39, 36.30) | 1.43 (-18.86, 21.73) | 0.890 | 12.39 (-1.52, 26.31) | -14.07 (-33.88, 5.73) | 0.164 | -6.56 (-28.61, 15.49) | 0.560 | 0.424 |
| 4 | 22.21 (8.04, 36.39) | 1.80 (-18.32, 21.92) | 0.861 | 10.58 (-4.33, 25.49) | -15.89 (-36.40, 4.62) | 0.129 | -10.59 (-33.32, 12.14) | 0.361 | 0.299 |
| 5 | 19.58 (4.82, 34.33) | -0.83 (-21.31, 19.65) | 0.936 | 12.27 (-2.48, 27.03) | -14.20 (-34.58, 6.19) | 0.172 | -5.44 (-28.27, 17.39) | 0.640 | 0.475 |
| 6 | 14.56 (-1.04, 30.17) | -5.85 (-26.93, 15.23) | 0.586 | 13.93 (-2.02, 29.89) | -12.54 (-33.80, 8.72) | 0.248 | 7.25 (-17.10, 31.60) | 0.559 | 0.941 |
| 7 | 31.01 (12.29, 49.74) | 10.60 (-12.87, 34.07) | 0.376 | 24.85 (3.82, 45.88) | -1.62 (-26.99, 23.75) | 0.900 | -3.37 (-34.27, 27.53) | 0.831 | 0.625 |
| 1m | 31.24 (14.86, 47.61) | 10.82 (-10.93, 32.58) | 0.329 | 38.67 (24.76, 52.59) | 12.21 (-7.57, 31.98) | 0.226 | 6.51 (-17.29, 30.32) | 0.592 | 0.976 |
| 3m | 45.01 (27.96, 62.06) | 24.60 (2.42, 46.77) | 0.030 | 34.93 (18.57, 51.29) | 8.47 (-13.14, 30.07) | 0.442 | -3.00 (-28.98, 22.98) | 0.821 | 0.600 |

**HN35 10-point change**

Table 1: HN35 item score change categories from baseline

|  |  | H&N35: Pain | | | H&N35: Swallowing | | | H&N35: Senses problems | | |
| --- | --- | --- | --- | --- | --- | --- | --- | --- | --- | --- |
|  | Score change | CMW (Tx) | SoC (Cx) |  | CMW (Tx) | SoC (Cx) |  | CMW (Tx) | SoC (Cx) |  |
|  |  | N (%) | N (%) | p | N (%) | N (%) | p | N (%) | N (%) | p |
| 1w | ≥10 decrease | 7 (23.3%) | 11 (34.4%) | 0.622 | 1 (3.3%) | 4 (12.5%) | 0.275 | 5 (16.7%) | 4 (12.5%) | 0.688 |
|  | nil | 17 (56.7%) | 15 (46.9%) |  | 27 (90.0%) | 24 (75.0%) |  | 15 (50.0%) | 14 (43.8%) |  |
|  | ≥10 increase | 6 (20.0%) | 6 (18.8%) |  | 2 (6.7%) | 4 (12.5%) |  | 10 (33.3%) | 14 (43.8%) |  |
| 2w | ≥10 decrease | 5 (17.9%) | 5 (15.6%) | 0.922 | 1 (3.6%) | 4 (12.5%) | 0.413 | 1 (3.6%) | 4 (12.5%) | 0.013 |
|  | nil | 13 (46.4%) | 14 (43.8%) |  | 18 (64.3%) | 17 (53.1%) |  | 14 (50.0%) | 5 (15.6%) |  |
|  | ≥10 increase | 10 (35.7%) | 13 (40.6%) |  | 9 (32.1%) | 11 (34.4%) |  | 13 (46.4%) | 23 (71.9%) |  |
| 3w | ≥10 decrease | 3 (9.1%) | 3 (8.8%) | 0.936 | 1 (3.0%) | 1 (2.9%) | 0.995 | 1 (3.0%) | 0 (0.0%) | 0.434 |
|  | nil | 11 (33.3%) | 10 (29.4%) |  | 13 (39.4%) | 13 (38.2%) |  | 6 (18.2%) | 4 (11.8%) |  |
|  | ≥10 increase | 19 (57.6%) | 21 (61.8%) |  | 19 (57.6%) | 20 (58.8%) |  | 26 (78.8%) | 30 (88.2%) |  |
| 4w | ≥10 decrease | 5 (15.2%) | 2 (6.7%) | 0.543 | 2 (6.1%) | 2 (6.7%) | 0.708 | 3 (9.1%) | 0 (0.0%) | 0.143 |
|  | nil | 11 (33.3%) | 10 (33.3%) |  | 8 (24.2%) | 10 (33.3%) |  | 0 (0.0%) | 1 (3.3%) |  |
|  | ≥10 increase | 17 (51.5%) | 18 (60.0%) |  | 23 (69.7%) | 18 (60.0%) |  | 30 (90.9%) | 29 (96.7%) |  |
| 5w | ≥10 decrease | 2 (6.1%) | 2 (6.3%) | 0.277 | 1 (3.0%) | 0 (0.0%) | 0.426 | 4 (12.1%) | 2 (6.3%) | 0.414 |
|  | nil | 12 (36.4%) | 6 (18.8%) |  | 10 (30.3%) | 7 (21.9%) |  | 29 (87.9%) | 30 (93.8%) |  |
|  | ≥10 increase | 19 (57.6%) | 24 (75.0%) |  | 22 (66.7%) | 25 (78.1%) |  | 0 (0.0%) | 0 (0.0%) |  |
| 6w | ≥10 decrease | 1 (3.3%) | 1 (3.7%) | 0.927 | 3 (10.0%) | 0 (0.0%) | 0.179 | 1 (3.3%) | 0 (0.0%) | 0.632 |
|  | nil | 8 (26.7%) | 6 (22.2%) |  | 5 (16.7%) | 3 (11.1%) |  | 1 (3.3%) | 1 (3.7%) |  |
|  | ≥10 increase | 21 (70.0%) | 20 (74.1%) |  | 22 (73.3%) | 24 (88.9%) |  | 28 (93.3%) | 26 (96.3%) |  |
| 7w | ≥10 decrease | 1 (4.8%) | 0 (0.0%) | 0.711 | 0 (0.0%) | 0 (0.0%) | 0.785 | 0 (0.0%) | 2 (15.4%) | 0.064 |
|  | nil | 4 (19.0%) | 3 (23.1%) |  | 4 (19.0%) | 2 (15.4%) |  | 21 (100.0%) | 11 (84.6%) |  |
|  | ≥10 increase | 16 (76.2%) | 10 (76.9%) |  | 17 (81.0%) | 11 (84.6%) |  | 0 (0.0%) | 0 (0.0%) |  |
| 1m | ≥10 decrease | 7 (30.4%) | 5 (13.9%) | 0.304 | 5 (21.7%) | 3 (8.3%) | 0.277 | 1 (4.3%) | 2 (5.6%) | 0.111 |
|  | nil | 8 (34.8%) | 16 (44.4%) |  | 11 (47.8%) | 17 (47.2%) |  | 0 (0.0%) | 6 (16.7%) |  |
|  | ≥10 increase | 8 (34.8%) | 15 (41.7%) |  | 7 (30.4%) | 16 (44.4%) |  | 22 (95.7%) | 28 (77.8%) |  |
| 3m | ≥10 decrease | 6 (24.0%) | 5 (20.8%) | 0.703 | 4 (16.0%) | 2 (8.3%) | 0.652 | 2 (8.0%) | 2 (8.3%) | 0.995 |
|  | nil | 16 (64.0%) | 14 (58.3%) |  | 13 (52.0%) | 15 (62.5%) |  | 6 (24.0%) | 6 (25.0%) |  |
|  | ≥10 increase | 3 (12.0%) | 5 (20.8%) |  | 8 (32.0%) | 7 (29.2%) |  | 17 (68.0%) | 16 (66.7%) |  |

|  |  | H&N35: Speech problems | | | H&N35: Trouble with social eating | | | H&N35: Trouble with social contact | | |
| --- | --- | --- | --- | --- | --- | --- | --- | --- | --- | --- |
|  | Score change | CMW (Tx) | SoC (Cx) |  | CMW (Tx) | SoC (Cx) |  | CMW (Tx) | SoC (Cx) |  |
|  |  | N (%) | N (%) | p | N (%) | N (%) | p | N (%) | N (%) | p |
| 1w | ≥10 decrease | 6 (20.0%) | 9 (28.1%) | 0.42 | 4 (13.3%) | 5 (15.6%) | 0.883 | 4 (13.3%) | 8 (25.0%) | 0.448 |
|  | nil | 13 (43.3%) | 16 (50.0%) |  | 21 (70.0%) | 23 (71.9%) |  | 19 (63.3%) | 19 (59.4%) |  |
|  | ≥10 increase | 11 (36.7%) | 7 (21.9%) |  | 5 (16.7%) | 4 (12.5%) |  | 7 (23.3%) | 5 (15.6%) |  |
| 2w | ≥10 decrease | 5 (17.9%) | 7 (21.9%) | 0.927 | 5 (17.9%) | 2 (6.3%) | 0.259 | 4 (14.3%) | 7 (21.9%) | 0.569 |
|  | nil | 10 (35.7%) | 11 (34.4%) |  | 15 (53.6%) | 16 (50.0%) |  | 15 (53.6%) | 13 (40.6%) |  |
|  | ≥10 increase | 13 (46.4%) | 14 (43.8%) |  | 8 (28.6%) | 14 (43.8%) |  | 9 (32.1%) | 12 (37.5%) |  |
| 3w | ≥10 decrease | 5 (15.2%) | 8 (23.5%) | 0.613 | 3 (9.1%) | 1 (2.9%) | 0.569 | 2 (6.1%) | 6 (17.6%) | 0.012 |
|  | nil | 8 (24.2%) | 9 (26.5%) |  | 9 (27.3%) | 10 (29.4%) |  | 27 (81.8%) | 16 (47.1%) |  |
|  | ≥10 increase | 20 (60.6%) | 17 (50.0%) |  | 21 (63.6%) | 23 (67.6%) |  | 4 (12.1%) | 12 (35.3%) |  |
| 4w | ≥10 decrease | 2 (6.1%) | 6 (20.0%) | 0.252 | 2 (6.1%) | 2 (6.7%) | 0.599 | 3 (9.1%) | 6 (20.0%) | 0.462 |
|  | nil | 9 (27.3%) | 7 (23.3%) |  | 9 (27.3%) | 5 (16.7%) |  | 18 (54.5%) | 14 (46.7%) |  |
|  | ≥10 increase | 22 (66.7%) | 17 (56.7%) |  | 22 (66.7%) | 23 (76.7%) |  | 12 (36.4%) | 10 (33.3%) |  |
| 5w | ≥10 decrease | 3 (9.1%) | 6 (18.8%) | 0.017 | 1 (3.0%) | 1 (3.1%) | 0.919 | 0 (0.0%) | 3 (9.4%) | 0.193 |
|  | nil | 2 (6.1%) | 9 (28.1%) |  | 4 (12.1%) | 5 (15.6%) |  | 18 (54.5%) | 15 (46.9%) |  |
|  | ≥10 increase | 28 (84.8%) | 17 (53.1%) |  | 28 (84.8%) | 26 (81.3%) |  | 15 (45.5%) | 14 (43.8%) |  |
| 6w | ≥10 decrease | 3 (10.0%) | 5 (18.5%) | 0.485 | 4 (13.3%) | 1 (3.7%) | 0.439 | 2 (6.7%) | 3 (11.1%) | 0.701 |
|  | nil | 6 (20.0%) | 3 (11.1%) |  | 4 (13.3%) | 4 (14.8%) |  | 14 (46.7%) | 10 (37.0%) |  |
|  | ≥10 increase | 21 (70.0%) | 19 (70.4%) |  | 22 (73.3%) | 22 (81.5%) |  | 14 (46.7%) | 14 (51.9%) |  |
| 7w | ≥10 decrease | 3 (14.3%) | 2 (15.4%) | 0.359 | 1 (4.8%) | 1 (7.7%) | 0.724 | 1 (4.8%) | 1 (7.7%) | 0.924 |
|  | nil | 3 (14.3%) | 0 (0.0%) |  | 0 (0.0%) | 0 (0.0%) |  | 11 (52.4%) | 7 (53.8%) |  |
|  | ≥10 increase | 15 (71.4%) | 11 (84.6%) |  | 20 (95.2%) | 12 (92.3%) |  | 9 (42.9%) | 5 (38.5%) |  |
| 1m | ≥10 decrease | 5 (21.7%) | 8 (22.2%) | 0.942 | 2 (8.7%) | 1 (2.8%) | 0.516 | 2 (8.7%) | 6 (16.7%) | 0.288 |
|  | nil | 6 (26.1%) | 8 (22.2%) |  | 8 (34.8%) | 16 (44.4%) |  | 15 (65.2%) | 16 (44.4%) |  |
|  | ≥10 increase | 12 (52.2%) | 20 (55.6%) |  | 13 (56.5%) | 19 (52.8%) |  | 6 (26.1%) | 14 (38.9%) |  |
| 3m | ≥10 decrease | 5 (20.0%) | 5 (20.8%) | 0.138 | 2 (8.0%) | 0 (0.0%) | 0.034 | 1 (4.0%) | 3 (12.5%) | 0.494 |
|  | nil | 2 (8.0%) | 7 (29.2%) |  | 9 (36.0%) | 17 (70.8%) |  | 18 (72.0%) | 17 (70.8%) |  |
|  | ≥10 increase | 18 (72.0%) | 12 (50.0%) |  | 14 (56.0%) | 7 (29.2%) |  | 6 (24.0%) | 4 (16.7%) |  |

|  |  | H&N35: Less sexuality | | | H&N35: Teeth | | | H&N35: Opening mouth | | |
| --- | --- | --- | --- | --- | --- | --- | --- | --- | --- | --- |
|  | Score change | CMW (Tx) | SoC (Cx) |  | CMW (Tx) | SoC (Cx) |  | CMW (Tx) | SoC (Cx) |  |
|  |  | N (%) | N (%) | p | N (%) | N (%) | p | N (%) | N (%) | p |
| 1w | ≥10 decrease | 4 (13.3%) | 4 (12.5%) | 0.995 | 5 (16.7%) | 6 (18.8%) | 0.235 | 5 (16.7%) | 4 (12.5%) | 0.871 |
|  | nil | 14 (46.7%) | 15 (46.9%) |  | 24 (80.0%) | 21 (65.6%) |  | 22 (73.3%) | 24 (75.0%) |  |
|  | ≥10 increase | 12 (40.0%) | 13 (40.6%) |  | 1 (3.3%) | 5 (15.6%) |  | 3 (10.0%) | 4 (12.5%) |  |
| 2w | ≥10 decrease | 2 (7.1%) | 3 (9.4%) | 0.775 | 7 (25.0%) | 5 (15.6%) | 0.159 | 4 (14.3%) | 6 (18.8%) | 0.765 |
|  | nil | 13 (46.4%) | 17 (53.1%) |  | 19 (67.9%) | 19 (59.4%) |  | 20 (71.4%) | 20 (62.5%) |  |
|  | ≥10 increase | 13 (46.4%) | 12 (37.5%) |  | 2 (7.1%) | 8 (25.0%) |  | 4 (14.3%) | 6 (18.8%) |  |
| 3w | ≥10 decrease | 4 (12.1%) | 3 (8.8%) | 0.79 | 6 (18.2%) | 8 (23.5%) | 0.779 | 4 (12.1%) | 4 (11.8%) | 0.931 |
|  | nil | 16 (48.5%) | 15 (44.1%) |  | 24 (72.7%) | 22 (64.7%) |  | 21 (63.6%) | 23 (67.6%) |  |
|  | ≥10 increase | 13 (39.4%) | 16 (47.1%) |  | 3 (9.1%) | 4 (11.8%) |  | 8 (24.2%) | 7 (20.6%) |  |
| 4w | ≥10 decrease | 2 (6.1%) | 2 (6.7%) | 0.929 | 7 (21.2%) | 8 (26.7%) | 0.621 | 6 (18.2%) | 4 (13.3%) | 0.868 |
|  | nil | 14 (42.4%) | 14 (46.7%) |  | 20 (60.6%) | 19 (63.3%) |  | 20 (60.6%) | 19 (63.3%) |  |
|  | ≥10 increase | 17 (51.5%) | 14 (46.7%) |  | 6 (18.2%) | 3 (10.0%) |  | 7 (21.2%) | 7 (23.3%) |  |
| 5w | ≥10 decrease | 5 (15.2%) | 1 (3.1%) | 0.093 | 6 (18.2%) | 4 (12.5%) | 0.524 | 4 (12.1%) | 5 (15.6%) | 0.244 |
|  | nil | 15 (45.5%) | 11 (34.4%) |  | 23 (69.7%) | 21 (65.6%) |  | 23 (69.7%) | 16 (50.0%) |  |
|  | ≥10 increase | 13 (39.4%) | 20 (62.5%) |  | 4 (12.1%) | 7 (21.9%) |  | 6 (18.2%) | 11 (34.4%) |  |
| 6w | ≥10 decrease | 4 (13.3%) | 1 (3.7%) | 0.373 | 6 (20.0%) | 4 (14.8%) | 0.614 | 2 (6.7%) | 2 (7.4%) | 0.991 |
|  | nil | 11 (36.7%) | 9 (33.3%) |  | 21 (70.0%) | 18 (66.7%) |  | 16 (53.3%) | 14 (51.9%) |  |
|  | ≥10 increase | 15 (50.0%) | 17 (63.0%) |  | 3 (10.0%) | 5 (18.5%) |  | 12 (40.0%) | 11 (40.7%) |  |
| 7w | ≥10 decrease | 0 (0.0%) | 0 (0.0%) | 0.362 | 7 (33.3%) | 2 (15.4%) | 0.040 | 3 (14.3%) | 2 (15.4%) | 0.813 |
|  | nil | 5 (23.8%) | 5 (38.5%) |  | 9 (42.9%) | 11 (84.6%) |  | 11 (52.4%) | 8 (61.5%) |  |
|  | ≥10 increase | 16 (76.2%) | 8 (61.5%) |  | 5 (23.8%) | 0 (0.0%) |  | 7 (33.3%) | 3 (23.1%) |  |
| 1m | ≥10 decrease | 1 (4.3%) | 5 (13.9%) | 0.489 | 6 (26.1%) | 4 (11.1%) | 0.084 | 6 (26.1%) | 6 (16.7%) | 0.668 |
|  | nil | 8 (34.8%) | 12 (33.3%) |  | 10 (43.5%) | 26 (72.2%) |  | 12 (52.2%) | 22 (61.1%) |  |
|  | ≥10 increase | 14 (60.9%) | 19 (52.8%) |  | 7 (30.4%) | 6 (16.7%) |  | 5 (21.7%) | 8 (22.2%) |  |
| 3m | ≥10 decrease | 2 (8.0%) | 3 (12.5%) | 0.414 | 5 (20.0%) | 3 (12.5%) | 0.289 | 6 (24.0%) | 5 (20.8%) | 0.703 |
|  | nil | 10 (40.0%) | 13 (54.2%) |  | 10 (40.0%) | 15 (62.5%) |  | 14 (56.0%) | 16 (66.7%) |  |
|  | ≥10 increase | 13 (52.0%) | 8 (33.3%) |  | 10 (40.0%) | 6 (25.0%) |  | 5 (20.0%) | 3 (12.5%) |  |

|  |  | H&N35: Dry mouth | | | H&N35: Sticky saliva | | | H&N35: Coughing | | |
| --- | --- | --- | --- | --- | --- | --- | --- | --- | --- | --- |
|  | Score change | CMW (Tx) | SoC (Cx) |  | CMW (Tx) | SoC (Cx) |  | CMW (Tx) | SoC (Cx) |  |
|  |  | N (%) | N (%) | p | N (%) | N (%) | p | N (%) | N (%) | p |
| 1w | ≥10 decrease | 4 (13.3%) | 5 (15.6%) | 0.959 | 1 (3.3%) | 5 (15.6%) | 0.262 | 7 (23.3%) | 5 (15.6%) | 0.638 |
|  | nil | 13 (43.3%) | 13 (40.6%) |  | 16 (53.3%) | 15 (46.9%) |  | 17 (56.7%) | 18 (56.3%) |  |
|  | ≥10 increase | 13 (43.3%) | 14 (43.8%) |  | 13 (43.3%) | 12 (37.5%) |  | 6 (20.0%) | 9 (28.1%) |  |
| 2w | ≥10 decrease | 1 (3.6%) | 4 (12.5%) | 0.425 | 1 (3.6%) | 3 (9.4%) | 0.069 | 6 (21.4%) | 6 (18.8%) | 0.193 |
|  | nil | 7 (25.0%) | 6 (18.8%) |  | 15 (53.6%) | 8 (25.0%) |  | 18 (64.3%) | 15 (46.9%) |  |
|  | ≥10 increase | 20 (71.4%) | 22 (68.8%) |  | 12 (42.9%) | 21 (65.6%) |  | 4 (14.3%) | 11 (34.4%) |  |
| 3w | ≥10 decrease | 1 (3.0%) | 3 (8.8%) | 0.588 | 2 (6.1%) | 2 (5.9%) | 0.589 | 5 (15.2%) | 6 (17.6%) | 0.928 |
|  | nil | 7 (21.2%) | 6 (17.6%) |  | 3 (9.1%) | 6 (17.6%) |  | 17 (51.5%) | 16 (47.1%) |  |
|  | ≥10 increase | 25 (75.8%) | 25 (73.5%) |  | 28 (84.8%) | 26 (76.5%) |  | 11 (33.3%) | 12 (35.3%) |  |
| 4w | ≥10 decrease | 1 (3.0%) | 2 (6.7%) | 0.774 | 2 (6.1%) | 2 (6.7%) | 0.865 | 2 (6.1%) | 3 (10.0%) | 0.73 |
|  | nil | 5 (15.2%) | 5 (16.7%) |  | 4 (12.1%) | 5 (16.7%) |  | 15 (45.5%) | 15 (50.0%) |  |
|  | ≥10 increase | 27 (81.8%) | 23 (76.7%) |  | 27 (81.8%) | 23 (76.7%) |  | 16 (48.5%) | 12 (40.0%) |  |
| 5w | ≥10 decrease | 1 (3.0%) | 4 (12.5%) | 0.347 | 1 (3.0%) | 4 (12.5%) | 0.307 | 3 (9.1%) | 3 (9.4%) | 0.988 |
|  | nil | 6 (18.2%) | 6 (18.8%) |  | 5 (15.2%) | 3 (9.4%) |  | 13 (39.4%) | 12 (37.5%) |  |
|  | ≥10 increase | 26 (78.8%) | 22 (68.8%) |  | 27 (81.8%) | 25 (78.1%) |  | 17 (51.5%) | 17 (53.1%) |  |
| 6w | ≥10 decrease | 1 (3.3%) | 3 (11.1%) | 0.508 | 1 (3.3%) | 1 (3.7%) | 0.834 | 1 (3.3%) | 3 (11.1%) | 0.332 |
|  | nil | 2 (6.7%) | 2 (7.4%) |  | 2 (6.7%) | 3 (11.1%) |  | 8 (26.7%) | 4 (14.8%) |  |
|  | ≥10 increase | 27 (90.0%) | 22 (81.5%) |  | 27 (90.0%) | 23 (85.2%) |  | 21 (70.0%) | 20 (74.1%) |  |
| 7w | ≥10 decrease | 0 (0.0%) | 2 (15.4%) | 0.142 | 0 (0.0%) | 3 (23.1%) | 0.070 | 0 (0.0%) | 1 (7.7%) | 0.419 |
|  | nil | 2 (9.5%) | 2 (15.4%) |  | 2 (9.5%) | 1 (7.7%) |  | 8 (38.1%) | 4 (30.8%) |  |
|  | ≥10 increase | 19 (90.5%) | 9 (69.2%) |  | 19 (90.5%) | 9 (69.2%) |  | 13 (61.9%) | 8 (61.5%) |  |
| 1m | ≥10 decrease | 1 (4.3%) | 5 (13.9%) | 0.374 | 2 (8.7%) | 3 (8.3%) | 0.669 | 5 (21.7%) | 6 (16.7%) | 0.441 |
|  | nil | 2 (8.7%) | 5 (13.9%) |  | 6 (26.1%) | 6 (16.7%) |  | 11 (47.8%) | 13 (36.1%) |  |
|  | ≥10 increase | 20 (87.0%) | 26 (72.2%) |  | 15 (65.2%) | 27 (75.0%) |  | 7 (30.4%) | 17 (47.2%) |  |
| 3m | ≥10 decrease | 0 (0.0%) | 2 (8.3%) | 0.329 | 2 (8.0%) | 4 (16.7%) | 0.603 | 6 (24.0%) | 5 (20.8%) | 0.782 |
|  | nil | 4 (16.0%) | 3 (12.5%) |  | 7 (28.0%) | 5 (20.8%) |  | 11 (44.0%) | 9 (37.5%) |  |
|  | ≥10 increase | 21 (84.0%) | 19 (79.2%) |  | 16 (64.0%) | 15 (62.5%) |  | 8 (32.0%) | 10 (41.7%) |  |

|  |  | H&N35: Felt ill | | | H&N35: Pain killers | | | H&N35: Nutritional supplements | | |
| --- | --- | --- | --- | --- | --- | --- | --- | --- | --- | --- |
|  | Score change | CMW (Tx) | SoC (Cx) |  | CMW (Tx) | SoC (Cx) |  | CMW (Tx) | SoC (Cx) |  |
|  |  | N (%) | N (%) | p | N (%) | N (%) | p | N (%) | N (%) | p |
| 1w | ≥10 decrease | 4 (13.3%) | 5 (15.6%) | 0.587 | 5 (16.7%) | 4 (12.5%) | 0.770 | 6 (20.0%) | 2 (6.3%) | 0.015 |
|  | nil | 17 (56.7%) | 14 (43.8%) |  | 20 (66.7%) | 24 (75.0%) |  | 23 (76.7%) | 21 (65.6%) |  |
|  | ≥10 increase | 9 (30.0%) | 13 (40.6%) |  | 5 (16.7%) | 4 (12.5%) |  | 1 (3.3%) | 9 (28.1%) |  |
| 2w | ≥10 decrease | 2 (7.1%) | 4 (12.5%) | 0.262 | 2 (7.1%) | 3 (9.4%) | 0.297 | 4 (14.3%) | 4 (12.5%) | 0.920 |
|  | nil | 19 (67.9%) | 15 (46.9%) |  | 21 (75.0%) | 18 (56.3%) |  | 19 (67.9%) | 21 (65.6%) |  |
|  | ≥10 increase | 7 (25.0%) | 13 (40.6%) |  | 5 (17.9%) | 11 (34.4%) |  | 5 (17.9%) | 7 (21.9%) |  |
| 3w | ≥10 decrease | 3 (9.1%) | 3 (8.8%) | 0.930 | 1 (3.0%) | 5 (14.7%) | 0.152 | 5 (15.2%) | 4 (11.8%) | 0.504 |
|  | nil | 15 (45.5%) | 17 (50.0%) |  | 17 (51.5%) | 19 (55.9%) |  | 18 (54.5%) | 15 (44.1%) |  |
|  | ≥10 increase | 15 (45.5%) | 14 (41.2%) |  | 15 (45.5%) | 10 (29.4%) |  | 10 (30.3%) | 15 (44.1%) |  |
| 4w | ≥10 decrease | 3 (9.1%) | 2 (6.7%) | 0.671 | 2 (6.1%) | 5 (16.7%) | 0.404 | 3 (9.1%) | 3 (10.0%) | 0.313 |
|  | nil | 15 (45.5%) | 11 (36.7%) |  | 18 (54.5%) | 15 (50.0%) |  | 16 (48.5%) | 9 (30.0%) |  |
|  | ≥10 increase | 15 (45.5%) | 17 (56.7%) |  | 13 (39.4%) | 10 (33.3%) |  | 14 (42.4%) | 18 (60.0%) |  |
| 5w | ≥10 decrease | 1 (3.0%) | 1 (3.1%) | 0.909 | 1 (3.0%) | 1 (3.1%) | 0.936 | 4 (12.1%) | 4 (12.5%) | 0.630 |
|  | nil | 12 (36.4%) | 10 (31.3%) |  | 19 (57.6%) | 17 (53.1%) |  | 14 (42.4%) | 10 (31.3%) |  |
|  | ≥10 increase | 20 (60.6%) | 21 (65.6%) |  | 13 (39.4%) | 14 (43.8%) |  | 15 (45.5%) | 18 (56.3%) |  |
| 6w | ≥10 decrease | 2 (6.7%) | 2 (7.4%) | 0.954 | 3 (10.0%) | 3 (11.1%) | 0.977 | 3 (10.0%) | 4 (14.8%) | 0.813 |
|  | nil | 10 (33.3%) | 8 (29.6%) |  | 14 (46.7%) | 13 (48.1%) |  | 13 (43.3%) | 10 (37.0%) |  |
|  | ≥10 increase | 18 (60.0%) | 17 (63.0%) |  | 13 (43.3%) | 11 (40.7%) |  | 14 (46.7%) | 13 (48.1%) |  |
| 7w | ≥10 decrease | 0 (0.0%) | 1 (7.7%) | 0.389 | 2 (9.5%) | 2 (15.4%) | 0.608 | 2 (9.5%) | 1 (7.7%) | 0.309 |
|  | nil | 7 (33.3%) | 5 (38.5%) |  | 9 (42.9%) | 7 (53.8%) |  | 10 (47.6%) | 3 (23.1%) |  |
|  | ≥10 increase | 14 (66.7%) | 7 (53.8%) |  | 10 (47.6%) | 4 (30.8%) |  | 9 (42.9%) | 9 (69.2%) |  |
| 1m | ≥10 decrease | 4 (17.4%) | 4 (11.1%) | 0.750 | 3 (13.0%) | 8 (22.2%) | 0.665 | 3 (13.0%) | 3 (8.3%) | 0.802 |
|  | nil | 11 (47.8%) | 20 (55.6%) |  | 16 (69.6%) | 23 (63.9%) |  | 13 (56.5%) | 20 (55.6%) |  |
|  | ≥10 increase | 8 (34.8%) | 12 (33.3%) |  | 4 (17.4%) | 5 (13.9%) |  | 7 (30.4%) | 13 (36.1%) |  |
| 3m | ≥10 decrease | 6 (24.0%) | 3 (12.5%) | 0.498 | 4 (16.0%) | 7 (29.2%) | 0.384 | 6 (24.0%) | 1 (4.2%) | 0.127 |
|  | nil | 15 (60.0%) | 18 (75.0%) |  | 18 (72.0%) | 16 (66.7%) |  | 12 (48.0%) | 16 (66.7%) |  |
|  | ≥10 increase | 4 (16.0%) | 3 (12.5%) |  | 3 (12.0%) | 1 (4.2%) |  | 7 (28.0%) | 7 (29.2%) |  |

|  |  | H&N35: Feeding tube | | | H&N35: Weight loss | | | H&N35: Weight gain | | |
| --- | --- | --- | --- | --- | --- | --- | --- | --- | --- | --- |
|  | Score change | CMW (Tx) | SoC (Cx) |  | CMW (Tx) | SoC (Cx) |  | CMW (Tx) | SoC (Cx) |  |
|  |  | N (%) | N (%) | p | N (%) | N (%) | p | N (%) | N (%) | p |
| 1w | ≥10 decrease | 1 (3.3%) | 1 (3.1%) | 0.963 | 6 (20.0%) | 8 (25.0%) | 0.326 | 5 (16.7%) | 3 (9.4%) | 0.692 |
|  | nil | 29 (96.7%) | 31 (96.9%) |  | 21 (70.0%) | 17 (53.1%) |  | 20 (66.7%) | 23 (71.9%) |  |
|  | ≥10 increase | 0 (0.0%) | 0 (0.0%) |  | 3 (10.0%) | 7 (21.9%) |  | 5 (16.7%) | 6 (18.8%) |  |
| 2w | ≥10 decrease | 0 (0.0%) | 1 (3.1%) | 0.251 | 5 (17.9%) | 8 (25.0%) | 0.784 | 5 (17.9%) | 7 (21.9%) | 0.501 |
|  | nil | 1 (3.3%) | 1 (3.1%) |  | 15 (53.6%) | 15 (46.9%) |  | 21 (75.0%) | 20 (62.5%) |  |
|  | ≥10 increase | 0 (0.0%) | 2 (6.3%) |  | 8 (28.6%) | 9 (28.1%) |  | 2 (7.1%) | 5 (15.6%) |  |
| 3w | ≥10 decrease | 0 (0.0%) | 1 (2.9%) | 0.368 | 1 (3.0%) | 4 (11.8%) | 0.396 | 3 (9.1%) | 6 (17.6%) | 0.513 |
|  | nil | 33 (100.0%) | 32 (94.1%) |  | 18 (54.5%) | 17 (50.0%) |  | 28 (84.8%) | 27 (79.4%) |  |
|  | ≥10 increase | 0 (0.0%) | 1 (2.9%) |  | 14 (42.4%) | 13 (38.2%) |  | 2 (6.1%) | 1 (2.9%) |  |
| 4w | ≥10 decrease | 1 (3.0%) | 1 (3.3%) | 0.792 | 4 (12.1%) | 5 (16.7%) | 0.790 | 3 (9.1%) | 5 (16.7%) | 0.485 |
|  | nil | 31 (93.9%) | 27 (90.0%) |  | 11 (33.3%) | 8 (26.7%) |  | 24 (72.7%) | 22 (73.3%) |  |
|  | ≥10 increase | 1 (3.0%) | 2 (6.7%) |  | 18 (54.5%) | 17 (56.7%) |  | 6 (18.2%) | 3 (10.0%) |  |
| 5w | ≥10 decrease | 1 (3.0%) | 1 (3.1%) | 0.999 | 1 (3.0%) | 2 (6.3%) | 0.241 | 3 (9.1%) | 5 (15.6%) | 0.471 |
|  | nil | 31 (93.9%) | 30 (93.8%) |  | 17 (51.5%) | 10 (31.3%) |  | 27 (81.8%) | 26 (81.3%) |  |
|  | ≥10 increase | 1 (3.0%) | 1 (3.1%) |  | 15 (45.5%) | 20 (62.5%) |  | 3 (9.1%) | 1 (3.1%) |  |
| 6w | ≥10 decrease | 0 (0.0%) | 1 (3.7%) | 0.164 | 0 (0.0%) | 3 (11.1%) | 0.025 | 5 (16.7%) | 5 (18.5%) | 0.933 |
|  | nil | 29 (96.7%) | 22 (81.5%) |  | 14 (46.7%) | 5 (18.5%) |  | 22 (73.3%) | 20 (74.1%) |  |
|  | ≥10 increase | 1 (3.3%) | 4 (14.8%) |  | 16 (53.3%) | 19 (70.4%) |  | 3 (10.0%) | 2 (7.4%) |  |
| 7w | ≥10 decrease | 20 (95.2%) | 13 (100.0%) | 0.425 | 0 (0.0%) | 2 (15.4%) | 0.061 | 2 (9.5%) | 0 (0.0%) | 0.409 |
|  | nil | 1 (4.8%) | 0 (0.0%) |  | 7 (33.3%) | 1 (7.7%) |  | 16 (76.2%) | 12 (92.3%) |  |
|  | ≥10 increase | 0 (0.0%) | 0 (0.0%) |  | 14 (66.7%) | 10 (76.9%) |  | 3 (14.3%) | 1 (7.7%) |  |
| 1m | ≥10 decrease | 0 (0.0%) | 1 (2.8%) | 0.622 | 3 (13.0%) | 7 (19.4%) | 0.731 | 4 (17.4%) | 4 (11.1%) | 0.737 |
|  | nil | 20 (87.0%) | 32 (88.9%) |  | 11 (47.8%) | 14 (38.9%) |  | 14 (60.9%) | 22 (61.1%) |  |
|  | ≥10 increase | 3 (13.0%) | 3 (8.3%) |  | 9 (39.1%) | 15 (41.7%) |  | 5 (21.7%) | 10 (27.8%) |  |
| 3m | ≥10 decrease | 1 (4.0%) | 0 (0.0%) | 0.368 | 5 (20.0%) | 4 (16.7%) | 0.732 | 4 (16.0%) | 2 (8.3%) | 0.712 |
|  | nil | 23 (92.0%) | 24 (100.0%) |  | 16 (64.0%) | 14 (58.3%) |  | 15 (60.0%) | 16 (66.7%) |  |
|  | ≥10 increase | 1 (4.0%) | 0 (0.0%) |  | 4 (16.0%) | 6 (25.0%) |  | 6 (24.0%) | 6 (25.0%) |  |
